# Microarray profiling reveals suppressed interferon stimulated gene program in fibroblasts from scleroderma-associated interstitial lung disease

**DOI:** 10.1186/1465-9921-14-80

**Published:** 2013-08-02

**Authors:** Gisela E Lindahl, Carmel JW Stock, Xu Shi-Wen, Patricia Leoni, Piersante Sestini, Sarah L Howat, George Bou-Gharios, Andrew G Nicholson, Christopher P Denton, Jan C Grutters, Toby M Maher, Athol U Wells, David J Abraham, Elisabetta A Renzoni

**Affiliations:** 1 Interstitial Lung Disease Unit, Royal Brompton Hospital and National Heart and Lung Institute, Imperial College London, Emmanuel Kaye Building, 1B Manresa Road, London SW3 6LR, UK; 2 Centre for Rheumatology and Connective Tissue Diseases, Royal Free Campus, University College London Medical School, London NW3 2PF, UK; 3 Respiratory Medicine Department, Ospedale “Le Scotte”, University of Siena, Siena, Italy; 4 Institute of Pharmaceutical Science, King’s College London, Franklin-Wilkins Building, 150 Stamford Street, London SE1 9NH, UK; 5 Kennedy Institute of Rheumatology, University of Oxford, 65 Aspenlea Road, London W6 8HL, UK; 6 Histopathology Department, Royal Brompton Hospital and National Heart and Lung Institute, Imperial College London, Emmanuel Kaye Building, 1B Manresa Road, London SW3 6LR, UK; 7 Center of Interstitial Lung Diseases, St. Antonius Hospital Nieuwegein, and Division of Heart and Lungs, University Medical Centre Utrecht, Utrecht, NL, Netherlands

**Keywords:** SSc-ILD, IPF, Pulmonary fibroblasts, Interferon regulated genes

## Abstract

**Background:**

Interstitial lung disease is a major cause of morbidity and mortality in systemic sclerosis (SSc), with insufficiently effective treatment options. Progression of pulmonary fibrosis involves expanding populations of fibroblasts, and the accumulation of extracellular matrix proteins. Characterisation of SSc lung fibroblast gene expression profiles underlying the fibrotic cell phenotype could enable a better understanding of the processes leading to the progressive build-up of scar tissue in the lungs. In this study we evaluate the transcriptomes of fibroblasts isolated from SSc lung biopsies at the time of diagnosis, compared with those from control lungs.

**Methods:**

We used Affymetrix oligonucleotide microarrays to compare the gene expression profile of pulmonary fibroblasts cultured from 8 patients with pulmonary fibrosis associated with SSc (SSc-ILD), with those from control lung tissue peripheral to resected cancer (n=10). Fibroblast cultures from 3 patients with idiopathic pulmonary fibrosis (IPF) were included as a further comparison. Genes differentially expressed were identified using two separate analysis programs following a set of pre-determined criteria: only genes significant in both analyses were considered. Microarray expression data was verified by qRT-PCR and/or western blot analysis.

**Results:**

A total of 843 genes were identified as differentially expressed in pulmonary fibroblasts from SSc-ILD and/or IPF compared to control lung, with a large overlap in the expression profiles of both diseases. We observed increased expression of a TGF-β response signature including fibrosis associated genes and myofibroblast markers, with marked heterogeneity across samples. Strongly suppressed expression of interferon stimulated genes, including antiviral, chemokine, and MHC class 1 genes, was uniformly observed in fibrotic fibroblasts. This expression profile includes key regulators and mediators of the interferon response, such as *STAT1*, and *CXCL10*, and was also independent of disease group.

**Conclusions:**

This study identified a strongly suppressed interferon-stimulated gene program in fibroblasts from fibrotic lung. The data suggests that the repressed expression of interferon-stimulated genes may underpin critical aspects of the profibrotic fibroblast phenotype, identifying an area in pulmonary fibrosis that requires further investigation.

## Background

Pulmonary fibrosis, characterised by the destruction of lung architecture leading to organ failure, is, together with pulmonary hypertension (PH), the leading cause of death in patients with systemic sclerosis (SSc) [1]. Interstitial lung disease is more common in SSc (SSc-ILD) than in any other connective tissue disease, occurring in > 70% of patients [2], and is most frequently associated with a pattern of non-specific interstitial pneumonia (NSIP) [3]. Despite intense research efforts, the underlying mechanisms of SSc-ILD remain largely unknown [4], and there are currently limited therapeutic options for this serious complication [2].

While a large number of hypothesis-driven studies have identified potential profibrotic mediators [4,5], translation of these into therapeutic targets has so far been largely disappointing [6]. The search for more effective targets in lung fibrosis is now being addressed using hypothesis generating microarray-based strategies [7,8]. The majority of these studies have investigated gene expression in whole lung tissue samples, mostly in the idiopathic setting [5]. Matrix metalloproteinase (MMP) 7 [9], osteopontin [10], Twist1 [11], and MMP19 [12], are among suggested mediators identified using this strategy in idiopathic pulmonary fibrosis (IPF), a disease characterised by a histological pattern of usual interstitial pneumonia (UIP) [13,14]. In SSc, most microarray studies have been performed on skin biopsies and dermal fibroblasts [7]. However, a recent study compared whole lung tissue and fibroblasts isolated, at the time of transplant, from SSc-ILD lungs with a histological pattern of UIP, with those from IPF and idiopathic PH. The investigators reported gene profiles of SSc-ILD/UIP, with either predominant fibrosis or PH, overlapping with profiles of IPF and idiopathic PH, respectively [15].

While the initiating factors for fibrosis development may vary between diseases, the progressive accumulation of scar tissue in the lung is characterised by common themes, including expanding populations of activated fibroblasts, and excessive accumulation of extracellular matrix (ECM) proteins [5]. An important strategy to identify potential therapeutic targets, therefore, is to define fibrotic fibroblast phenotypes so as to delineate underlying key mechanisms for fibrosis progression.

Here we report analysis of the transcriptome of fibroblasts isolated from surgical lung biopsies at the time of diagnosis, from patients with well defined SSc-ILD and the histopathological pattern of NSIP. Although the main aim of this study was to compare SSc-ILD/NSIP fibroblast gene expression profiles with those of control lung fibroblasts, we also included a small number of IPF-derived fibroblast lines, as a separate fibrotic group. Our study confirms, with a robust signature in both diseases, the aberrant expression of previously reported myofibroblast markers and fibrosis mediators, and identifies a number of novel, co-expressed putative disease targets. We also observed the suppression of a large gene program, the interferon stimulated genes (ISGs), reported here for the first time. From the known function of some of these genes [16], it is possible to hypothesise that this suppressed program underlies key fibrotic fibroblast properties, such as hyper-proliferation, and apoptosis resistance. This study therefore identifies a potential new area for investigation and possible intervention in pulmonary fibrosis.

## Methods

### Patients and primary lung fibroblasts

Primary adult pulmonary fibroblasts were cultured from control tissue samples of unaffected lung from patients undergoing cancer-resection surgery (n=10), and from surgical lung biopsy samples of 11 patients with pulmonary fibrosis (SSc-ILD n=8 and IPF n=3). Independent reviews of the clinical and histopathologic diagnoses were performed and conformed to established criteria [17,18]. All of the SSc-ILD biopsies were characterised by a fibrotic NSIP pattern, and the IPF biopsies by a UIP pattern, based on current consensus criteria for these histological patterns [19]. The control tissue was histologically normal. Median age (range) was 60 (52–78) in controls, 48 (38–69) in SSc-ILD, and 61 (44–67) in IPF. The gender distribution (M/F) was as follows: controls 6/4; SSc-ILD 2/6; IPF 2/1. Four of the SSc-ILD and two of the IPF patients were ex-smokers. Smoking status was not available for all control cases. In SSc-ILD patients, median (range) percent predicted FVC was 72.5% (61–106), median FEV1 was 79% (58–92) and median DLCO was 50% (24–58). In IPF patients, median FVC was 70% (64–75), median FEV1 was 66% (55–79), and median DLCO was 50% (35–53). Patients had not been on corticosteroids or other immunosuppressants prior to surgical biopsy, as the biopsy was performed at the time of diagnosis of the ILD pattern, prior to initiation of treatment. Informed written consent was given by all subjects, and authorisation given by the Royal Brompton Hospital Ethics Committee. Fibroblasts were obtained from the biopsies by explant culture, and cell cultures maintained, as previously described [20,21]. Experiments were performed on fibroblasts at passage 2–5. Only one sample (S1) was used at passage 2. There was no difference in the median passage number between the control (median: 4.5; range: 3–5), SSc-ILD (median: 4; range: 2–5) and IPF (median: 4; range 3–5) groups.

### Microarray gene expression and analysis

At confluence, lung fibroblasts were serum-deprived for 42 hours (media changed at 18 hours) in the presence of 0.1% bovine serum albumin (Sigma). Total RNA was harvested (Trizol, Life Technologies), quantified, and the integrity verified by denaturing gel electrophoresis. Samples with a 28S:18S ratios of approximately 2:1 were accepted for further analysis by the Genomics Laboratory, CSC-MRC, Imperial College London, Hammersmith. RNA samples were prepared for chip hybridisation following manufacturer’s guidelines (Affymetrix). Hybridisation of cRNA to Affymetrix human U133Av2 chips, containing approximately 14,500 well characterised human genes, signal amplification, and data collection were performed using an Affymetrix fluidics station and chip reader, according to manufacturer’s protocol. Array normalisation, using the invariant set normalisation method, and subsequent calculation of model-based expression values, was performed using DNA-Chip Analyzer (dChip) [22]. A list of differentially expressed genes was generated in dChip using fold change ≥2, difference in means ≥100, and p<0.05. Significance analysis of microarrays (SAM) v 4.0 [23] was also used to determine significantly differentially expressed genes with fold change ≥2, difference in means ≥100, delta =1, and a false discovery rate <0.01. Only genes identified as differentially expressed according to both programs were considered as different between groups. Microarray data has been deposited in the Gene Expression Omnibus database [24], accession number GSE40839. Although the main aim of this study was to assess global gene expression profiles in SSc-ILD compared to controls, for completeness we present the comparison between both SSc-ILD and IPF and controls separately in tables. dChip software was used for data visualisation and hierarchical average linkage clustering using Pearson’s correlation [22].

### Functional category analysis

Functional categories enriched in the differentially expressed genes were identified using the functional annotation and clustering tool of the Database for Annotation, Visualisation, and Integrated Discovery (DAVID) v 6.7 [25,26]. The probability that a Gene Ontology (GO) biological process term [27] is overrepresented was determined by a modified Fisher’s exact test, comparing the proportion of genes in the whole genome which are part of that GO term, to the proportion of the differentially expressed genes which are part of the same GO term, and was expressed as an EASE score. Clusters of overrepresented GO terms were then generated based on the similarity of differentially expressed genes assigned to each functional GO term. Clusters were considered significantly overrepresented if they contained a minimum of five GO terms with an EASE score of ≤ 0.01. A summary description of each cluster was generated based on the constitutive GO term names of that cluster which achieved an EASE score <0.05 following Benjamini-Hotchberg correction of multiple comparisons. Only clusters with enrichment scores >3 (minus log transformed geometric mean of the EASE scores of the constitutive terms, equivalent to average EASE score=0.001) were selected. The open access database INTERFEROME [28] was used to identify differentially expressed genes which have been shown experimentally to be regulated by interferons.

### qRT-PCR

RNA was extracted using the RNeasy® Mini kit (Qiagen) according to manufacturer’s instructions. Samples were quantified and quality assessed using the NanoDrop spectrophotometry system (Thermo Scientific). Complementary DNA (cDNA) was synthesised from 500 ng RNA in a 20 μl reaction using the QuanTitect® reverse transcription kit (Qiagen). Expression levels were measured using a Rotor Gene 6000 (Corbett) in 10 μl reactions containing 2 μl cDNA (five-fold dilution), 1 × SensiMix™ SYBR NO-ROX (Bioline), and 0.5 μM of each forward and reverse primer (Table 1). PCR conditions were: 10 minutes at 95°C, followed by 40 cycles of 10 seconds at 95°C, 15 seconds at 57°C, and 5 seconds at 72°C. All reactions were performed in duplicate and non-template controls were included for each gene. Standard curves were generated for each gene studied using seven two-fold serial dilutions, high standard of 1×10^7^ copies/μl, of primer set amplicons generated from cDNA. Threshold cycle was manually determined at a fixed value of 10^-0.5^ and the template quantity calculated using Rotor Gene 6000 Series Software 1.7 (Corbett). Expression levels were normalised to *YWHAZ* and *HPRT1*[29,30].

**Table 1:** qRT-PCR primers

**Gene**	**Forward primer**	**Reverse primer**
**Normalisation genes**		
*HPRT1*	TGACACTGGCAAAACAATGCA	GGTCCTTTTCACCAGCAAGCT
*YWHAZ*	ACTTTTGGTACATTGTGGCTTCAA	CCGCCAGGACAAACCAGTAT
**Genes of interest**		
*CXCL10*	GAAAGCAGTTAGCAAGGAAAG	ATCCTTGGAAGCACTGCATC
*ID1*	CCAGAACCGCAAGGTGAG	GGTCCCTGATGTAGTCGATGA
*IFITM1*	TTCTTGAACTGGTGCTGTCT	ATGAGGATGCCCAGAATCAG
*IL11*	CCTGTGGGGACATGAACTGT	AGGGTCTGGGGAAACTCG
*IRF1*	CAGCCCAAGAAAGGTCCTC	TTGAACGGTACAGACAGAGCA
*NOX4*	CTGCTGACGTTGCATGTTTC	CGGGAGGGTGGGTATCTAA
*Serpine 1*	GGAAAGGCAACATGACCAG	CAGGTTCTCTAGGGGCTTCC
*STAT1*	GGATCAGCTGCAGAACTGGT	TTTCTGTTCCAATTCCTCCAA

Shown are the primers, written 5’ to 3’, used for measuring expression levels by qRT-PCR for validation of the microarray results. All primer pairs except for *YWHAZ, IRF1,* and *ID1*, are intron spanning.

### Western blot analysis

Following pre-incubation for 24 hours in serum-free media (DMEM, 0.1% BSA, penicillin/streptomycin), pulmonary fibroblasts from healthy controls, SSc-ILD, and IPF (n=3 for each phenotype), were cultured for a further 24 hours in fresh serum-free media. Cells were lysed and western blot analysis was performed using the following primary antibodies: CTGF and STAT1 (Santa Cruz Biotechnology); αSMA (Dako); IFITM1-3, ISG15 and GAPDH (Abcam); IRF-1 (Cell Signaling Technology); horseradish peroxidise conjugated secondary antibodies (Dako and Cell Signaling Technology); and ECL detection (Amersham).

## Results

### Gene expression profiles of fibrotic lung fibroblasts: approximately two-thirds of differentially expressed genes are down-regulated

**Figure 1:**
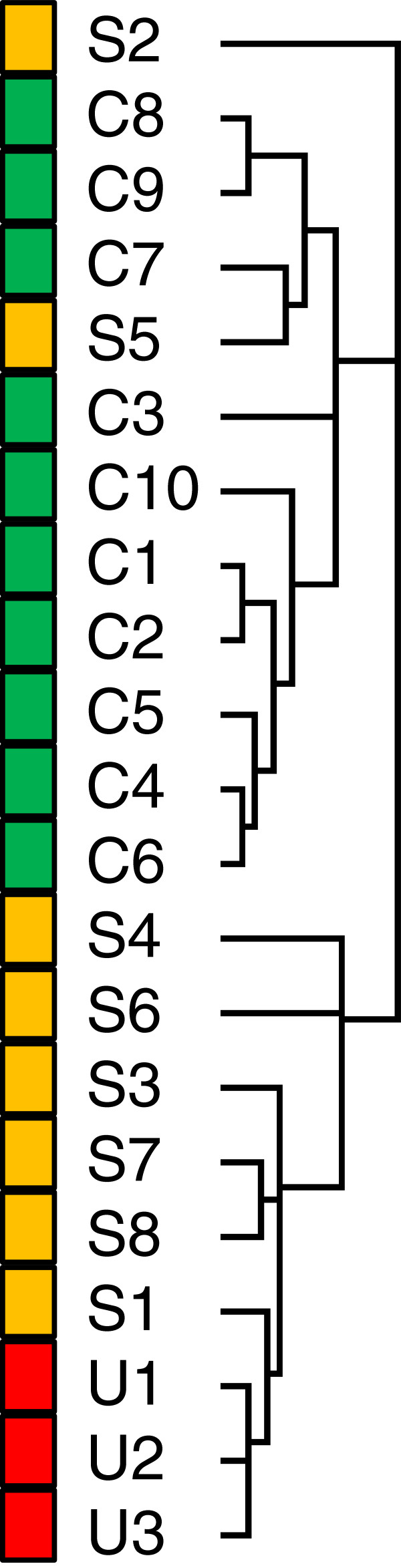
**Unsupervised clustering of samples based on full microarray probe set.** The sample dendrogram resulting from hierarchical clustering using all 22 K probes, shows clustering of samples by phenotype: control (C, green bar), SSc-ILD (S, orange bar), IPF (U, red bar).

Using an Affymetrix platform (U133Av2), we determined basal (serum free) global gene expression levels in fibroblasts prepared from lung tissue of 8 patients with SSc-ILD and 10 control lungs. As a further comparison we also included 3 fibroblast cultures from lung tissue of IPF patients. Unsupervised hierarchical cluster analysis of samples and genes resulted in an overall separation of fibrotic samples from controls (Figure 1). Two of the SSc-ILD samples clustered among the normal controls, demonstrating recognised fibrotic fibroblast sample heterogeneity.

When stringent criteria were applied, as described in the methods section, 478 and 744 probe sets (probes in future), equivalent to 360 and 547 genes, displayed differential expression by at least two-fold in SSc-ILD and IPF fibroblasts, respectively, compared to control fibroblasts. In SSc-ILD fibroblasts, 125 probes (99 genes) showed significantly higher, and 353 probes (261 genes) lower expression levels compared with controls (Additional file 1). In IPF fibroblasts, 239 and 505 probes (181 and 366 genes) had significantly higher and lower expression, respectively, compared with control fibroblasts (Additional file 2). Thus, approximately two thirds of differentially expressed genes were expressed at lower levels in fibroblasts from the two disease groups compared with controls. The sets of underexpressed genes in the disease groups, compared to normal controls, also contained the most significantly differentially expressed genes. Table 2 lists the 20 most significantly differentially expressed, and highest ranking (fold change) genes in SSc-ILD and IPF samples, separately, versus controls. It should be noted here that, while 379 out of a total of 843 probes were differentially expressed in both SSc-ILD and IPF vs. controls, most genes falling outside this overlap were very nearly significant in the other disease group, i.e. followed similar trends without meeting cut-off criteria. When the two disease groups were compared directly, only 8 probes (7 genes) were significantly differentially expressed (Additional file 3). The 843 probes differentially expressed in SSc-ILD and/or IPF, which also include the genes differentially expressed between these two groups, are listed in Additional file 4. Within the pooled fibrotic samples (IPF and SSc-ILD), no significant difference was observed in the 843 probes according to smoking status (SAM analysis, data not shown).

**Table 2:** Most differentially expressed genes in SSc-ILD and IPF compared to controls

**Genes overexpressed in SSc-ILD vs. Control**	**Probe set ID**	**Control mean**	**SSc-ILD mean**	**Fold change**	**p value**
Inhibitor of DNA binding 1, dominant negative helix-loop-helix protein	208937_s_at	25.5	917.5	36.1	0.00078
Interleukin 11	206924_at	23.6	717.8	30.4	0.015
Inhibitor of DNA binding 3, dominant negative helix-loop-helix protein	207826_s_at	27.7	603.2	21.8	0.00051
Tetraspanin 13	217979_at	37.9	533.9	14.1	0.0033
Elastin	212670_at	43.7	396.9	9.1	0.0021
Xylosyltransferase I	213725_x_at	29.0	255.4	8.8	0.0024
Serpin peptidase inhibitor, clade E, member 1	202628_s_at	329.8	2473.2	7.5	0.0022
Basic helix-loop-helix family, member e40	201170_s_at	44.3	256.1	5.8	0.0014
Connective tissue growth factor	209101_at	467.9	2637.1	5.6	0.00068
Solute carrier family 7, member 5	201195_s_at	56.1	294.2	5.3	0.00022
Tropomyosin 1 (alpha)	206116_s_at	317.5	1619.6	5.1	0.0032
Phosphoribosyl pyrophosphate synthetase 1	208447_s_at	59.0	283.4	4.8	0.0036
Inhibin, beta A	210511_s_at	143.3	688.1	4.8	0.0012
Growth arrest and DNA-damage-inducible, beta	207574_s_at	68.1	306.6	4.5	0.0023
Coiled-coil domain containing 99	221685_s_at	85.1	373.3	4.4	0.0018
Cadherin 2, type 1, N-cadherin (neuronal)	203440_at	105.3	433.0	4.1	0.00095
Desmoplakin	200606_at	80.5	306.1	3.8	0.0016
Insulin-like growth factor binding protein 3	212143_s_at	408.6	1489.1	3.6	0.0068
Microtubule associated monoxygenase, calponin and LIM domain containing 2	212473_s_at	184.7	667.5	3.6	0.0039
Prostaglandin-endoperoxide synthase 1	215813_s_at	102.4	362.0	3.5	0.0083
**Genes underexpressed in SSc-ILD vs. Control**	**Probe set ID**	**Control mean**	**SSc-ILD mean**	**Fold change**	**p value**
Chemokine (C-X-C motif) ligand 10	204533_at	771.2	19.2	−40.1	0.00034
Chemokine (C-X-C motif) ligand 11	210163_at	179.9	5.0	−36.0	0.0027
Flavin containing monooxygenase 2 (non-functional)	211726_s_at	530.4	15.7	−33.7	0.017
Interferon-induced protein with tetratricopeptide repeats 2	217502_at	707.1	26.1	−27.1	0.0096
Vascular cell adhesion molecule 1	203868_s_at	835.2	32.2	−26.0	0.0049
Bone marrow stromal cell antigen 2	201641_at	315.8	12.5	−25.3	0.0081
Radical S-adenosyl methionine domain containing 2	213797_at	333.8	13.3	−25.1	0.0039
Interferon-induced protein 44-like	204439_at	370.7	15.4	−24.1	0.00033
Interferon-induced protein with tetratricopeptide repeats 1	203153_at	1744.2	82.6	−21.1	0.000039
2’,5’-oligoadenylate synthetase 1, 40/46 kDa	205552_s_at	374.6	18.3	−20.5	0.0014
Complement factor B	202357_s_at	837.0	42.6	−19.6	0.0036
Interferon-induced protein with tetratricopeptide repeats 3	204747_at	985.0	61.9	−15.9	0.00031
Chromosome 10 open reading frame 10	209183_s_at	208.8	13.6	−15.3	0.0062
Myxovirus resistance 1, interferon-inducible protein p78 (mouse)	202086_at	1361.9	91.2	−14.9	0.00012
Receptor (chemosensory) transporter protein 4	219684_at	196.4	13.3	−14.8	0.000091
Chemokine (C-C motif) ligand 11	210133_at	529.8	36.2	−14.6	0.0021
Retinoic acid receptor responder (tazarotene induced) 3	204070_at	239.4	17.2	−13.9	0.00024
Alcohol dehydrogenase 1B (class I), beta polypeptide	209613_s_at	268.9	19.8	−13.6	0.011
Secreted and transmembrane 1	213716_s_at	285.0	22.0	−13.0	0.0012
Interferon, alpha-inducible protein 6	204415_at	1196.6	93.7	−12.8	0.00043
**Genes overexpressed in IPF vs. Control**	**Probe set ID**	**Control mean**	**IPF mean**	**Fold change**	**p value**
Interleukin 11	206924_at	23.6	2374.9	100.6	0.0019
Inhibitor of DNA binding 1, dominant negative helix-loop-helix protein	208937_s_at	25.5	752.8	29.6	0.011
Tetraspanin 13	217979_at	37.9	1039.1	27.4	0.0052
NADPH oxidase 4	219773_at	12.3	323.6	26.4	0.016
Inhibitor of DNA binding 3, dominant negative helix-loop-helix protein	207826_s_at	27.7	603.4	21.8	0.0025
Phospholamban	204939_s_at	25.7	460.1	17.9	0.035
Elastin	212670_at	43.7	766.9	17.6	0.0026
Xylosyltransferase I	213725_x_at	29.0	443.6	15.3	0.034
Galanin prepropeptide	214240_at	18.4	254.4	13.8	0.014
Cytokine receptor-like factor 1	206315_at	23.4	319.3	13.6	0.0098
Calponin 1, basic, smooth muscle	203951_at	83.0	1048.0	12.6	0.0024
Follistatin-like 3	203592_s_at	32.5	404.8	12.4	0.0048
CTP synthase	202613_at	39.5	454.7	11.5	0.000059
Endothelial cell-specific molecule 1	208394_x_at	10.2	116.8	11.4	0.031
Cadherin 6, type 2, K-cadherin (fetal kidney)	210602_s_at	26.2	298.4	11.4	0.00092
Proenkephalin	213791_at	33.7	366.7	10.9	0.00086
Adhesion molecule with Ig-like domain 2	222108_at	54.7	490.6	9.0	0.025
NUAK family, SNF1-like kinase, 1	204589_at	56.2	489.9	8.7	0.001
Tropomyosin 1 (alpha)	206117_at	30.9	261.7	8.5	0.0069
Inhibin, beta A	210511_s_at	143.3	1198.5	8.4	0.0023
**Genes underexpressed in IPF vs. Control**	**Probe set ID**	**Control mean**	**IPF mean**	**Fold change**	**p value**
Interferon-induced protein with tetratricopeptide repeats 1	203153_at	1744.2	5.4	−321.4	0.000033
Myxovirus resistance 1, interferon-inducible protein p78 (mouse)	202086_at	1361.9	8.8	−154.1	0.000096
Interferon, alpha-inducible protein 6	204415_at	1196.6	11.8	−101.2	0.00027
Chemokine (C-X-C motif) ligand 10	204533_at	771.2	9.5	−81.3	0.00031
Superoxide dismutase 2, mitochondrial	221477_s_at	2180.4	29.8	−73.2	<0.000001
Myxovirus resistance 2 (mouse)	204994_at	517.3	8.5	−60.6	0.00062
Interferon induced transmembrane protein 1 (9–27)	214022_s_at	3698.0	61.4	−60.3	<0.000001
Interferon-induced protein with tetratricopeptide repeats 3	204747_at	985.0	17.2	−57.2	0.00023
Pentraxin 3, long	206157_at	1415.2	35.2	−40.2	0.000016
Interferon-induced protein 44-like	204439_at	370.7	11.6	−31.9	0.0003
Complement component 3	217767_at	457.9	14.4	−31.8	0.000072
KIAA1199	212942_s_at	799.0	30.8	−26.0	0.00027
Interferon-induced protein 35	209417_s_at	455.6	18.1	−25.1	0.000062
Chemokine (C-X-C motif) ligand 1	204470_at	637.1	26.3	−24.2	<0.000001
Growth arrest-specific 1	204457_s_at	383.2	17.2	−22.3	0.000059
Signal transducer and activator of transcription 1, 91 kDa	209969_s_at	327.3	15.3	−21.4	0.000097
Chemokine (C-C motif) ligand 2	216598_s_at	2676.4	128.3	−20.9	0.00003
Interferon-induced protein 44	214453_s_at	335.0	17.0	−19.7	0.000027
Caspase 1 (interleukin 1, beta, convertase)	211367_s_at	180.6	10.0	−18.1	<0.000001
Tumor necrosis factor, alpha-induced protein 2	202510_s_at	404.2	22.6	−17.9	0.000003

Shown are the top 20 genes, based on fold change, over and under expressed in SSc-ILD and IPF compared to controls. Where more than one probe set corresponding to the same gene were present in the top 20, the probe with the greatest fold change or most significant p-value, is shown.

**Figure 2:**
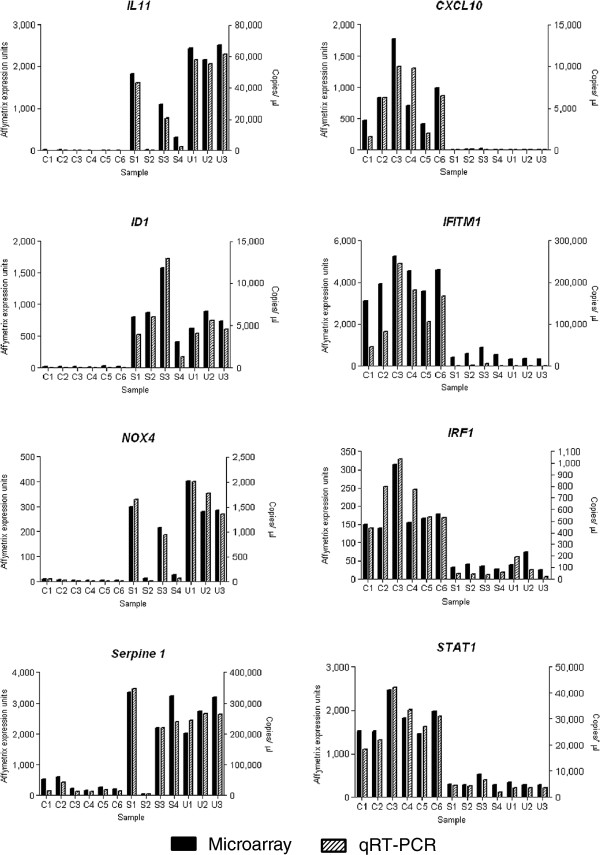
**Quantitative RT-PCR confirmation of microarray results.** Expression levels of eight genes selected from the microarray data was measured by qRT-PCR in thirteen of the samples used in the microarray. For each sample the microarray data is plotted on the left-hand axis, and the qRT-PCR results plotted on the right-hand axis. qRT-PCR expression levels were normalised to *YWHAZ* and *HPRT1.*

**Figure 3:**
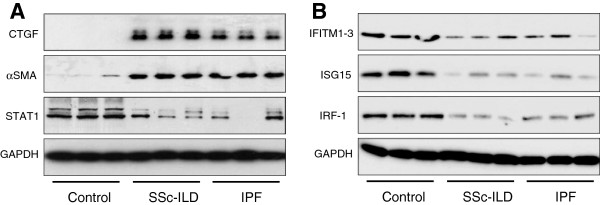
**Western blot confirmation of microarray results.** Protein expression levels of six significantly differently expressed genes selected from the microarray data were visualised by western blot in fibroblast samples independent to those used in the microarray (n=3 for each group). **A)** Protein expression of fibrosis related genes, CTGF and αSMA; and key regulator of interferon response, STAT1. **B)** Protein expression of interferon stimulated genes (ISG): IFITM1-3, ISG15 and IRF-1.

Expression levels of a subset of genes identified by the present microarray analysis were verified by qRT-PCR in the microarray RNA samples demonstrating good correlation between these two techniques (Figure 2). Protein levels for six differentially expressed genes, fibrosis related genes; connective tissue growth factor (CTGF*)*, and alpha-smooth muscle actin (αSMA), and interferon stimulated genes (ISG); signal transducer and activator of transcription 1 (STAT1), IFITM1-3 (all three isoforms are detected by this antibody), ISG15 and IRF-1, were determined by western blot analysis in independent preparations of additional SSc-ILD, IPF, and control fibroblasts (Figure 3).

### Functional groups over-represented among differentially expressed genes

As predicted from the similarities between the two disease groups with regards to differentially expressed genes, gene ontology analysis revealed a major overlap in overrepresented functional groups. Enriched functional groups representing six broad categories (GO clusters) were identified among the genes with higher expression in disease fibroblasts compared with normal controls (see Table 3 for a summary of enriched clusters of GO terms, and Additional file 5 for full GO analysis): anatomical structure development, regulation of cell cycle, response to stress and wounding, regulation of apoptosis, cell migration, and smooth muscle contraction. Among underexpressed/downregulated genes, GO clusters included: inflammatory and immune response, response to biotic stimulus, regulation of apoptosis, regulation of cell migration, regulation of cell proliferation, and regulation of I-κB/NF-κB cascade. By far the most enriched functional groups, compared with control cells, in both SSc-ILD and IPF lung fibroblasts, are genes involved in immune system processes and in microbial/viral defence, which are strongly suppressed in both disease groups. These genes are also among the most significantly differentially expressed genes in this study.

**Table 3:** Over-represented functional terms

**GO cluster**	**Description**	**Enrichment score**
**Genes overexpressed in SSc-ILD vs. Control**		
1	Anatomical structure development	5.50
2	Regulation of cell cycle	4.23
3	Response to stress and wounding	3.33
**Genes underexpressed in SSc-ILD vs. Control**		
1	Inflammatory response	11.53
2	Regulation of cell proliferation	5.16
3	Regulation of biological process	5.12
4	Inflammatory response/chemotaxis	4.81
5	Regulation of cell migration	4.33
6	Response to external stimulus	4.19
7	Regulation of apoptosis	3.78
8	Inflammatory and immune response	3.77
9	Response to biotic stimulus/ion homeostasis	3.69
10	Regulation of I-κB kinase/NF-κB cascade	3.37
**Genes overexpressed in IPF vs. Control**		
1	Anatomical structure development/neurogenesis	4.94
2	Regulation of apoptosis	3.95
3	Cell migration/neurogenesis	3.81
4	Regulation of cell motion	3.45
5	Response to wounding/tissue development	3.17
6	Smooth muscle contraction/Blood circulation	3.03
**Genes underexpressed in IPF vs. Control**		
1	Immune response	12.65
2	Response to virus, bacteria and LPS	8.66
3	Positive regulation of biological process and cell death	5.29
4	Negative regulation of biological process and cell death	4.91
5	Regulation of immune system and developmental process	4.08
6	Inflammatory response/chemotaxis	3.98
7	Regulation of cell migration and adhesion	3.87
8	Inflammatory and humoral immune response	3.63
9	Anatomical structure development	3.39
10	Response to stimulus and I-κB kinase/NF-κB cascade	3.19
11	Response to extracellular stimulus and oxidative stress	3.18

Using the DAVID functional annotation tool, genes over and under expressed in SSc-ILD and IPF compared to controls were clustered according to Gene Ontology (GO) biological process terms. Shown are the summary descriptions and enrichment scores of the sets of enriched GO terms within each GO cluster with an enrichment score >3.

### Cluster analysis of differentially expressed genes – ISGs; a major group suppressed in fibrotic lung fibroblasts

**Figure 4:**
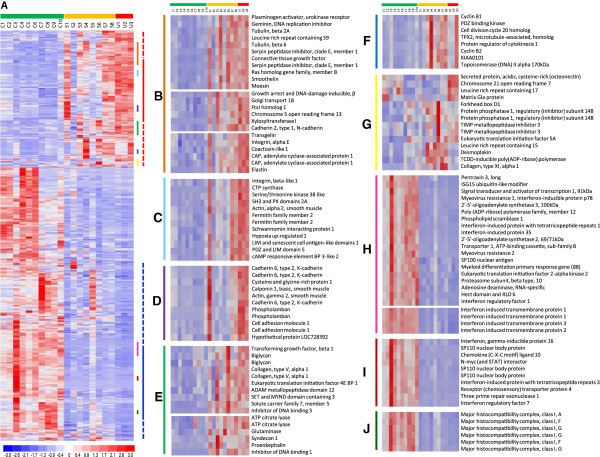
**Gene expression profiles of control, SSc-ILD, and IPF pulmonary fibroblasts. A)** Supervised hierarchical clustering of probes. The gene set used comprised the 843 probes with a fold change ≥2, difference in means ≥100, and p<0.05 (dChip) and FDR<0.01 (SAM), in both the SSc-ILD or IPF samples, compared to controls, which includes also 8 probes differentially expressed between SSc-ILD and IPF samples when compared directly. Each column corresponds to an individual sample (C= control, S=SSc-ILD, U=IPF), and each row corresponds to an individual probe. The coloured bars to the right of the heatmap identify the location of the insets displayed in **B-J**. Panels **B-J** were selected to highlight sample heterogeneity and/or genes co-expressed with high scoring differentially expressed genes.

To visualise the differential expression across samples and to identify co-expressed genes, average linkage cluster analysis was performed using expression data for the 843 probes sets which displayed differential expression in at least one of the two comparisons: SSc-ILD vs. control, and IPF vs. control (Figure 4A). Within this set are also the eight probes differentially expressed when SSc-ILD and IPF samples were compared directly. Parts of identified gene clusters were selected to illustrate co-expression among upregulated (Figure 4, Panels B-F) and downregulated (Panels H-J) genes, and also to highlight different patterns of sample heterogeneity. Overall, a heterogeneous expression pattern was observed in the upregulated genes, whereas downregulated genes had more uniform expression patterns across samples for the majority of genes in both disease groups. Among upregulated genes, a TGF-β response signature [31,32] including genes encoding for fibrosis mediators and myofibroblast markers, such as *SERPINE1* (*PAI1*), connective tissue growth factor, smoothelin, and transgelin (*SM22*) is prominent (Panel B). Co-expressed with these are strongly upregulated genes: growth arrest and DNA-damage inducible β (*GADD45*), xylosyltransferase 1 (*XYLT1*), N-cadherin, and elastin, with potential roles in the fibrotic disease process. Groups B, C and D all contain genes involved in contraction and migration, however, the degree of heterogeneity between samples differ between these groups: in group B, the majority of fibrotic samples have elevated expression of smoothelin and transgelin compared with controls; in group C, fewer fibrotic samples, 7 out of 11, have enhanced levels of α2 smooth muscle actin (*ACTA2*) expression; and in group D, all three IPF samples, but only 3 out of 8 SSc-ILD samples, have elevated expression of calponin 1 and actin gamma 2 smooth muscle (*ACTG2)*. This may indicate different degrees of contractile/migratory phenotypes among these fibrotic cell preparations. Panel E contains *ID1* and *ID3*, which are in the top 20 differentially regulated genes in both disease groups, and are upregulated in most of the fibrotic samples. Group F depicts a cluster of co-expressed cell-cycle associated genes, including cyclins and *TOPO2*, which exhibit heterogeneous expression in both disease groups. Panel G illustrates an area with less clustering, which however includes possible disease specific genes, e.g. Secreted protein, acidic, cysteine-rich (*SPARC*) (IPF, Table 2B) and desmoplakin (SSc-ILD) (Additional file 4). Desmoplakin is among the top 20 most upregulated genes in SSc-ILD with an elevated expression in 7 out of the 8 SSc-ILD fibroblast lines, but with low expression in the three IPF cell preparations and in all controls. Desmoplakin is part of the desmosome complex which forms tight cell-cell contacts [33], and its enhanced expression in SSc-ILD fibroblasts may define a different pathogenesis and cell origin. Panels H-J shows the marked suppressed expression of interferon stimulated genes (ISGs), such as antiviral genes (Group H), chemokine (Group I) and MHC class I genes (Group J). This cluster also includes key regulators of the interferon gene program, *STAT1*, interferon regulatory factor 1 (*IRF1)* and interferon regulatory factor 7 (*IRF7)*, as well as chemokine (C-X-C motif) ligand 10 (*CXCL10*/*IP10*), one of the most strongly suppressed genes in the study, and the most strongly repressed chemokine in both disease groups (Panel H and I).

### Comparing the SSc-ILD and IFP fibroblast gene expression profiles with the interferome

Since we observe a clear fibrosis/TGF-β signature together with a strongly suppressed ISG program, and there is a well-documented antagonistic relationship between TGF-β and interferon signalling in the fibrosis literature [34], we next interrogated the Interferome, a database of interferon regulated genes (IRGs) reported in the literature [28]. This database includes 1996 human IRGs, of which 1581 are induced and 415 repressed by interferons. In our study, out of the 99 and 181 overexpressed genes in SSc-ILD and IPF fibroblasts, respectively, 40 (40.4%) and 55 (30.1%) genes were in the Interferome database. Among our underexpressed genes, out of a total of 261 and 366 in SSc-ILD and IPF fibroblasts, respectively, 134 (51.3%) and 173 (74.3%) genes were in the Interferome database. The genes overlapping with IRGs in this database are listed in Additional file 6. The comparison revealed that many of the TGF-β responsive genes upregulated in our microarray data set are indeed genes repressed by IFNs.

## Discussion

Previous studies have shown that fibroblasts isolated from SSc-ILD [35] and IPF [5] lungs, while displaying substantial heterogeneity, are generally more proliferative, migratory, resistant to apoptosis, and ECM producing, than control lung fibroblasts [36]. A greater proportion display elevated αSMA expression, also enhancing their contractility. These features are all consistent with the fibroblast accumulation and scar tissue formation observed in fibrotic lung. The so called myofibroblast phenotype is maintained over several passages in culture [37], suggesting an underlying epigenetic regulation, whether established and maintained in local cells within the chronic disease setting, or supporting the phenotype of a specialised infiltrating wound healing cell type [38]. Regardless of cell origin, this feature enables *in vitro* studies of mechanisms underlying the fibrotic fibroblast phenotype.

In this study we observed a high number of differentially expressed genes between SSc-ILD and/or IPF derived fibroblasts, compared with controls. There was a large overlap between expression profiles of SSc-ILD and IPF fibroblasts, suggesting several common pathways at this stage of the two diseases. Indeed, a direct comparison demonstrated only seven genes with significantly different expression between the two disease groups. Caution should be applied, however, when interpreting data from this direct comparison since only three IPF samples were included here. It is possible that more genes with differential expression between the two disease groups would be identified with a larger numbers of IPF fibroblasts samples. Such studies would also be required to verify the sporadic observations made in this study, such as the elevated expression levels of desmoplakin in SSc-ILD, but not in IPF fibroblasts. Therefore, we stress that the main objective of the study presented here was to gain an overview of potential SSc-ILD target genes, and the study was not designed to detect differences between different fibrotic entities.

Among significantly upregulated genes in both SSc-ILD and IPF/UIP fibroblasts, we identify a recognised fibrosis signature, including smooth muscle actin (*ACTA2*), *CTGF,* and *PAI1* (*SERPINE1*), along with genes more recently associated with lung fibrogenesis, including *ID1*, *ID3*, *IL11*, and *NOX4*. Inhibitor of DNA binding 1 and 3 (*ID1* and *ID3)*, are target genes of bone morphogenetic proteins, and control cell differentiation by dominant negative inhibition of helix-loop-helix transcription factors [39]. Chambers et al. described upregulation of *ID1* in lung fibroblasts in response to TGF-β, and linkage to the smooth muscle phenotypic switch [31]. Increased *ID1* and *ID3* gene expression was also observed in lung tissue and fibroblasts from patients with SSc-ILD by Hsu and colleagues, [15]. The ID proteins are overexpressed in many cancers, controlling cell growth and apoptosis, and have been suggested as a therapeutic target [39,40]. In fibroproliferative stages of fibrosis, high *ID1/ID3* expression could maintain fibroblasts in a dedifferentiated, hyperproliferative, and apoptosis resistant state. Another significantly upregulated gene, though somewhat variably in SSc-ILD fibroblasts, is *NOX4*, encoding a member of the NADPH oxidase (NOX) proteins which generate superoxide by electron transfer to oxygen [41]. Through its involvement in TGF-β-induced fibroblast differentiation into myofibroblasts, two recent studies have suggested a role for NOX4 in IPF, where it is found to be overexpressed [41]. Specific NOX4 inhibitors are now being developed as possible antifibrotic agents [42]. Another potential novel target gene, *GADD45B*, highly induced and coexpressed with the fibrosis related genes in this study, is a pro-survival factor associated with stress-resistant tumours [43], and has been found to be upregulated in SSc skin biopsies [44].

The most striking of our observations is the strongly and uniformly repressed ISG profile in fibrotic fibroblasts. Among these are genes coding for: antiviral protein myxovirus (influenza virus) resistance 1 (*MX1*), interferon gamma inducible protein p16 (*IFI16*), 2’,5’-oligoadenylate synthetase 1 (*OAS1*), the chemokines chemokine (C-X-C motif) ligand 10 (*CXCL10/IP10*) and chemokine (C-C motif) ligand 11 (*CCL11*), and antigen presenting MHC I molecules. Key transcriptional regulators of this program, including *IRF1*, *IRF7*, and *STAT1* are also suppressed. To our knowledge, the reduction in expression of a large set of immune response/interferon related genes has not previously been described in either SSc-ILD or IPF derived fibroblasts, or other fibrotic lung diseases. Global gene expression was recently evaluated in scleroderma whole skin biopsies and matched dermal fibroblasts. While 26 genes were differentially expressed in both scleroderma whole skin and fibroblasts compared to controls, nine were found to be discordant [45]. Interestingly, the majority of the discordant genes were upregulated in skin biopsies but downregulated in fibroblasts, including the ISG genes we identify as repressed in lung fibroblasts, such as *MX1*, *IFI16*, intercellular adhesion molecule 1 (*ICAM1*), and *OAS1*. While the authors argue that the discordant genes are an indication of a scarce representativeness of skin fibroblast gene expression *in vivo*[45], in light of our data, it is likely that they were in fact observing the same phenomenon reported in the current study. As these are mainly interferon regulated genes, the discordance between fibroblasts and whole tissue gene expression could be explained by the known increase of immune cell populations in SSc skin, likely to be overexpressing immunoregulatory genes. The upregulation of *MX1*, *IRF7* and *STAT1* in PBMC from SSc patients would support this notion [46,47]. The downregulation of the ISG program both in SSc-ILD and in IPF fibroblasts suggests that this phenomenon relates to a local fibroblast specific, rather than systemic, profibrotic process, perhaps underpinned by a general susceptibility for tissue fibrosis, common to both diseases.

Additional support for our finding of an aberrantly regulated ISG program in fibrotic lung fibroblasts, comes from work on two of the signature genes from this group, *CXCL10* (*IP10*) and *STAT1*. IP10 levels were found to be downregulated in IPF lung fibroblasts by Keane et al. [48]. More recently, Coward et al. have shown, again in IPF fibroblasts, that epigenetic dysregulation involving both histone deacetylation and hypermethylation is responsible for targeted repression of *IP10*[49]. By contrast, Hsu et al. did not report a significant difference in expression in isolated lung fibroblasts [15], a difference which may relate to experimental design, as discussed below. Further support of a role of suppressed ISGs in pulmonary fibrosis comes from two animal models. Mice deficient in *IP10*[50], and in *STAT1*[51], displayed enhanced susceptibility to pulmonary fibrosis. Based on these studies, the suppressed ISG program indentified in fibroblasts isolated from SSc-ILD and IPF lung, as presented here, would support enhanced lung fibrosis progression through promoting fibroblast proliferation, migration, and apoptosis resistance. Interestingly, IFN-γ treatment in IPF has failed to show a benefit [52], and there was a suggestion of worse pulmonary outcomes in a study investigating treatment of SSc patients with IFN-α compared with placebo [53], with the latter providing indirect support for IFN-related mechanisms involved in organ-specific SSc complications. There could be several possible explanations for these disappointing results, including unexpected adverse effects through circulating cell populations. Activation of pathways downstream of systemically administered interferons is likely to have different direct and indirect effects depending on the cell type and tissue location. The findings shown here add important information to this complexity, and need to be investigated in future detailed mechanistic cell and animal studies.

The signatures observed in our study are remarkably strong, both in terms of fold difference and statistical significance. One possible reason for this is that lung tissue samples were obtained from biopsies at the time of diagnosis, when the disease may be at a relatively early or active wound healing stage, and when perhaps fibroblast proliferation (accumulation) and elastin synthesis, rather than contraction and collagen remodelling, dominate. This is in contrast to the study by Hsu et al., in which gene expression profiles were investigated in SSc lung tissue and fibroblasts from transplant, and therefore possible end-stage, material, also noted by the authors as a potential limitation [15]. Another possibility relates to differences in *in vitro* culture condition as we employed serum free media before harvest, similarly to others, including Coward et al. [49], as opposed to in low serum (0.5%) as applied by e.g. Hsu et al. [15]. Serum withdrawal, a form of cellular stress, may evoke the clear differential expression profiles observed in our study. Whereas in the fibrotic fibroblasts an anti-apoptotic survival gene program is maintained, which may be a result of the suppressed ISGs, this gene program may not be subject to repression in the normal fibroblasts.

While an accepted source of control tissue in studies of ILD [10,12], the use of control fibroblasts from cancer resected specimens, rather than from healthy control subjects (not available for this study), represents a potential limitation. Although obtained from areas of lung with normal histological appearance, differential gene expression in fibroblasts derived from lungs in which cancer has developed cannot be excluded. However, significant gene expression differences were observed in lung cancer associated fibroblasts compared to matched fibroblasts from areas of normal lung from the same patient, suggesting that the cancer associated phenotype of lung fibroblasts is regionally limited to the cancer stroma [54]. A further possible source of bias in gene expression is smoking history. Smoking has been shown to be associated with interstitial fibrotic changes [55], and is itself likely to cause changes in the expression of certain genes. We observed no significant differences among differentially expressed genes according to smoking status in the pooled fibrotic samples, suggesting that the observed changes were related to the fibrotic lung disease itself rather than to smoking. However, as subgroup numbers were small, and it was not possible to separately analyse the two fibrotic lung diseases, further studies are needed to carefully assess the contribution of smoking to gene expression changes in the context of fibrotic lung disease.

While the general hypothesis, that a repressed interferon stimulated gene program at least in part underpins the fibrotic fibroblast phenotype, will be tested in future studies, it is interesting to note the same phenomenon in several other clinical settings where hyperplasia and apoptosis resistance are key features; certain viruses, including high-risk human papillomaviruses (HPV), have evolved a mechanism to down-regulate ISGs in host cells as an immunoevasive strategy [56], and persistent HPV infection may lead to cervical cancer development; breast cancer metastasis is promoted by *IRF7* silencing [57]; fibroblasts from patients with Li-Fraumeni syndrome become spontaneously immortalised through the downregulation of interferon pathway genes [58]. Conversely, *IRF1* expression reverts the phenotype of oncogenically transformed fibroblasts [59], and IRF-1 enhancing drugs with tumour suppressing properties are currently being developed [60]. Many similar examples in the literature lead to questions about whether fibrosis is a pre-cancerous state [61]. The repressed, or aberrantly regulated, fibroblast specific interferon response network may therefore be a common necessary determinant allowing lung fibrosis progression to occur.

In summary, in this study comparing gene expression profiles of fibroblasts explanted from fibrotic lung tissue (SSc-ILD and IPF), with control fibroblasts from areas of normal lung, we observe: an overall elevated expression of previously reported fibrosis associated genes, with marked heterogeneity across samples; differentially regulated myofibroblast markers which correlate with the expression heterogeneity between samples; and a strongly suppressed interferon stimulated gene program, uniformly present across fibrotic samples. This suppressed gene program displays both the greatest significance and largest fold differences in expression in our data set. Similarly to functional findings in parallel fields, particularly cancer, this group of genes, and the suppression of their expression, could explain essential aspects of the profibrotic fibroblast phenotype. This hypothesis will need to be tested by future studies, with particular focus on epigenetic silencing as a potential underlying mechanism.

## Abbreviations

DAVID: Database for annotation, visualisation, and integrated discovery; ECM: Extracellular matrix; GO: Gene ontology; IPF: Idiopathic pulmonary fibrosis; IRG: Interferon regulated genes; ISG: Interferon stimulated gene; NSIP: Nonspecific interstitial pneumonia; PH: Pulmonary hypertension; SAM: Significance analysis of microarrays; SSc: Systemic sclerosis; SSc-ILD: Interstitial lung disease in SSc; UIP: Usual interstitial pneumonia.

## Competing interests

The authors declare that they have no competing interests.

## Authors’ contributions

GEL CJWS contributed to design, data generation, and data analysis of the study, and wrote and prepared the manuscript. XS performed all explant cell preparations, cell culture experiments and western blot analyses. PS contributed to data analysis and interpretation, and critical review of the manuscript. PL SLH contributed to the verification of the microarray data and valuable discussions. GG participated in the microarray work. AGN contributed human lung tissue samples and expert histopathological typing. CPD and JCG contributed patient material and information. DJA, CPD AUW TMM contributed to design and critical supervision of the study. EAR designed the study, performed experimental work and wrote the manuscript. All authors contributed to critical revision of the manuscript. All authors read and approved the final manuscript.

## Supplementary Material

Additional file 1 **Genes differentially expressed in SSc-ILD.** Word file, .txt extension. This data set contains all of the genes up- or down- regulated in SSc-ILD fibroblasts compared to control fibroblasts. Included are p-values from dChip analysis and q-values from SAM analysis. Click here for file

Additional file 2 **Genes differentially expressed in IPF.** Word file, .txt extension. This data set contains all of the genes up- or down- regulated in IPF fibroblasts compared to control fibroblasts. Included are p-values from dChip analysis and q-values from SAM analysis. Click here for file

Additional file 3 **Genes differentially expressed between IPF and SSc-ILD.** Word file, .txt extension. This data set contains all of the genes up- or down- regulated in IPF fibroblasts compared to SSc-ILD fibroblasts. Included are p-values from dChip analysis and q-values from SAM analysis. Click here for file

Additional file 4 **Summary of differentially expressed genes in SSc-ILD and IPF.** Word file, .txt extension. This data set contains all of the genes up- or down- regulated in fibroblasts from at least one disease group compared to control fibroblasts. Included are p-values from dChip analysis and q-values from SAM analysis. Click here for file

Additional file 5 **Functional annotation clustering analysis.** Excel file, .xlsx extension. Using the DAVID functional annotation tool, genes over and under expressed in SSc-ILD and IPF compared to controls were clustered according to Gene Ontology biological process terms. Shown are the enriched terms within each annotation cluster with an EASE score threshold of ≤ 0.01, and an initial group membership of 5. Click here for file

Additional file 6 **Differentially expressed genes present in Interferome.** Excel file, .xlsx extension. The data set contains all of the genes differentially expressed genes in SSc-ILD and IPF fibroblasts compared to control fibroblasts which have been shown experimentally to be regulated by interferons listed in the INTERFEROME database. Click here for file
