# Supplementary material for: Microarray profiling reveals suppressed interferon stimulated gene program in fibroblasts from scleroderma-associated interstitial lung disease
Source: Respir Res. 2013 Aug 2;14(1):80. doi: 10.1186/1465-9921-14-80 (PMC3750263; doi:10.1186/1465-9921-14-80)
Supplement: Additional file 1 — Genes differentially expressed in SSc-ILD. Word file, .txt extension. This data set contains all of the genes up- or down- regulated in SSc-ILD fibroblasts compared to control fibroblasts. Included are p-values from dChip analysis and q-values from SAM analysis. [file 1465-9921-14-80-S1.docx]

| **Annotation** | **Accession** | **Probe Set ID** | **Control mean** | **SSc-ILD mean** | **Fold change** | **p value** | **q-value(%)** |
| --- | --- | --- | --- | --- | --- | --- | --- |
| **Overexpressed in SSc-ILD** |  |  |  |  |  |  |  |
| Inhibitor of DNA binding 1, dominant negative helix-loop-helix protein | D13889 | 208937_s_at | 25.45 | 917.52 | 36.05 | 0.00078 | <0.01 |
| Interleukin 11 | NM_000641 | 206924_at | 23.62 | 717.79 | 30.39 | 0.015 | <0.01 |
| Inhibitor of DNA binding 3, dominant negative helix-loop-helix protein | NM_002167 | 207826_s_at | 27.70 | 603.21 | 21.78 | 0.00051 | <0.01 |
| Tetraspanin 13 | NM_014399 | 217979_at | 37.92 | 533.94 | 14.08 | 0.0033 | <0.01 |
| Elastin | AA479278 | 212670_at | 43.66 | 396.92 | 9.09 | 0.0021 | <0.01 |
| Xylosyltransferase I | AI693140 | 213725_x_at | 29.01 | 255.42 | 8.80 | 0.0024 | <0.01 |
| Serpin peptidase inhibitor, clade E, member 1 | NM_000602 | 202628_s_at | 329.82 | 2473.18 | 7.50 | 0.0022 | <0.01 |
| Serpin peptidase inhibitor, clade E, member 1 | AL574210 | 202627_s_at | 373.34 | 2453.13 | 6.57 | 0.0040 | <0.01 |
| Basic helix-loop-helix family, member e40 | NM_003670 | 201170_s_at | 44.27 | 256.07 | 5.78 | 0.0014 | <0.01 |
| Connective tissue growth factor | M92934 | 209101_at | 467.87 | 2637.13 | 5.64 | 0.00068 | <0.01 |
| Solute carrier family 7, member 5 | AB018009 | 201195_s_at | 56.08 | 294.19 | 5.25 | 0.00022 | <0.01 |
| Tropomyosin 1 (alpha) | NM_000366 | 206116_s_at | 317.53 | 1619.64 | 5.10 | 0.0032 | <0.01 |
| Phosphoribosyl pyrophosphate synthetase 1 | NM_002764 | 208447_s_at | 58.98 | 283.42 | 4.81 | 0.0036 | <0.01 |
| Inhibin, beta A | M13436 | 210511_s_at | 143.33 | 688.08 | 4.80 | 0.0012 | <0.01 |
| Growth arrest and DNA-damage-inducible, beta | NM_015675 | 207574_s_at | 68.13 | 306.59 | 4.50 | 0.0023 | <0.01 |
| Coiled-coil domain containing 99 | AF269167 | 221685_s_at | 85.14 | 373.33 | 4.38 | 0.0018 | <0.01 |
| Growth arrest and DNA-damage-inducible, beta | AF087853 | 209304_x_at | 75.76 | 319.52 | 4.22 | 0.0015 | <0.01 |
| Cadherin 2, type 1, N-cadherin (neuronal) | M34064 | 203440_at | 105.26 | 432.98 | 4.11 | 0.00095 | <0.01 |
| Tropomyosin 1 (alpha) | M19267 | 210987_x_at | 621.30 | 2528.40 | 4.07 | 0.0025 | <0.01 |
| Phosphoribosyl pyrophosphate synthetase 1 | BC001605 | 209440_at | 141.67 | 548.77 | 3.87 | 0.0021 | <0.01 |
| Desmoplakin | NM_004415 | 200606_at | 80.51 | 306.14 | 3.80 | 0.0016 | <0.01 |
| Insulin-like growth factor binding protein 3 | BF340228 | 212143_s_at | 408.59 | 1489.10 | 3.64 | 0.0068 | <0.01 |
| Microtubule associated monoxygenase, calponin and LIM domain containing 2 | BE965029 | 212473_s_at | 184.67 | 667.48 | 3.61 | 0.0039 | <0.01 |
| Microtubule associated monoxygenase, calponin and LIM domain containing 2 | BE965029 | 212472_at | 95.31 | 341.04 | 3.58 | 0.0016 | <0.01 |
| Prostaglandin-endoperoxide synthase 1 | S36219 | 215813_s_at | 102.39 | 362.03 | 3.54 | 0.0083 | <0.01 |
| Ubiquitin-conjugating enzyme E2S | NM_014501 | 202779_s_at | 106.80 | 377.09 | 3.53 | 0.0087 | <0.01 |
| Ras homolog gene family, member B | AI263909 | 212099_at | 86.54 | 303.60 | 3.51 | 0.00099 | <0.01 |
| Actin, alpha 2, smooth muscle, aorta | NM_001613 | 200974_at | 820.33 | 2831.14 | 3.45 | 0.023 | <0.01 |
| Tropomyosin 1 (alpha) | Z24727 | 210986_s_at | 756.33 | 2438.52 | 3.22 | 0.0046 | <0.01 |
| Insulin-like growth factor binding protein 3 | M31159 | 210095_s_at | 986.38 | 3159.58 | 3.20 | 0.0030 | <0.01 |
| Phosphofructokinase, platelet | NM_002627 | 201037_at | 175.40 | 529.33 | 3.02 | 0.00033 | <0.01 |
| Chromosome 5 open reading frame 13 | NM_004772 | 201310_s_at | 430.04 | 1233.67 | 2.87 | 0.0053 | <0.01 |
| Chromosome 5 open reading frame 13 | U36189 | 201309_x_at | 193.25 | 552.20 | 2.86 | 0.0032 | <0.01 |
| Collagen, type V, alpha 1 | AI983428 | 212489_at | 219.71 | 627.74 | 2.86 | 0.015 | <0.01 |
| Collagen, type V, alpha 1 | N30339 | 212488_at | 528.69 | 1455.41 | 2.75 | 0.016 | <0.01 |
| Versican | D32039 | 211571_s_at | 164.13 | 441.26 | 2.69 | 0.00068 | <0.01 |
| Versican | NM_004385 | 204620_s_at | 353.15 | 891.88 | 2.53 | 0.0015 | <0.01 |
| Myeloid/lymphoid or mixed-lineage leukemia; translocated to, 11 | BC006471 | 211071_s_at | 90.40 | 223.09 | 2.47 | 0.000011 | <0.01 |
| Versican | BF218922 | 221731_x_at | 351.23 | 846.90 | 2.41 | 0.00059 | <0.01 |
| **Annotation** | **Accession** | **Probe Set ID** | **Control mean** | **SSc-ILD mean** | **Fold change** | **p value** | **q-value(%)** |
| Tubulin, beta 3 | AL565749 | 213476_x_at | 420.77 | 952.29 | 2.26 | 0.0023 | <0.01 |
| Tubulin, beta 3 | NM_006086 | 202154_x_at | 449.19 | 968.49 | 2.16 | 0.0083 | <0.01 |
| POTE ankyrin domain family, member K, pseudogene | AY014272 | 210926_at | 272.13 | 578.80 | 2.13 | 0.0043 | <0.01 |
| Proteasome 26S subunit, non-ATPase, 2 | NM_002808 | 200830_at | 322.02 | 680.55 | 2.11 | 0.0033 | <0.01 |
| cAMP responsive element binding protein 3-like 2 | BE675139 | 212345_s_at | 290.05 | 612.65 | 2.11 | 0.011 | <0.01 |
| Tubulin, beta 6 | BC002654 | 209191_at | 588.66 | 1204.43 | 2.05 | 0.0075 | <0.01 |
| CTP synthase | NM_001905 | 202613_at | 39.48 | 245.10 | 6.21 | 0.0061 | 0.012 |
| Leucine rich repeat containing 15 | AU147799 | 213909_at | 81.45 | 363.87 | 4.47 | 0.010 | 0.012 |
| Prostaglandin-endoperoxide synthase 1 (prostaglandin G/H synthase and cyclooxygenase) | NM_000962 | 205128_x_at | 122.75 | 405.05 | 3.30 | 0.010 | 0.012 |
| Versican | R94644 | 215646_s_at | 141.85 | 353.27 | 2.49 | 0.0020 | 0.012 |
| TCDD-inducible poly(ADP-ribose) polymerase | AL556438 | 212665_at | 147.41 | 326.67 | 2.22 | 0.00071 | 0.012 |
| Tropomyosin 1 (alpha) | NM_000366 | 206117_at | 30.90 | 186.96 | 6.05 | 0.0033 | 0.022 |
| Tumor necrosis factor receptor superfamily, member 12A | NM_016639 | 218368_s_at | 175.59 | 440.75 | 2.51 | 0.016 | 0.022 |
| NADPH oxidase 4 | NM_016931 | 219773_at | 12.26 | 206.59 | 16.86 | 0.012 | 0.030 |
| Proenkephalin | NM_006211 | 213791_at | 33.71 | 314.31 | 9.32 | 0.029 | 0.030 |
| Dapper, antagonist of beta-catenin, homolog 1 (Xenopus laevis) | NM_016651 | 219179_at | 33.34 | 173.36 | 5.20 | 0.0027 | 0.030 |
| Topoisomerase (DNA) II alpha 170kDa | AL561834 | 201292_at | 44.15 | 190.98 | 4.33 | 0.0037 | 0.030 |
| Growth arrest and DNA-damage-inducible, beta | AF078077 | 209305_s_at | 47.37 | 182.72 | 3.86 | 0.0034 | 0.030 |
| PRKC, apoptosis, WT1, regulator | AI336206 | 204004_at | 69.24 | 238.13 | 3.44 | 0.0086 | 0.030 |
| Ribonucleotide reductase M2 | BC001886 | 209773_s_at | 103.33 | 353.46 | 3.42 | 0.021 | 0.030 |
| Ubiquitin-conjugating enzyme E2C | NM_007019 | 202954_at | 92.01 | 291.33 | 3.17 | 0.014 | 0.030 |
| UDP-N-acetyl-alpha-D-galactosamine | BE906572 | 212256_at | 54.51 | 171.76 | 3.15 | 0.0018 | 0.030 |
| Procollagen-lysine, 2-oxoglutarate 5-dioxygenase 2 | AI754404 | 202619_s_at | 200.84 | 629.11 | 3.13 | 0.036 | 0.030 |
| Glutaminase | NM_014905 | 203159_at | 70.13 | 209.34 | 2.98 | 0.0031 | 0.030 |
| 5'-nucleotidase domain containing 2 | NM_022908 | 218051_s_at | 51.71 | 152.69 | 2.95 | 0.00028 | 0.030 |
| ADAM metallopeptidase domain 12 | NM_003474 | 202952_s_at | 128.25 | 376.64 | 2.94 | 0.013 | 0.030 |
| Uridine-cytidine kinase 2 | BC002906 | 209825_s_at | 79.65 | 229.59 | 2.88 | 0.0049 | 0.030 |
| ATPase, Na+/K+ transporting, beta 1 polypeptide | BC000006 | 201242_s_at | 77.89 | 215.08 | 2.76 | 0.00066 | 0.030 |
| Histone cluster 1, H4c | NM_003542 | 205967_at | 121.16 | 333.46 | 2.75 | 0.013 | 0.030 |
| Cysteine and glycine-rich protein 2 | U46006 | 211126_s_at | 124.93 | 341.87 | 2.74 | 0.012 | 0.030 |
| Ornithine decarboxylase 1 | NM_002539 | 200790_at | 105.82 | 280.04 | 2.65 | 0.0072 | 0.030 |
| Tubulin, beta 2A | BF971587 | 209372_x_at | 72.01 | 188.83 | 2.62 | 0.0022 | 0.030 |
| Hematological and neurological expressed 1 | NM_016185 | 217755_at | 91.20 | 229.50 | 2.52 | 0.0036 | 0.030 |
| ATP citrate lyase | U18197 | 210337_s_at | 171.95 | 360.98 | 2.10 | 0.0019 | 0.030 |
| Transgelin | NM_003186 | 205547_s_at | 1004.01 | 2054.46 | 2.05 | 0.026 | 0.030 |
| Spermidine synthase | NM_003132 | 201516_at | 116.04 | 236.03 | 2.03 | 0.00034 | 0.030 |
| Follistatin-like 3 (secreted glycoprotein) | NM_005860 | 203592_s_at | 32.53 | 168.45 | 5.18 | 0.0079 | 0.048 |
| NUAK family, SNF1-like kinase, 1 | NM_014840 | 204589_at | 56.16 | 232.84 | 4.15 | 0.016 | 0.048 |
| Cyclin-dependent kinase 1 | D88357 | 210559_s_at | 52.88 | 204.47 | 3.87 | 0.0094 | 0.048 |
| **Annotation** | **Accession** | **Probe Set ID** | **Control mean** | **SSc-ILD mean** | **Fold change** | **p value** | **q-value(%)** |
| ADAM metallopeptidase domain 19 | Y13786 | 209765_at | 60.22 | 194.06 | 3.22 | 0.0046 | 0.048 |
| Procollagen-lysine, 2-oxoglutarate 5-dioxygenase 2 | NM_000935 | 202620_s_at | 144.10 | 414.59 | 2.88 | 0.038 | 0.048 |
| Ectonucleotide pyrophosphatase/phosphodiesterase 1 | NM_006208 | 205066_s_at | 109.70 | 296.12 | 2.70 | 0.013 | 0.048 |
| KIAA0101 | NM_014736 | 202503_s_at | 133.51 | 326.43 | 2.44 | 0.017 | 0.048 |
| Collagen, type IV, alpha 1 | AI922605 | 211980_at | 365.60 | 870.69 | 2.38 | 0.048 | 0.048 |
| H2A histone family, member X | NM_002105 | 205436_s_at | 161.94 | 377.68 | 2.33 | 0.017 | 0.048 |
| Plasminogen activator, urokinase receptor | U08839 | 210845_s_at | 120.27 | 268.46 | 2.23 | 0.0083 | 0.048 |
| Solute carrier family 7, member 1 | AA148507 | 212295_s_at | 171.25 | 352.68 | 2.06 | 0.014 | 0.048 |
| Cytokine receptor-like factor 1 | NM_004750 | 206315_at | 23.43 | 128.54 | 5.49 | 0.0042 | 0.054 |
| Cyclin A2 | NM_001237 | 203418_at | 26.97 | 128.99 | 4.78 | 0.0044 | 0.054 |
| Cyclin B1 | BE407516 | 214710_s_at | 42.74 | 180.59 | 4.23 | 0.032 | 0.054 |
| Cyclin-dependent kinase 1 | AL524035 | 203213_at | 42.19 | 171.61 | 4.07 | 0.011 | 0.054 |
| Protein regulator of cytokinesis 1 | NM_003981 | 218009_s_at | 41.41 | 165.05 | 3.99 | 0.015 | 0.054 |
| Topoisomerase (DNA) II alpha 170kDa | AU159942 | 201291_s_at | 55.26 | 219.87 | 3.98 | 0.018 | 0.054 |
| Syndecan 1 | Z48199 | 201286_at | 36.50 | 144.36 | 3.95 | 0.021 | 0.054 |
| TPX2, microtubule-associated, homolog (Xenopus laevis) | AF098158 | 210052_s_at | 41.86 | 163.78 | 3.91 | 0.018 | 0.054 |
| Cyclin-dependent kinase 1 | NM_001786 | 203214_x_at | 46.80 | 177.95 | 3.80 | 0.012 | 0.054 |
| Fatty acid binding protein 5 | NM_001444 | 202345_s_at | 47.30 | 178.07 | 3.76 | 0.033 | 0.054 |
| Cadherin 2, type 1, N-cadherin (neuronal) | NM_001792 | 203441_s_at | 45.15 | 163.70 | 3.63 | 0.0099 | 0.054 |
| Leukemia inhibitory factor (cholinergic differentiation factor) | NM_002309 | 205266_at | 46.52 | 166.79 | 3.59 | 0.0053 | 0.054 |
| Ribonucleotide reductase M2 | BE966236 | 201890_at | 56.12 | 197.08 | 3.51 | 0.020 | 0.054 |
| ZW10 interactor | NM_007057 | 204026_s_at | 53.65 | 187.41 | 3.49 | 0.012 | 0.054 |
| Collagen, type VII, alpha 1 | NM_000094 | 204136_at | 81.29 | 279.94 | 3.44 | 0.029 | 0.054 |
| Centromere protein F, 350/400kDa (mitosin) | NM_005196 | 207828_s_at | 61.18 | 207.92 | 3.40 | 0.013 | 0.054 |
| Cyclin B2 | NM_004701 | 202705_at | 49.44 | 165.79 | 3.35 | 0.013 | 0.054 |
| PDZ and LIM domain 5 | NM_006457 | 203243_s_at | 53.22 | 175.79 | 3.30 | 0.0066 | 0.054 |
| Smoothelin | NM_006932 | 207390_s_at | 46.47 | 147.71 | 3.18 | 0.0047 | 0.054 |
| Smoothelin | AF064238 | 209427_at | 51.64 | 154.70 | 3.00 | 0.0056 | 0.054 |
| Transmembrane protein 2 | NM_013390 | 218113_at | 94.73 | 283.11 | 2.99 | 0.046 | 0.054 |
| RAB3B, member RAS oncogene family | BC005035 | 205924_at | 76.96 | 205.42 | 2.67 | 0.011 | 0.054 |
| Maternal embryonic leucine zipper kinase | NM_014791 | 204825_at | 74.13 | 194.32 | 2.62 | 0.023 | 0.054 |
| Pituitary tumor-transforming 1 | NM_004219 | 203554_x_at | 149.22 | 361.15 | 2.42 | 0.040 | 0.054 |
| Isocitrate dehydrogenase 2 (NADP+), mitochondrial | U52144 | 210046_s_at | 117.64 | 269.41 | 2.29 | 0.019 | 0.054 |
| Fermitin family member 2 | AW469573 | 209209_s_at | 117.61 | 265.36 | 2.26 | 0.016 | 0.054 |
| Phosphoglucomutase 3 | BC001258 | 210041_s_at | 109.99 | 242.72 | 2.21 | 0.0089 | 0.054 |
| Eukaryotic translation initiation factor 4E binding protein 1 | AB044548 | 221539_at | 104.34 | 230.53 | 2.21 | 0.010 | 0.054 |
| Versican | BF590263 | 204619_s_at | 128.19 | 273.34 | 2.13 | 0.019 | 0.054 |
| Ribonucleotide reductase M1 | NM_001033 | 201477_s_at | 102.65 | 216.19 | 2.11 | 0.015 | 0.054 |
| Cell division cycle 20 homolog (S. cerevisiae) | NM_001255 | 202870_s_at | 20.69 | 143.24 | 6.92 | 0.042 | 0.069 |
| Collagen, type XI, alpha 1 | NM_001854 | 204320_at | 31.27 | 145.79 | 4.66 | 0.024 | 0.069 |
| **Annotation** | **Accession** | **Probe Set ID** | **Control mean** | **SSc-ILD mean** | **Fold change** | **p value** | **q-value(%)** |
| PDZ binding kinase | NM_018492 | 219148_at | 36.85 | 147.55 | 4.00 | 0.022 | 0.069 |
| Nucleolar and spindle associated protein 1 | NM_016359 | 218039_at | 67.38 | 182.22 | 2.70 | 0.018 | 0.069 |
| Met proto-oncogene (hepatocyte growth factor receptor) | BG170541 | 203510_at | 72.74 | 184.74 | 2.54 | 0.034 | 0.069 |
| SET and MYND domain containing 3 | NM_022743 | 218788_s_at | 74.68 | 179.78 | 2.41 | 0.019 | 0.069 |
| Protein phosphatase 1, regulatory (inhibitor) subunit 14B | BE305165 | 212680_x_at | 103.57 | 229.14 | 2.21 | 0.035 | 0.069 |
| Forkhead box D1 | NM_004472 | 206307_s_at | 132.29 | 274.90 | 2.08 | 0.031 | 0.069 |
| Ribonucleotide reductase M1 | AI692974 | 201476_s_at | 103.53 | 214.12 | 2.07 | 0.023 | 0.069 |
| **Underexpressed in SSc-ILD** |  |  |  |  |  |  |  |
| Chemokine (C-X-C motif) ligand 10 | NM_001565 | 204533_at | 771.16 | 19.22 | -40.12 | 0.00034 | <0.01 |
| Chemokine (C-X-C motif) ligand 11 | AF030514 | 210163_at | 179.86 | 4.99 | -36.04 | 0.0027 | <0.01 |
| Flavin containing monooxygenase 2 (non-functional) | BC005894 | 211726_s_at | 530.43 | 15.73 | -33.73 | 0.017 | <0.01 |
| Interferon-induced protein with tetratricopeptide repeats 2 | BE888744 | 217502_at | 707.12 | 26.10 | -27.09 | 0.0096 | <0.01 |
| Vascular cell adhesion molecule 1 | NM_001078 | 203868_s_at | 835.20 | 32.17 | -25.96 | 0.0049 | <0.01 |
| Bone marrow stromal cell antigen 2 | NM_004335 | 201641_at | 315.76 | 12.49 | -25.27 | 0.0081 | <0.01 |
| Radical S-adenosyl methionine domain containing 2 | AI337069 | 213797_at | 333.76 | 13.28 | -25.14 | 0.0039 | <0.01 |
| Interferon-induced protein 44-like | NM_006820 | 204439_at | 370.65 | 15.36 | -24.13 | 0.00033 | <0.01 |
| Interferon-induced protein with tetratricopeptide repeats 1 | NM_001548 | 203153_at | 1744.16 | 82.58 | -21.12 | 0.000039 | <0.01 |
| 2',5'-oligoadenylate synthetase 1, 40/46kDa | NM_002534 | 205552_s_at | 374.61 | 18.32 | -20.45 | 0.0014 | <0.01 |
| Complement factor B | NM_001710 | 202357_s_at | 837.04 | 42.64 | -19.63 | 0.0036 | <0.01 |
| Chemokine (C-X-C motif) ligand 11 | AF002985 | 211122_s_at | 165.76 | 8.57 | -19.34 | 0.00058 | <0.01 |
| 2',5'-oligoadenylate synthetase 1, 40/46kDa | NM_016816 | 202869_at | 482.74 | 26.56 | -18.18 | 0.0018 | <0.01 |
| Interferon-induced protein with tetratricopeptide repeats 3 | NM_001549 | 204747_at | 985.04 | 61.94 | -15.90 | 0.00031 | <0.01 |
| Chromosome 10 open reading frame 10 | AL136653 | 209183_s_at | 208.79 | 13.64 | -15.31 | 0.0062 | <0.01 |
| Myxovirus resistance 1, interferon-inducible protein p78 (mouse) | NM_002462 | 202086_at | 1361.89 | 91.23 | -14.93 | 0.00012 | <0.01 |
| Receptor (chemosensory) transporter protein 4 | NM_022147 | 219684_at | 196.44 | 13.26 | -14.82 | 0.000091 | <0.01 |
| Chemokine (C-C motif) ligand 11 | D49372 | 210133_at | 529.84 | 36.18 | -14.64 | 0.0021 | <0.01 |
| Retinoic acid receptor responder (tazarotene induced) 3 | NM_004585 | 204070_at | 239.43 | 17.24 | -13.89 | 0.00024 | <0.01 |
| Alcohol dehydrogenase 1B (class I), beta polypeptide | M21692 | 209613_s_at | 268.94 | 19.82 | -13.57 | 0.011 | <0.01 |
| Secreted and transmembrane 1 | BF939675 | 213716_s_at | 285.00 | 21.99 | -12.96 | 0.0012 | <0.01 |
| Interferon, alpha-inducible protein 6 | NM_022873 | 204415_at | 1196.56 | 93.67 | -12.77 | 0.00043 | <0.01 |
| Myxovirus resistance 2 (mouse) | NM_002463 | 204994_at | 517.28 | 40.63 | -12.73 | 0.00089 | <0.01 |
| Interferon induced with helicase C domain 1 | NM_022168 | 219209_at | 354.78 | 29.67 | -11.96 | 0.0028 | <0.01 |
| Interferon, alpha-inducible protein 27 | NM_005532 | 202411_at | 897.46 | 78.80 | -11.39 | 0.0026 | <0.01 |
| 2'-5'-oligoadenylate synthetase-like | NM_003733 | 205660_at | 246.36 | 21.63 | -11.39 | 0.019 | <0.01 |
| Solute carrier family 39 (zinc transporter), member 8 | AB040120 | 209267_s_at | 494.00 | 43.88 | -11.26 | 0.0031 | <0.01 |
| Alcohol dehydrogenase 1B (class I), beta polypeptide | M24317 | 209612_s_at | 606.08 | 54.45 | -11.13 | 0.012 | <0.01 |
| Hect domain and RLD 6 | NM_017912 | 219352_at | 274.04 | 25.73 | -10.65 | 0.000007 | <0.01 |
| Retinoic acid receptor responder (tazarotene induced) 2 | BC000069 | 209496_at | 450.34 | 43.85 | -10.27 | 0.010 | <0.01 |
| 2'-5'-oligoadenylate synthetase 2, 69/71kDa | NM_016817 | 204972_at | 279.23 | 27.91 | -10.00 | 0.00038 | <0.01 |
| Interferon regulatory factor 7 | NM_004030 | 208436_s_at | 390.28 | 39.53 | -9.87 | 0.00082 | <0.01 |
| **Annotation** | **Accession** | **Probe Set ID** | **Control mean** | **SSc-ILD mean** | **Fold change** | **p value** | **q-value(%)** |
| Chemokine (C-C motif) ligand 7 | NM_006273 | 208075_s_at | 215.28 | 23.08 | -9.33 | 0.000096 | <0.01 |
| Interferon-induced protein 35 | BC001356 | 209417_s_at | 455.61 | 49.12 | -9.28 | 0.000082 | <0.01 |
| Apolipoprotein L, 3 | NM_014349 | 221087_s_at | 124.85 | 13.52 | -9.23 | 0.00024 | <0.01 |
|  |  |  |  |  |  |  |  |
| ISG15 ubiquitin-like modifier | NM_005101 | 205483_s_at | 3787.75 | 411.64 | -9.20 | <0.000001 | <0.01 |
| Selenoprotein P, plasma, 1 | NM_005410 | 201427_s_at | 345.19 | 37.87 | -9.12 | 0.012 | <0.01 |
| Apolipoprotein L, 1 | AF323540 | 209546_s_at | 139.58 | 15.50 | -9.00 | 0.00024 | <0.01 |
| Interleukin 15 receptor, alpha | NM_002189 | 207375_s_at | 170.29 | 19.41 | -8.77 | 0.00057 | <0.01 |
| Solute carrier family 39 (zinc transporter), member 8 | NM_022154 | 219869_s_at | 228.81 | 26.27 | -8.71 | 0.0054 | <0.01 |
| Interferon induced transmembrane protein 1 (9-27) | AA749101 | 214022_s_at | 3698.03 | 443.31 | -8.34 | <0.000001 | <0.01 |
| Interferon-induced protein 44 | NM_006417 | 214453_s_at | 335.01 | 41.06 | -8.16 | 0.000038 | <0.01 |
| DEAD (Asp-Glu-Ala-Asp) box polypeptide 58 | NM_014314 | 218943_s_at | 391.58 | 49.78 | -7.87 | 0.0020 | <0.01 |
| Transmembrane protein 140 | NM_018295 | 218999_at | 156.91 | 20.11 | -7.80 | 0.00006 | <0.01 |
| Proteasome subunit, beta type, 9 | NM_002800 | 204279_at | 395.20 | 51.45 | -7.68 | 0.0016 | <0.01 |
| Signal transducer and activator of transcription 1, 91kDa | BC002704 | 209969_s_at | 327.32 | 44.57 | -7.34 | 0.00017 | <0.01 |
| Interferon, gamma-inducible protein 30 | NM_006332 | 201422_at | 199.42 | 27.67 | -7.21 | 0.0061 | <0.01 |
| Superoxide dismutase 2, mitochondrial | BF575213 | 221477_s_at | 2180.37 | 311.04 | -7.01 | <0.000001 | <0.01 |
| Pentraxin 3, long | NM_002852 | 206157_at | 1415.20 | 202.98 | -6.97 | 0.000019 | <0.01 |
| Chemokine (C-C motif) ligand 2 | S69738 | 216598_s_at | 2676.36 | 385.53 | -6.94 | 0.000055 | <0.01 |
| 2'-5'-oligoadenylate synthetase 2, 69/71kDa | NM_002535 | 206553_at | 117.22 | 17.12 | -6.85 | 0.00087 | <0.01 |
| Superoxide dismutase 2, mitochondrial | W46388 | 215223_s_at | 1587.48 | 236.02 | -6.73 | <0.000001 | <0.01 |
| Tumor necrosis factor, alpha-induced protein 2 | NM_006291 | 202510_s_at | 404.24 | 63.56 | -6.36 | 0.000003 | <0.01 |
| Phospholipid scramblase 1 | NM_021105 | 202430_s_at | 470.63 | 74.34 | -6.33 | 0.00003 | <0.01 |
| Cathepsin S | NM_004079 | 202902_s_at | 204.78 | 34.18 | -5.99 | 0.0031 | <0.01 |
| Tripartite motif-containing 22 | AA083478 | 213293_s_at | 1052.31 | 178.30 | -5.90 | 0.000037 | <0.01 |
| Superoxide dismutase 2, mitochondrial | X15132 | 216841_s_at | 484.78 | 84.08 | -5.77 | 0.000018 | <0.01 |
| Chemokine (C-X-C motif) ligand 2 | M57731 | 209774_x_at | 144.94 | 25.11 | -5.77 | 0.000073 | <0.01 |
| DEAD (Asp-Glu-Ala-Asp) box polypeptide 60 | NM_017631 | 218986_s_at | 253.21 | 44.30 | -5.72 | 0.000028 | <0.01 |
| 2'-5'-oligoadenylate synthetase 3, 100kDa | NM_006187 | 218400_at | 305.50 | 53.43 | -5.72 | 0.00015 | <0.01 |
| Ubiquitin-conjugating enzyme E2L 6 | NM_004223 | 201649_at | 770.87 | 136.41 | -5.65 | 0.000012 | <0.01 |
| Phospholipid scramblase 1 | AI825926 | 202446_s_at | 746.01 | 134.49 | -5.55 | 0.000036 | <0.01 |
| Regulator of calcineurin 2 | NM_005822 | 203498_at | 142.46 | 25.74 | -5.53 | 0.00039 | <0.01 |
| KIAA1199 | AB033025 | 212942_s_at | 799.03 | 144.95 | -5.51 | 0.00071 | <0.01 |
| Poly (ADP-ribose) polymerase family, member 12 | NM_022750 | 218543_s_at | 213.75 | 39.08 | -5.47 | 0.000033 | <0.01 |
| Hydroxysteroid (11-beta) dehydrogenase 1 | NM_005525 | 205404_at | 430.46 | 78.97 | -5.45 | 0.0093 | <0.01 |
| Lymphocyte antigen 6 complex, locus E | NM_002346 | 202145_at | 966.94 | 177.81 | -5.44 | 0.000045 | <0.01 |
| Sushi, von Willebrand factor type A, EGF and pentraxin domain containing 1 | AA716107 | 213247_at | 332.92 | 61.79 | -5.39 | 0.0019 | <0.01 |
| XIAP associated factor 1 | NM_017523 | 206133_at | 190.99 | 35.58 | -5.37 | 0.00044 | <0.01 |
| Hepatocyte growth factor (hepapoietin A) | X16323 | 209960_at | 215.20 | 40.31 | -5.34 | 0.014 | <0.01 |
| Transient receptor potential cation channel, subfamily A, member 1 | AA502609 | 217590_s_at | 588.36 | 111.31 | -5.29 | 0.0095 | <0.01 |
| Caspase 1 (interleukin 1, beta, convertase) | U13700 | 211368_s_at | 154.32 | 29.61 | -5.21 | 0.000002 | <0.01 |
| **Annotation** | **Accession** | **Probe Set ID** | **Control mean** | **SSc-ILD mean** | **Fold change** | **p value** | **q-value(%)** |
| Leucine aminopeptidase 3 | NM_015907 | 217933_s_at | 705.29 | 136.52 | -5.17 | 0.0026 | <0.01 |
| Caspase 1 (interleukin 1, beta, convertase) | U13699 | 211367_s_at | 180.59 | 35.05 | -5.15 | <0.000001 | <0.01 |
| Chemokine (C-X-C motif) ligand 1 | NM_001511 | 204470_at | 637.05 | 123.92 | -5.14 | <0.000001 | <0.01 |
| Three prime repair exonuclease 1 | NM_016381 | 205875_s_at | 127.55 | 24.89 | -5.13 | 0.00059 | <0.01 |
| Ubiquitin specific peptidase 18 | NM_017414 | 219211_at | 134.72 | 26.59 | -5.07 | 0.0013 | <0.01 |
| Tripartite motif-containing 14 | NM_014788 | 203148_s_at | 160.01 | 32.32 | -4.95 | 0.000001 | <0.01 |
| Guanylate binding protein 2, interferon-inducible | NM_004120 | 202748_at | 216.57 | 44.17 | -4.90 | 0.00036 | <0.01 |
| Interleukin 6 signal transducer (gp130) | AB015706 | 211000_s_at | 529.90 | 108.08 | -4.90 | 0.0043 | <0.01 |
| SP110 nuclear body protein | NM_004509 | 208012_x_at | 228.58 | 46.71 | -4.89 | 0.00042 | <0.01 |
| Serpin peptidase inhibitor, clade G (C1 inhibitor), member 1 | NM_000062 | 200986_at | 416.16 | 86.54 | -4.81 | 0.00098 | <0.01 |
| Chemokine (C-X-C motif) ligand 6 | NM_002993 | 206336_at | 425.78 | 90.06 | -4.73 | 0.0019 | <0.01 |
| Phosphatidic acid phosphatase type 2B | AB000889 | 209355_s_at | 1566.08 | 332.45 | -4.71 | 0.000003 | <0.01 |
| C-type lectin domain family 2, member B | BC005254 | 209732_at | 595.30 | 126.45 | -4.71 | 0.022 | <0.01 |
| Growth arrest-specific 1 | NM_002048 | 204457_s_at | 383.22 | 81.59 | -4.70 | 0.00019 | <0.01 |
| Adrenomedullin | NM_001124 | 202912_at | 1968.38 | 421.93 | -4.67 | 0.000011 | <0.01 |
| Phosphatidic acid phosphatase type 2B | AA628586 | 212226_s_at | 2495.93 | 536.86 | -4.65 | 0.000011 | <0.01 |
| Hect domain and RLD 5 | NM_016323 | 219863_at | 340.15 | 73.86 | -4.61 | 0.00074 | <0.01 |
| Interferon induced transmembrane protein 1 (9-27) | NM_003641 | 201601_x_at | 4134.68 | 897.96 | -4.60 | <0.000001 | <0.01 |
| Olfactomedin-like 1 | AW305097 | 217525_at | 149.66 | 32.98 | -4.54 | 0.0011 | <0.01 |
| Phosphatidic acid phosphatase type 2B | AV725664 | 212230_at | 1344.37 | 296.77 | -4.53 | 0.000007 | <0.01 |
| Tumor necrosis factor receptor superfamily, member 11b | NM_002546 | 204933_s_at | 1231.11 | 277.60 | -4.43 | 0.0015 | <0.01 |
| SP110 nuclear body protein | AA969194 | 209761_s_at | 171.10 | 38.84 | -4.40 | 0.00061 | <0.01 |
| Interleukin 6 signal transducer (gp130) | BE856546 | 204863_s_at | 786.04 | 179.76 | -4.37 | 0.0010 | <0.01 |
| Interleukin 6 signal transducer (gp130) | AW242916 | 212196_at | 813.69 | 186.59 | -4.36 | 0.0039 | <0.01 |
| Major histocompatibility complex, class I, E | X56841 | 200904_at | 537.54 | 125.18 | -4.29 | 0.000002 | <0.01 |
| Stomatin | M81635 | 201061_s_at | 851.55 | 198.97 | -4.28 | 0.00008 | <0.01 |
| Complement component 3 | NM_000064 | 217767_at | 457.89 | 107.33 | -4.27 | 0.00031 | <0.01 |
| N-myc (and STAT) interactor | NM_004688 | 203964_at | 307.52 | 72.41 | -4.25 | 0.000068 | <0.01 |
| DNA-damage regulated autophagy modulator 1 | NM_018370 | 218627_at | 1126.08 | 269.16 | -4.18 | 0.000001 | <0.01 |
| Complement component 1, r subcomponent | AL573058 | 212067_s_at | 1448.63 | 350.18 | -4.14 | 0.000064 | <0.01 |
| Quiescin Q6 sulfhydryl oxidase 1 | NM_002826 | 201482_at | 1457.49 | 353.25 | -4.13 | 0.016 | <0.01 |
| Caspase 1 (interleukin 1, beta, convertase) | M87507 | 209970_x_at | 331.85 | 80.61 | -4.12 | 0.000001 | <0.01 |
| Caspase 1 (interleukin 1, beta, convertase) | U13698 | 211366_x_at | 364.20 | 88.39 | -4.12 | <0.000001 | <0.01 |
| Butyrophilin, subfamily 3, member A2 | NM_006994 | 204820_s_at | 371.47 | 91.52 | -4.06 | 0.00063 | <0.01 |
| Platelet derived growth factor D | NM_025208 | 219304_s_at | 173.66 | 43.11 | -4.03 | 0.0016 | <0.01 |
| Tumor necrosis factor receptor superfamily, member 11b | BF433902 | 204932_at | 233.73 | 58.34 | -4.01 | 0.0011 | <0.01 |
| Tumor necrosis factor, alpha-induced protein 3 | NM_006290 | 202644_s_at | 237.81 | 59.81 | -3.98 | 0.000016 | <0.01 |
| Odd-skipped related 2 (Drosophila) | AI811298 | 213568_at | 144.37 | 36.28 | -3.98 | 0.00043 | <0.01 |
| SP110 nuclear body protein | AF280094 | 209762_x_at | 171.04 | 43.33 | -3.95 | 0.00019 | <0.01 |
| Mitogen-activated protein kinase kinase kinase 5 | NM_005923 | 203837_at | 174.28 | 44.40 | -3.93 | 0.0017 | <0.01 |
| **Annotation** | **Accession** | **Probe Set ID** | **Control mean** | **SSc-ILD mean** | **Fold change** | **p value** | **q-value(%)** |
| Colony stimulating factor 1 (macrophage) | M37435 | 209716_at | 247.62 | 63.15 | -3.92 | 0.000006 | <0.01 |
| Mitogen-activated protein kinase kinase kinase 5 | D84476 | 203836_s_at | 158.06 | 40.42 | -3.91 | 0.00011 | <0.01 |
| Tumor necrosis factor, alpha-induced protein 3 | AI738896 | 202643_s_at | 148.03 | 38.01 | -3.89 | 0.0010 | <0.01 |
| Adenosine monophosphate deaminase 3 | NM_000480 | 207992_s_at | 138.40 | 35.87 | -3.86 | 0.00022 | <0.01 |
| Interferon, gamma-inducible protein 16 | BG256677 | 208965_s_at | 499.74 | 130.48 | -3.83 | 0.00020 | <0.01 |
| Wilms tumor 1 associated protein | NM_004906 | 203137_at | 718.00 | 188.21 | -3.81 | 0.000007 | <0.01 |
| Stomatin | AI537887 | 201060_x_at | 513.40 | 134.67 | -3.81 | 0.000024 | <0.01 |
| Major histocompatibility complex, class I, G | AF226990 | 210514_x_at | 1406.95 | 368.96 | -3.81 | 0.00012 | <0.01 |
| Histone cluster 1, H2ac | AL353759 | 215071_s_at | 321.95 | 84.55 | -3.81 | 0.0043 | <0.01 |
| SMAD family member 3 | NM_015400 | 218284_at | 263.84 | 69.27 | -3.81 | 0.0058 | <0.01 |
| Interferon, gamma-inducible protein 16 | AF208043 | 208966_x_at | 957.31 | 253.28 | -3.78 | 0.000007 | <0.01 |
| Interferon, gamma-inducible protein 16 | NM_005531 | 206332_s_at | 764.46 | 203.44 | -3.76 | 0.000021 | <0.01 |
| Bradykinin receptor B2 | NM_000623 | 205870_at | 407.71 | 109.24 | -3.73 | 0.000001 | <0.01 |
| Methyltransferase like 7A | NM_014033 | 207761_s_at | 219.19 | 59.15 | -3.71 | 0.0018 | <0.01 |
| Chromosome 19 open reading frame 66 | NM_018381 | 218429_s_at | 149.14 | 40.52 | -3.68 | 0.00032 | <0.01 |
| Complement factor H | X56210 | 215388_s_at | 437.48 | 118.72 | -3.68 | 0.018 | <0.01 |
| Major histocompatibility complex, class I, G | M90684 | 211529_x_at | 1596.04 | 434.44 | -3.67 | 0.00012 | <0.01 |
| Cytochrome b5 type A (microsomal) | M22865 | 209366_x_at | 478.26 | 130.55 | -3.66 | 0.000017 | <0.01 |
| Aldo-keto reductase family 1, member C2 | U05598 | 209699_x_at | 1413.06 | 387.06 | -3.65 | 0.000095 | <0.01 |
| Eukaryotic translation initiation factor 2-alpha kinase 2 | NM_002759 | 204211_x_at | 213.57 | 58.72 | -3.64 | 0.000032 | <0.01 |
| Serglycin | J03223 | 201858_s_at | 550.00 | 151.08 | -3.64 | 0.018 | <0.01 |
| Transporter 1, ATP-binding cassette, sub-family B | NM_000593 | 202307_s_at | 326.14 | 89.73 | -3.63 | 0.00079 | <0.01 |
| Tissue factor pathway inhibitor | BF511231 | 213258_at | 421.90 | 116.18 | -3.63 | 0.010 | <0.01 |
| Signal transducer and activator of transcription 1, 91kDa | NM_007315 | 200887_s_at | 2028.99 | 561.13 | -3.62 | 0.000001 | <0.01 |
| Aldo-keto reductase family 1, member C1 | NM_001353 | 204151_x_at | 1649.18 | 457.13 | -3.61 | 0.000093 | <0.01 |
| Vesicle-associated membrane protein 5 | NM_006634 | 204929_s_at | 349.85 | 97.17 | -3.60 | 0.00016 | <0.01 |
| SP100 nuclear antigen | U36501 | 210218_s_at | 198.71 | 55.26 | -3.60 | 0.0068 | <0.01 |
| Complement component 1, s subcomponent | M18767 | 208747_s_at | 2721.67 | 757.25 | -3.59 | 0.000003 | <0.01 |
| Interferon regulatory factor 1 | NM_002198 | 202531_at | 198.74 | 55.50 | -3.58 | 0.000048 | <0.01 |
| Serglycin | NM_002727 | 201859_at | 816.74 | 228.32 | -3.58 | 0.019 | <0.01 |
| Wilms tumor 1 associated protein | BC000383 | 210285_x_at | 438.40 | 122.84 | -3.57 | 0.000002 | <0.01 |
| --- | AL121994 | 216565_x_at | 684.40 | 192.38 | -3.56 | 0.00003 | <0.01 |
| Major histocompatibility complex, class I, G | M90685 | 211528_x_at | 1715.65 | 482.96 | -3.55 | 0.00039 | <0.01 |
| TAP binding protein (tapasin) | AF029750 | 208829_at | 270.49 | 76.68 | -3.53 | 0.000003 | <0.01 |
| Major histocompatibility complex, class I, E | M31183 | 217456_x_at | 581.41 | 164.58 | -3.53 | 0.00012 | <0.01 |
| Potassium channel tetramerisation domain containing 12 | AI718937 | 212192_at | 246.53 | 71.10 | -3.47 | 0.011 | <0.01 |
| Interleukin 6 signal transducer (gp130) | NM_002184 | 204864_s_at | 144.41 | 41.77 | -3.46 | 0.0012 | <0.01 |
| Cytochrome b5 type A (microsomal) | NM_001914 | 207843_x_at | 415.89 | 120.97 | -3.44 | 0.000026 | <0.01 |
| Proteasome subunit, beta type, 8 | U17496 | 209040_s_at | 222.36 | 64.70 | -3.44 | 0.00013 | <0.01 |
| Major histocompatibility complex, class I, C | U62824 | 211799_x_at | 1112.53 | 323.28 | -3.44 | 0.00055 | <0.01 |
| **Annotation** | **Accession** | **Probe Set ID** | **Control mean** | **SSc-ILD mean** | **Fold change** | **p value** | **q-value(%)** |
| SMAD family member 3 | NM_005902 | 205398_s_at | 149.72 | 43.66 | -3.43 | 0.00092 | <0.01 |
| Cathepsin O | AV729484 | 203758_at | 231.63 | 68.18 | -3.40 | 0.000059 | <0.01 |
| Aldo-keto reductase family 1, member C1 | S68290 | 216594_x_at | 1136.23 | 335.13 | -3.39 | 0.00006 | <0.01 |
| Sulfide quinone reductase-like (yeast) | NM_021199 | 217995_at | 530.98 | 156.96 | -3.38 | 0.000001 | <0.01 |
| Bradykinin receptor B1 | NM_000710 | 207510_at | 524.20 | 155.00 | -3.38 | 0.0012 | <0.01 |
| Major histocompatibility complex, class I, F | AW514210 | 221875_x_at | 1272.83 | 378.69 | -3.36 | 0.00021 | <0.01 |
| Nuclear factor of kappa light polypeptide gene enhancer in B-cells inhibitor, alpha | AI078167 | 201502_s_at | 695.42 | 211.57 | -3.29 | 0.00001 | <0.01 |
| Tryptophanyl-tRNA synthetase | NM_004184 | 200629_at | 645.60 | 197.00 | -3.28 | 0.000041 | <0.01 |
| Lectin, galactoside-binding, soluble, 3 binding protein | NM_005567 | 200923_at | 506.23 | 154.97 | -3.27 | 0.00008 | <0.01 |
| Guanylate binding protein 1, interferon-inducible, 67kDa | BC002666 | 202269_x_at | 626.71 | 191.65 | -3.27 | 0.017 | <0.01 |
| Aldo-keto reductase family 1, member C2 | M33376 | 211653_x_at | 1100.18 | 338.61 | -3.25 | 0.00005 | <0.01 |
| Aldo-keto reductase family 1, member C3 | AB018580 | 209160_at | 247.34 | 76.30 | -3.24 | 0.00014 | <0.01 |
| Tumor necrosis factor, alpha-induced protein 6 | AW188198 | 206025_s_at | 1047.49 | 323.01 | -3.24 | 0.022 | <0.01 |
| Cytochrome b5 type A (microsomal) | M22976 | 215726_s_at | 251.43 | 77.91 | -3.23 | 0.00076 | <0.01 |
| Major histocompatibility complex, class I, E | NM_005516 | 200905_x_at | 1414.20 | 442.92 | -3.19 | 0.000025 | <0.01 |
| Dipeptidyl-peptidase 4 | NM_001935 | 203717_at | 396.67 | 125.03 | -3.17 | 0.0027 | <0.01 |
| Cathepsin K | NM_000396 | 202450_s_at | 2596.69 | 819.30 | -3.17 | 0.010 | <0.01 |
| Dipeptidyl-peptidase 4 | M80536 | 203716_s_at | 327.81 | 103.26 | -3.17 | 0.020 | <0.01 |
| Major histocompatibility complex, class I, C | AK024836 | 216526_x_at | 3043.48 | 964.14 | -3.16 | 0.00011 | <0.01 |
| Prostaglandin E synthase | AF010316 | 210367_s_at | 214.48 | 67.85 | -3.16 | 0.00068 | <0.01 |
| Prostaglandin I2 (prostacyclin) synthase | NM_000961 | 208131_s_at | 212.13 | 67.48 | -3.14 | 0.0036 | <0.01 |
| Eukaryotic translation initiation factor 2-alpha kinase 2 | AV755522 | 213294_at | 318.33 | 101.37 | -3.14 | <0.000001 | <0.01 |
| Interferon-induced protein with tetratricopeptide repeats 5 | N47725 | 203595_s_at | 187.98 | 60.03 | -3.13 | 0.000002 | <0.01 |
| Major histocompatibility complex, class I, B | L07950 | 211911_x_at | 2548.47 | 813.48 | -3.13 | 0.00035 | <0.01 |
| Glutaredoxin (thioltransferase) | AF162769 | 209276_s_at | 575.63 | 184.00 | -3.13 | 0.0027 | <0.01 |
| Major histocompatibility complex, class I, F | NM_018950 | 204806_x_at | 1239.65 | 396.69 | -3.12 | 0.000063 | <0.01 |
| Zinc finger protein 36, C3H type-like 2 | U07802 | 201368_at | 869.53 | 279.30 | -3.11 | 0.000001 | <0.01 |
| Thioredoxin interacting protein | AA812232 | 201008_s_at | 512.89 | 165.04 | -3.11 | 0.0063 | <0.01 |
| Rho family GTPase 3 | BG054844 | 212724_at | 2833.29 | 910.51 | -3.11 | <0.000001 | <0.01 |
| Pleiotrophin | AL565812 | 209465_x_at | 199.49 | 64.80 | -3.08 | 0.0015 | <0.01 |
| G protein-coupled receptor kinase 5 | NM_005308 | 204396_s_at | 222.36 | 72.52 | -3.07 | 0.0011 | <0.01 |
| Endothelial PAS domain protein 1 | AF052094 | 200878_at | 1875.53 | 612.98 | -3.06 | 0.0010 | <0.01 |
| Major histocompatibility complex, class I, A | M80469 | 217436_x_at | 773.14 | 253.30 | -3.05 | 0.00048 | <0.01 |
| Lumican | NM_002345 | 201744_s_at | 1252.91 | 411.48 | -3.04 | 0.000009 | <0.01 |
| Major histocompatibility complex, class I, C | M12679 | 214459_x_at | 2813.68 | 932.88 | -3.02 | 0.000061 | <0.01 |
| Peripheral myelin protein 22 | L03203 | 210139_s_at | 1142.20 | 378.29 | -3.02 | 0.000086 | <0.01 |
| Mannosidase, alpha, class 1A, member 1 | BG287153 | 221760_at | 236.85 | 78.42 | -3.02 | 0.00051 | <0.01 |
| Guanylate binding protein 1, interferon-inducible, 67kDa | NM_002053 | 202270_at | 271.06 | 90.06 | -3.01 | 0.0071 | <0.01 |
| Pleiotrophin | BC005916 | 211737_x_at | 676.08 | 225.25 | -3.00 | 0.0017 | <0.01 |
| PHD finger protein 11 | BF055474 | 221816_s_at | 379.52 | 127.00 | -2.99 | 0.000022 | <0.01 |
| **Annotation** | **Accession** | **Probe Set ID** | **Control mean** | **SSc-ILD mean** | **Fold change** | **p value** | **q-value(%)** |
| Kruppel-like factor 4 (gut) | BF514079 | 221841_s_at | 234.23 | 78.33 | -2.99 | 0.000036 | <0.01 |
| Glutaredoxin (thioltransferase) | NM_002064 | 206662_at | 793.08 | 265.44 | -2.99 | 0.0016 | <0.01 |
| Interleukin 1 receptor, type I | NM_000877 | 202948_at | 1273.37 | 429.19 | -2.97 | 0.000052 | <0.01 |
| Pleiotrophin | M57399 | 209466_x_at | 688.02 | 232.60 | -2.96 | 0.00066 | <0.01 |
| Major histocompatibility complex, class I, G | M90686 | 211530_x_at | 1322.04 | 447.38 | -2.96 | 0.0010 | <0.01 |
| Major histocompatibility complex, class I, B | D83043 | 208729_x_at | 2374.26 | 805.76 | -2.95 | 0.00028 | <0.01 |
| Tumor necrosis factor, alpha-induced protein 8 | BC005352 | 210260_s_at | 242.60 | 82.44 | -2.94 | 0.000064 | <0.01 |
| SP100 nuclear antigen | NM_003113 | 202864_s_at | 238.59 | 81.10 | -2.94 | 0.00048 | <0.01 |
| Tryptophanyl-tRNA synthetase | M61715 | 200628_s_at | 619.63 | 211.52 | -2.93 | 0.000002 | <0.01 |
| Major histocompatibility complex, class I, C | BC004489 | 208812_x_at | 2623.61 | 898.42 | -2.92 | 0.00035 | <0.01 |
| Tumor necrosis factor, alpha-induced protein 8 | NM_014350 | 208296_x_at | 259.86 | 89.03 | -2.92 | 0.00048 | <0.01 |
| Histone cluster 2, H2aa3 | AI313324 | 214290_s_at | 720.22 | 247.66 | -2.91 | 0.0011 | <0.01 |
| Interleukin 6 signal transducer (gp130) | AL049265 | 212195_at | 2080.57 | 725.27 | -2.87 | 0.00019 | <0.01 |
| Fibulin 1 | Z95331 | 202994_s_at | 545.28 | 189.83 | -2.87 | 0.00065 | <0.01 |
| Interferon regulatory factor 9 | NM_006084 | 203882_at | 307.42 | 107.38 | -2.86 | <0.000001 | <0.01 |
| Pleckstrin homology domain containing, family O member 2 | NM_025201 | 204436_at | 184.73 | 64.71 | -2.85 | 0.00082 | <0.01 |
| Thioredoxin interacting protein | NM_006472 | 201010_s_at | 535.81 | 187.88 | -2.85 | 0.0059 | <0.01 |
| Cathepsin C | NM_001814 | 201487_at | 550.75 | 194.65 | -2.83 | 0.00015 | <0.01 |
| Microfibrillar-associated protein 4 | R72286 | 212713_at | 578.43 | 204.89 | -2.82 | 0.00087 | <0.01 |
| AT rich interactive domain 5B (MRF1-like) | BG285011 | 212614_at | 1078.55 | 384.02 | -2.81 | 0.00019 | <0.01 |
| Spondin 2, extracellular matrix protein | NM_012445 | 218638_s_at | 634.94 | 226.78 | -2.80 | 0.021 | <0.01 |
| Chromosome 13 open reading frame 15 | NM_014059 | 218723_s_at | 212.39 | 76.00 | -2.79 | 0.00010 | <0.01 |
| Tumor necrosis factor, alpha-induced protein 6 | NM_007115 | 206026_s_at | 847.28 | 303.41 | -2.79 | 0.0046 | <0.01 |
| Tissue factor pathway inhibitor | AF021834 | 210664_s_at | 293.97 | 105.52 | -2.79 | 0.012 | <0.01 |
| Major histocompatibility complex, class I, B | L42024 | 209140_x_at | 3231.85 | 1163.91 | -2.78 | 0.00025 | <0.01 |
| Interleukin 7 receptor | NM_002185 | 205798_at | 484.14 | 174.18 | -2.78 | 0.019 | <0.01 |
| Proteasome activator subunit 1 | NM_006263 | 200814_at | 684.77 | 247.47 | -2.77 | 0.000009 | <0.01 |
| HEG homolog 1 (zebrafish) | AI148659 | 213069_at | 509.38 | 184.16 | -2.77 | 0.0027 | <0.01 |
| ATP-binding cassette, sub-family A (ABC1), member 1 | AF285167 | 203505_at | 278.42 | 101.37 | -2.75 | 0.00097 | <0.01 |
| Slit homolog 2 (Drosophila) | AF055585 | 209897_s_at | 244.95 | 88.95 | -2.75 | 0.011 | <0.01 |
| Histone cluster 2, H2aa3 | NM_003516 | 218280_x_at | 428.53 | 156.57 | -2.74 | 0.0010 | <0.01 |
| Fibulin 1 | NM_006486 | 202995_s_at | 448.92 | 164.51 | -2.73 | 0.0016 | <0.01 |
| ATP-binding cassette, sub-family A (ABC1), member 1 | NM_005502 | 203504_s_at | 339.68 | 126.53 | -2.68 | 0.000011 | <0.01 |
| Nicotinamide phosphoribosyltransferase | NM_005746 | 217739_s_at | 541.20 | 202.44 | -2.67 | 0.00068 | <0.01 |
| Insulin-like growth factor binding protein 6 | NM_002178 | 203851_at | 1312.90 | 491.34 | -2.67 | 0.0016 | <0.01 |
| Protein kinase inhibitor gamma | NM_007066 | 202732_at | 560.87 | 211.19 | -2.66 | 0.000008 | <0.01 |
| Thioredoxin interacting protein | AI439556 | 201009_s_at | 507.71 | 190.82 | -2.66 | 0.0088 | <0.01 |
| Tumor necrosis factor receptor superfamily, member 21 | NM_016629 | 218856_at | 247.55 | 93.51 | -2.65 | 0.0040 | <0.01 |
| Interferon induced transmembrane protein 2 (1-8D) | NM_006435 | 201315_x_at | 4413.29 | 1668.79 | -2.64 | <0.000001 | <0.01 |
| Matrix-remodelling associated 5 | AF245505 | 209596_at | 672.60 | 257.82 | -2.61 | 0.0016 | <0.01 |
| **Annotation** | **Accession** | **Probe Set ID** | **Control mean** | **SSc-ILD mean** | **Fold change** | **p value** | **q-value(%)** |
| UDP-glucose ceramide glucosyltransferase | NM_003358 | 204881_s_at | 614.44 | 236.46 | -2.60 | 0.00033 | <0.01 |
| Phosphatidic acid phosphatase type 2A | AB000888 | 209147_s_at | 454.68 | 174.69 | -2.60 | 0.0014 | <0.01 |
| Potassium channel tetramerisation domain containing 12 | AA551075 | 212188_at | 395.76 | 153.64 | -2.58 | 0.016 | <0.01 |
| Calcium/calmodulin-dependent protein kinase II inhibitor 1 | NM_018584 | 218309_at | 714.76 | 278.64 | -2.57 | 0.00039 | <0.01 |
| Optineurin | AV757675 | 202073_at | 193.33 | 75.56 | -2.56 | 0.00018 | <0.01 |
| Aldo-keto reductase family 1, member B1 | NM_001628 | 201272_at | 2024.30 | 789.86 | -2.56 | 0.00048 | <0.01 |
| Serpin peptidase inhibitor, clade F, member 1 | NM_002615 | 202283_at | 1165.35 | 463.93 | -2.51 | 0.0019 | <0.01 |
| Adenosine deaminase, RNA-specific | NM_001111 | 201786_s_at | 861.16 | 346.09 | -2.49 | 0.000002 | <0.01 |
| Major histocompatibility complex, class I, A | AA573862 | 215313_x_at | 2751.38 | 1110.07 | -2.48 | 0.000006 | <0.01 |
| N-acetylglucosamine-1-phosphate transferase, alpha and beta subunits | AK001821 | 212959_s_at | 209.96 | 84.77 | -2.48 | 0.0034 | <0.01 |
| Myeloid differentiation primary response gene (88) | U70451 | 209124_at | 262.90 | 106.41 | -2.47 | 0.00024 | <0.01 |
| Interleukin 8 | AF043337 | 211506_s_at | 799.00 | 324.04 | -2.47 | 0.0032 | <0.01 |
| CCAAT/enhancer binding protein (C/EBP), delta | NM_005195 | 203973_s_at | 748.34 | 303.41 | -2.47 | 0.0043 | <0.01 |
| Cbp/p300-interacting transactivator, with Glu/Asp-rich carboxyterminal domain,2 | AF109161 | 209357_at | 280.28 | 114.03 | -2.46 | 0.00026 | <0.01 |
| Tensin 3 | NM_022748 | 217853_at | 756.79 | 307.08 | -2.46 | 0.0013 | <0.01 |
| Granulin | AK023348 | 216041_x_at | 945.17 | 386.72 | -2.44 | 0.000063 | <0.01 |
| Interferon induced transmembrane protein 3 (1-8U) | BF338947 | 212203_x_at | 4971.02 | 2039.45 | -2.44 | <0.000001 | <0.01 |
| TNFAIP3 interacting protein 1 | NM_006058 | 207196_s_at | 386.23 | 158.71 | -2.43 | 0.00083 | <0.01 |
| Laminin, alpha 4 | NM_002290 | 202202_s_at | 741.79 | 306.34 | -2.42 | 0.000034 | <0.01 |
| Nicotinamide phosphoribosyltransferase | BF575514 | 217738_at | 330.31 | 136.38 | -2.42 | 0.0017 | <0.01 |
| Platelet-derived growth factor receptor, alpha polypeptide | NM_006206 | 203131_at | 2022.22 | 840.14 | -2.41 | 0.000003 | <0.01 |
| Stanniocalcin 2 | AI435828 | 203438_at | 621.16 | 257.79 | -2.41 | 0.0018 | <0.01 |
| Lymphocyte antigen 96 | NM_015364 | 206584_at | 229.75 | 95.62 | -2.40 | 0.00031 | <0.01 |
| Erythrocyte membrane protein band 4.1-like 2 | BF511685 | 201718_s_at | 198.21 | 82.75 | -2.40 | 0.0039 | <0.01 |
| Serine carboxypeptidase 1 | NM_021626 | 218217_at | 220.13 | 92.13 | -2.39 | 0.00026 | <0.01 |
| Phorbol-12-myristate-13-acetate-induced protein 1 | AI857639 | 204285_s_at | 420.65 | 176.48 | -2.38 | 0.000026 | <0.01 |
| Proteasome activator subunit 2 | NM_002818 | 201762_s_at | 802.34 | 337.56 | -2.38 | 0.000041 | <0.01 |
| Erythrocyte membrane protein band 4.1-like 2 | NM_001431 | 201719_s_at | 306.45 | 128.91 | -2.38 | 0.00039 | <0.01 |
| Monooxygenase, DBH-like 1 | AY007239 | 209708_at | 248.08 | 104.05 | -2.38 | 0.0032 | <0.01 |
| Major histocompatibility complex, class I, A | AI923492 | 213932_x_at | 2612.13 | 1102.23 | -2.37 | 0.000015 | <0.01 |
| Integral membrane protein 2B | AF092128 | 217732_s_at | 1312.72 | 555.97 | -2.36 | 0.000008 | <0.01 |
| Granulin | BC000324 | 211284_s_at | 827.12 | 350.51 | -2.36 | 0.000028 | <0.01 |
| B-cell CLL/lymphoma 6 | NM_001706 | 203140_at | 190.40 | 80.55 | -2.36 | 0.00016 | <0.01 |
| Cathepsin L1 | NM_001912 | 202087_s_at | 769.84 | 326.66 | -2.36 | 0.012 | <0.01 |
| Interleukin 8 | NM_000584 | 202859_x_at | 1540.90 | 656.27 | -2.35 | 0.00059 | <0.01 |
| Adducin 3 (gamma) | NM_019903 | 201753_s_at | 376.81 | 160.14 | -2.35 | 0.0049 | <0.01 |
| Lysophosphatidic acid receptor 1 | AW269335 | 204036_at | 635.12 | 273.16 | -2.33 | 0.0026 | <0.01 |
| SP100 nuclear antigen | NM_003113 | 202863_at | 183.83 | 79.27 | -2.32 | 0.000008 | <0.01 |
| CD47 molecule | BG230614 | 213857_s_at | 817.33 | 352.13 | -2.32 | 0.00024 | <0.01 |
| Ferritin, heavy polypeptide 1 | AA083483 | 214211_at | 1019.26 | 443.74 | -2.30 | 0.000004 | <0.01 |
| **Annotation** | **Accession** | **Probe Set ID** | **Control mean** | **SSc-ILD mean** | **Fold change** | **p value** | **q-value(%)** |
| Ninjurin 1 | NM_004148 | 203045_at | 221.58 | 96.20 | -2.30 | 0.000038 | <0.01 |
| Lipoma HMGIC fusion partner | NM_005780 | 218656_s_at | 262.95 | 114.35 | -2.30 | 0.0029 | <0.01 |
| Sequestosome 1 | NM_003900 | 201471_s_at | 1499.86 | 657.74 | -2.28 | 0.00019 | <0.01 |
| Glycophorin C | NM_002101 | 202947_s_at | 212.41 | 93.63 | -2.27 | 0.0017 | <0.01 |
| Laminin, beta 1 | NM_002291 | 201505_at | 551.79 | 244.06 | -2.26 | 0.00036 | <0.01 |
| FYN oncogene related to SRC, FGR, YES | M14333 | 210105_s_at | 372.83 | 164.63 | -2.26 | 0.0025 | <0.01 |
| Cyclin-dependent kinase inhibitor 2A (melanoma, p16, inhibits CDK4) | U38945 | 209644_x_at | 312.20 | 139.48 | -2.24 | 0.011 | <0.01 |
| Integral membrane protein 2B | NM_021999 | 217731_s_at | 688.86 | 310.07 | -2.22 | 0.0020 | <0.01 |
| Lysophosphatidic acid receptor 1 | BF055366 | 204037_at | 543.76 | 244.66 | -2.22 | 0.0075 | <0.01 |
| Epidermal growth factor receptor | AW157070 | 201983_s_at | 406.72 | 183.91 | -2.21 | 0.00092 | <0.01 |
| Aryl hydrocarbon receptor | NM_001621 | 202820_at | 216.93 | 99.16 | -2.19 | 0.00051 | <0.01 |
| Adducin 3 (gamma) | AI818488 | 205882_x_at | 445.76 | 203.48 | -2.19 | 0.0014 | <0.01 |
| Decorin | AI281593 | 209335_at | 2232.37 | 1027.07 | -2.17 | 0.00015 | <0.01 |
| Cytochrome P450, family 1, subfamily B, polypeptide 1 | AU144855 | 202436_s_at | 467.47 | 216.00 | -2.16 | 0.037 | <0.01 |
| Adducin 3 (gamma) | AI763123 | 201752_s_at | 469.13 | 218.24 | -2.15 | 0.00078 | <0.01 |
| SAM and SH3 domain containing 1 | AK025495 | 213236_at | 225.16 | 104.68 | -2.15 | 0.0018 | <0.01 |
| DNA-damage-inducible transcript 3 | BC003637 | 209383_at | 285.01 | 133.09 | -2.14 | 0.00069 | <0.01 |
| Heme binding protein 1 | NM_015987 | 218450_at | 475.01 | 223.29 | -2.13 | 0.0015 | <0.01 |
| Adducin 3 (gamma) | BE545756 | 201034_at | 520.40 | 243.82 | -2.13 | 0.0041 | <0.01 |
| Sulfatase 1 | BE500977 | 212354_at | 601.25 | 283.95 | -2.12 | 0.011 | <0.01 |
| Perilipin 2 | BC005127 | 209122_at | 477.98 | 226.55 | -2.11 | 0.00012 | <0.01 |
| Alcohol dehydrogenase 5 (class III), chi polypeptide | M30471 | 208848_at | 260.04 | 124.11 | -2.10 | 0.000011 | <0.01 |
| PDZ domain containing ring finger 3 | AL569804 | 212915_at | 193.21 | 91.88 | -2.10 | 0.000079 | <0.01 |
| Syndecan binding protein (syntenin) | NM_005625 | 200958_s_at | 1404.06 | 669.35 | -2.10 | 0.000094 | <0.01 |
| EGF-containing fibulin-like extracellular matrix protein 1 | AI826799 | 201842_s_at | 970.26 | 463.10 | -2.10 | 0.0014 | <0.01 |
| Dihydropyrimidinase-like 2 | NM_001386 | 200762_at | 552.25 | 265.70 | -2.08 | 0.000009 | <0.01 |
| Sarcoglycan, epsilon | NM_003919 | 204688_at | 421.32 | 202.84 | -2.08 | 0.00062 | <0.01 |
| Laminin, beta 1 | M20206 | 211651_s_at | 404.45 | 194.10 | -2.08 | 0.0026 | <0.01 |
| FK506 binding protein 9, 63 kDa | AL050187 | 212169_at | 735.67 | 354.16 | -2.08 | 0.015 | <0.01 |
| Granulin | NM_002087 | 200678_x_at | 544.33 | 261.34 | -2.08 | 0.019 | <0.01 |
| Phosphatidic acid phosphatase type 2A | AF014403 | 210946_at | 662.73 | 320.62 | -2.07 | 0.00039 | <0.01 |
| Acyl-CoA synthetase long-chain family member 3 | D89053 | 201662_s_at | 286.97 | 138.73 | -2.07 | 0.0011 | <0.01 |
| Decorin | AF138303 | 211813_x_at | 6527.54 | 3165.57 | -2.06 | 0.000011 | <0.01 |
| Optineurin | NM_021980 | 202074_s_at | 573.06 | 279.49 | -2.05 | 0.00004 | <0.01 |
| Tripartite motif-containing 8 | NM_030912 | 221012_s_at | 368.69 | 179.84 | -2.05 | 0.000089 | <0.01 |
| Related RAS viral (r-ras) oncogene homolog 2 | AI753792 | 212589_at | 314.11 | 154.21 | -2.04 | 0.0011 | <0.01 |
| Cathepsin B | NM_001908 | 200838_at | 1294.18 | 638.23 | -2.03 | 0.0053 | <0.01 |
| CD47 molecule | Z25521 | 211075_s_at | 504.30 | 249.18 | -2.02 | 0.000039 | <0.01 |
| Chromosome 14 open reading frame 132 | NM_020215 | 218820_at | 300.39 | 148.51 | -2.02 | 0.0058 | <0.01 |
| Receptor accessory protein 5 | BC000232 | 208873_s_at | 358.34 | 177.89 | -2.01 | 0.0024 | <0.01 |
| **Annotation** | **Accession** | **Probe Set ID** | **Control mean** | **SSc-ILD mean** | **Fold change** | **p value** | **q-value(%)** |
| Hypothetical protein LOC100130633 | AI805560 | 213698_at | 240.54 | 120.27 | -2.00 | 0.00034 | <0.01 |
| Hexosaminidase B (beta polypeptide) | NM_000521 | 201944_at | 728.46 | 363.34 | -2.00 | 0.011 | <0.01 |
| Histamine N-methyltransferase | NM_006895 | 204112_s_at | 174.98 | 35.22 | -4.97 | 0.012 | 0.012 |
| Cholesterol 25-hydroxylase | NM_003956 | 206932_at | 215.09 | 62.62 | -3.43 | 0.019 | 0.012 |
| Sphingomyelin phosphodiesterase, acid-like 3A | AA873600 | 213624_at | 253.49 | 76.42 | -3.32 | 0.018 | 0.012 |
| Tissue factor pathway inhibitor | J03225 | 209676_at | 265.88 | 86.55 | -3.07 | 0.018 | 0.012 |
| REV3-like, catalytic subunit of DNA polymerase zeta (yeast) | NM_002912 | 208070_s_at | 168.81 | 68.18 | -2.48 | 0.0016 | 0.012 |
| Meis homeobox 3 pseudogene 1 | H15129 | 214077_x_at | 185.97 | 76.37 | -2.44 | 0.0041 | 0.012 |
| Fibulin 1 | NM_001996 | 201787_at | 343.70 | 155.90 | -2.20 | 0.026 | 0.012 |
| Transmembrane protein 47 | AI803181 | 209655_s_at | 313.65 | 151.74 | -2.07 | 0.015 | 0.012 |
| CASP8 and FADD-like apoptosis regulator | AF009616 | 211316_x_at | 227.40 | 112.45 | -2.02 | 0.0073 | 0.012 |
| CD82 molecule | NM_002231 | 203904_x_at | 245.45 | 122.53 | -2.00 | 0.0072 | 0.012 |
| Transmembrane protein 176B | NM_014020 | 220532_s_at | 235.62 | 9.53 | -24.73 | 0.049 | 0.022 |
| Transmembrane protein 176A | NM_018487 | 218345_at | 138.95 | 7.60 | -18.27 | 0.026 | 0.022 |
| Alcohol dehydrogenase 1B (class I), beta polypeptide | AF153821 | 209614_at | 110.38 | 7.41 | -14.89 | 0.011 | 0.022 |
| Retinoic acid receptor responder (tazarotene induced) 1 | NM_002888 | 206392_s_at | 253.84 | 27.91 | -9.09 | 0.044 | 0.022 |
| Tumor necrosis factor (ligand) superfamily, member 10 | NM_003810 | 202688_at | 139.35 | 15.74 | -8.85 | 0.028 | 0.022 |
| Hemoglobin, gamma A | NM_000559 | 204848_x_at | 157.97 | 19.84 | -7.96 | 0.022 | 0.022 |
| Retinoic acid receptor responder (tazarotene induced) 1 | AI669229 | 221872_at | 221.36 | 28.12 | -7.87 | 0.046 | 0.022 |
| 2'-5'-oligoadenylate synthetase-like | AF063612 | 210797_s_at | 249.07 | 33.74 | -7.38 | 0.044 | 0.022 |
| G protein-coupled receptor 37 (endothelin receptor type B-like) | U87460 | 209631_s_at | 170.94 | 23.29 | -7.34 | 0.022 | 0.022 |
| Hemoglobin, gamma A | NM_000184 | 204419_x_at | 177.37 | 29.49 | -6.01 | 0.027 | 0.022 |
| Hemoglobin, gamma A | AI133353 | 213515_x_at | 182.98 | 32.54 | -5.62 | 0.026 | 0.022 |
| Transcription factor 21 | NM_003206 | 204931_at | 283.95 | 51.58 | -5.51 | 0.039 | 0.022 |
| Phosphodiesterase 5A, cGMP-specific | NM_001083 | 206757_at | 137.12 | 27.99 | -4.90 | 0.018 | 0.022 |
| Fibroblast growth factor 7 | NM_002009 | 205782_at | 159.10 | 49.53 | -3.21 | 0.015 | 0.022 |
| G0/G1switch 2 | NM_015714 | 213524_s_at | 244.86 | 90.92 | -2.69 | 0.028 | 0.022 |
| Coagulation factor II (thrombin) receptor | NM_001992 | 203989_x_at | 264.80 | 106.92 | -2.48 | 0.033 | 0.022 |
| Rho-related BTB domain containing 3 | NM_014899 | 202976_s_at | 271.18 | 121.05 | -2.24 | 0.029 | 0.022 |
| Aldehyde dehydrogenase 2 family (mitochondrial) | NM_000690 | 201425_at | 396.35 | 195.53 | -2.03 | 0.047 | 0.022 |
| H2B histone family, member S | NM_017445 | 208579_x_at | 280.55 | 140.12 | -2.00 | 0.026 | 0.022 |
| Tenascin XA pseudogene | BE044614 | 213451_x_at | 176.99 | 33.03 | -5.36 | 0.045 | 0.030 |
| Tenascin XA pseudogene | M25813 | 216333_x_at | 158.43 | 32.60 | -4.86 | 0.045 | 0.030 |
| Tenascin XA pseudogene | NM_007116 | 206093_x_at | 174.54 | 36.65 | -4.76 | 0.044 | 0.030 |
| Heat-responsive protein 12 | N54448 | 203790_s_at | 208.75 | 80.66 | -2.59 | 0.039 | 0.030 |
| Dipeptidyl-peptidase 4 | M74777 | 211478_s_at | 216.90 | 85.20 | -2.55 | 0.042 | 0.030 |
| Prostaglandin D2 synthase 21kDa (brain) | BC005939 | 211748_x_at | 209.41 | 85.49 | -2.45 | 0.034 | 0.030 |
| Growth differentiation factor 15 | AF003934 | 221577_x_at | 185.79 | 84.93 | -2.19 | 0.041 | 0.030 |
| Prostaglandin D2 synthase 21kDa (brain) | NM_000954 | 212187_x_at | 229.64 | 109.74 | -2.09 | 0.049 | 0.030 |
| Histone cluster 1, H2bd | BC002842 | 209911_x_at | 258.85 | 126.90 | -2.04 | 0.036 | 0.030 |
| **Annotation** | **Accession** | **Probe Set ID** | **Control mean** | **SSc-ILD mean** | **Fold change** | **p value** | **q-value(%)** |
| C-type lectin domain family 3, member B | NM_003278 | 205200_at | 144.76 | 42.80 | -3.38 | 0.041 | 0.048 |

**Additional file 1. Genes differentially expressed in SSc-ILD.** Word file, .txt extension.

This data set contains all of the genes up- or down- regulated (according to the criteria described in the methods) in SSc-ILD fibroblasts compared to control fibroblasts. Included are p-values from dChip analysis and q-values from SAM analysis.
