# Supplementary material for: Microarray profiling reveals suppressed interferon stimulated gene program in fibroblasts from scleroderma-associated interstitial lung disease
Source: Respir Res. 2013 Aug 2;14(1):80. doi: 10.1186/1465-9921-14-80 (PMC3750263; doi:10.1186/1465-9921-14-80)
Supplement: Additional file 3 — Genes differentially expressed between IPF and SSc-ILD. Word file, .txt extension. This data set contains all of the genes up- or down- regulated in IPF fibroblasts compared to SSc-ILD fibroblasts. Included are p-values from dChip analysis and q-values from SAM analysis. [file 1465-9921-14-80-S3.docx]

| **Annotation** | **Accession** | **Probe Set ID** | **Control mean** | **SSc mean** | **IPF mean** | **Fold change**  (IPF vs SSc) | **p value** | **q-value(%)** |
| --- | --- | --- | --- | --- | --- | --- | --- | --- |
| Chromosome 21 open reading frame 7 | NM_020152 | 221211_s_at | 117.54 | 67.45 | 299.76 | 4.44 | 0.0016 | <0.01 |
| Interleukin 11 | NM_000641 | 206924_at | 23.62 | 717.79 | 2374.92 | 3.31 | 0.000074 | <0.01 |
| Serglycin | NM_002727 | 201859_at | 816.74 | 228.32 | 671.17 | 2.94 | 0.00583 | <0.01 |
| Versican | R94644 | 215646_s_at | 141.85 | 353.27 | 827.52 | 2.34 | 0.00039 | <0.01 |
| Secreted protein, acidic, cysteine-rich (osteonectin) | AL575922 | 212667_at | 878.92 | 845.52 | 1899.52 | 2.25 | 0.00016 | <0.01 |
| Tensin 1 | NM_018274 | 221246_x_at | 211.87 | 320.59 | 702.52 | 2.19 | 0.0018 | <0.01 |
| Tensin 1 | AL046979 | 221748_s_at | 232.17 | 380.64 | 832.15 | 2.19 | 0.0057 | <0.01 |
| Sparc/osteonectin, cwcv and kazal-like domains proteoglycan (testican) 1 | AF231124 | 202363_at | 587.91 | 678.57 | 1417.8 | 2.09 | 0.0011 | <0.01 |

**Additional file 3. Genes differentially expressed between IPF and SSc-ILD.** Word file, .txt extension.

This data set contains all of the genes up- or down- regulated (according to the criteria described in the methods) in IPF fibroblasts compared to SSc-ILD fibroblasts. Included are p-values from dChip analysis and q-values from SAM analysis.
