# Supplementary material for: Microarray profiling reveals suppressed interferon stimulated gene program in fibroblasts from scleroderma-associated interstitial lung disease
Source: Respir Res. 2013 Aug 2;14(1):80. doi: 10.1186/1465-9921-14-80 (PMC3750263; doi:10.1186/1465-9921-14-80)
Supplement: Additional file 4 — Summary of differentially expressed genes in SSc-ILD and IPF. Word file, .txt extension. This data set contains all of the genes up- or down- regulated in fibroblasts from at least one disease group compared to control fibroblasts. Included are p-values from dChip analysis and q-values from SAM analysis. [file 1465-9921-14-80-S4.docx]

|  |  |  |  | **SSc-ILD** | | | | **IPF** | | | |
| --- | --- | --- | --- | --- | --- | --- | --- | --- | --- | --- | --- |
| **Annotation** | **Accession** | **Probe Set ID** | **Control mean** | **Ssc-ILD mean** | **Fold change** | **p value** | **q-value(%)** | **IPF mean** | **Fold change** | **p value** | **q-value(%)** |
| **Overexpressed in SSc-ILD and/or IPF** | | | | | | | | | | | |
| --- | AI278204 | 214807_at | 44.56 | 84.29 | 1.89 | 0.20 | 0.55 | 273.17 | 6.13 | 0.019 | <0.01 |
| --- | D50604 | 217211_at | 80.72 | 176.45 | 2.19 | 0.0082 | 0.29 | 256.00 | 3.17 | 0.000001 | <0.01 |
| 5'-nucleotidase domain containing 2 | NM_022908 | 218051_s_at | 51.71 | 152.69 | 2.95 | 0.00028 | 0.0303 | 201.66 | 3.90 | 0.013 | <0.01 |
| Abhydrolase domain containing 2 | BE671816 | 221815_at | 80.55 | 139.23 | 1.73 | 0.031 | 0.504 | 194.90 | 2.42 | 0.000018 | 0.028 |
| Abhydrolase domain containing 2 | AI557319 | 63825_at | 130.30 | 266.81 | 2.05 | 0.091 | 0.34 | 305.58 | 2.35 | 0.016 | 0.05 |
| Actin, alpha 2, smooth muscle, aorta | NM_001613 | 200974_at | 820.33 | 2831.14 | 3.45 | 0.023 | <0.01 | 5456.04 | 6.65 | <0.000001 | <0.01 |
| Actin, alpha, cardiac muscle 1 | NM_005159 | 205132_at | 107.70 | 462.17 | 4.29 | 0.10 | 0.069 | 638.01 | 5.92 | 0.0035 | <0.01 |
| Actin, gamma 2, smooth muscle, enteric | NM_001615 | 202274_at | 384.95 | 1169.72 | 3.04 | 0.24 | 0.11 | 2521.49 | 6.55 | 0.000002 | <0.01 |
| Actinin, alpha 1 | BC003576 | 208637_x_at | 259.33 | 459.34 | 1.77 | 0.023 | 0.054 | 559.53 | 2.16 | 0.016 | <0.01 |
| Actinin, alpha 1 | M95178 | 211160_x_at | 199.09 | 355.60 | 1.79 | 0.029 | 0.054 | 432.61 | 2.17 | 0.011 | <0.01 |
| Actinin, alpha 4 | U48734 | 200601_at | 102.29 | 197.04 | 1.93 | 0.0041 | 0.29 | 207.34 | 2.03 | 0.010 | 0.028 |
| ADAM metallopeptidase domain 12 | NM_003474 | 202952_s_at | 128.25 | 376.64 | 2.94 | 0.013 | 0.0303 | 559.67 | 4.36 | <0.000001 | <0.01 |
| ADAM metallopeptidase domain 12 | W46291 | 213790_at | 65.29 | 175.65 | 2.69 | 0.10 | 0.11 | 288.95 | 4.43 | 0.018 | <0.01 |
| ADAM metallopeptidase domain 19 | Y13786 | 209765_at | 60.22 | 194.06 | 3.22 | 0.0046 | 0.048 | 337.01 | 5.60 | 0.022 | <0.01 |
| Adhesion molecule with Ig-like domain 2 | AC004010 | 222108_at | 54.71 | 545.04 | 9.96 | 0.0072 | <0.01 | 490.55 | 8.97 | 0.025 | <0.01 |
| Aldehyde dehydrogenase 1 family, member B1 | BC001619 | 209646_x_at | 58.83 | 102.63 | 1.74 | 0.014 | 0.54 | 165.12 | 2.81 | 0.029 | 0.016 |
| Amyloid beta (A4) precursor protein-binding, family B, member 2 | AI694303 | 212970_at | 52.90 | 63.28 | 1.20 | 0.087 | 0.56 | 153.25 | 2.90 | 0.00063 | <0.01 |
| Amyloid beta (A4) precursor protein-binding, family B, member 2 | BF115739 | 212985_at | 61.05 | 90.54 | 1.48 | 0.088 | 0.56 | 192.52 | 3.15 | 0.015 | <0.01 |
| Apoptosis, caspase activation inhibitor | NM_020371 | 219366_at | 62.44 | 120.83 | 1.94 | 0.0012 | 0.46 | 174.29 | 2.79 | 0.002 | 0.0096 |
| ARP3 actin-related protein 3 homolog (yeast) | NM_005721 | 200996_at | 378.36 | 665.77 | 1.76 | 0.032 | 0.048 | 763.97 | 2.02 | 0.000004 | <0.01 |
| Asparagine synthetase (glutamine-hydrolyzing) | NM_001673 | 205047_s_at | 166.22 | 322.80 | 1.94 | 0.059 | 0.069 | 496.92 | 2.99 | 0.022 | <0.01 |
| ATP citrate lyase | AI971281 | 201127_s_at | 206.28 | 335.81 | 1.63 | 0.0083 | 0.054 | 450.21 | 2.18 | 0.023 | <0.01 |
| ATP citrate lyase | U18197 | 210337_s_at | 171.95 | 360.98 | 2.10 | 0.0019 | 0.0303 | 410.64 | 2.39 | 0.000029 | <0.01 |
| ATPase, Na+/K+ transporting, beta 1 polypeptide | BC000006 | 201242_s_at | 77.89 | 215.08 | 2.76 | 0.00066 | 0.0303 | 361.21 | 4.64 | 0.057 | <0.01 |
| Basic helix-loop-helix family, member e40 | NM_003670 | 201170_s_at | 44.27 | 256.07 | 5.78 | 0.0014 | <0.01 | 282.04 | 6.37 | 0.012 | <0.01 |
| Biglycan | BC002416 | 201261_x_at | 302.28 | 549.66 | 1.82 | 0.035 | 0.054 | 744.23 | 2.46 | 0.013 | <0.01 |
| Biglycan | NM_001711 | 201262_s_at | 47.64 | 133.75 | 2.81 | 0.031 | 0.401 | 196.18 | 4.12 | 0.016 | <0.01 |
| Biglycan | AA845258 | 213905_x_at | 202.01 | 319.52 | 1.58 | 0.068 | 0.104 | 444.33 | 2.20 | 0.0031 | 0.0096 |
| BolA homolog 2 (E. coli) | AF060511 | 209836_x_at | 176.86 | 274.06 | 1.55 | 0.0017 | 0.29 | 369.48 | 2.09 | 0.025 | <0.01 |
| C2 calcium-dependent domain containing 2 | AP001745 | 212875_s_at | 60.28 | 95.22 | 1.58 | 0.03 | 0.55 | 176.93 | 2.94 | 0.013 | <0.01 |
| Cadherin 2, type 1, N-cadherin (neuronal) | M34064 | 203440_at | 105.26 | 432.98 | 4.11 | 0.00095 | <0.01 | 459.98 | 4.37 | 0.012 | <0.01 |
| Cadherin 2, type 1, N-cadherin (neuronal) | NM_001792 | 203441_s_at | 45.15 | 163.70 | 3.63 | 0.0099 | 0.054 | 123.84 | 2.74 | 0.0073 | 0.097 |
| Cadherin 6, type 2, K-cadherin (fetal kidney) | AU151483 | 205532_s_at | 27.73 | 77.59 | 2.80 | 0.098 | 0.54 | 215.38 | 7.77 | 0.0038 | <0.01 |
| Cadherin 6, type 2, K-cadherin (fetal kidney) | BC000019 | 210602_s_at | 26.23 | 130.57 | 4.98 | 0.085 | 0.094 | 298.41 | 11.38 | 0.00092 | <0.01 |
| Cadherin 6, type 2, K-cadherin (fetal kidney) | BF344237 | 214803_at | 78.50 | 216.01 | 2.75 | 0.21 | 0.15 | 697.03 | 8.88 | <0.000001 | <0.01 |
| Calbindin 2 | NM_001740 | 205428_s_at | 21.04 | 58.59 | 2.78 | 0.025 | 0.55 | 130.07 | 6.18 | 0.01 | <0.01 |
| Caldesmon 1 | AI685060 | 201615_x_at | 679.86 | 1027.02 | 1.51 | 0.15 | 0.086 | 1622.82 | 2.39 | 0.000001 | <0.01 |
| Caldesmon 1 | AL577531 | 201616_s_at | 662.71 | 1210.70 | 1.83 | 0.14 | 0.069 | 1471.86 | 2.22 | 0.00023 | <0.01 |
| Caldesmon 1 | NM_004342 | 201617_x_at | 376.13 | 578.28 | 1.54 | 0.14 | 0.094 | 878.12 | 2.33 | 0.0065 | <0.01 |
| Calmodulin binding transcription activator 2 | AB020716 | 212948_at | 111.01 | 136.39 | 1.23 | 0.37 | 0.56 | 224.61 | 2.02 | 0.015 | 0.05 |
| Calponin 1, basic, smooth muscle | NM_001299 | 203951_at | 83.00 | 430.22 | 5.18 | 0.12 | 0.069 | 1047.98 | 12.63 | 0.0024 | <0.01 |
| cAMP responsive element binding protein 3-like 2 | BE675139 | 212345_s_at | 290.05 | 612.65 | 2.11 | 0.011 | <0.01 | 890.40 | 3.07 | 0.0062 | <0.01 |
| CAP, adenylate cyclase-associated protein 1 (yeast) | NM_006367 | 200625_s_at | 465.25 | 759.89 | 1.63 | 0.0023 | <0.01 | 1031.06 | 2.22 | 0.0071 | <0.01 |
|  |  |  |  | **SSc-ILD** | | | | **IPF** | | | |
| **Annotation** | **Accession** | **Probe Set ID** | **Control mean** | **Ssc-ILD mean** | **Fold change** | **p value** | **q-value(%)** | **IPF mean** | **Fold change** | **p value** | **q-value(%)** |
| CAP, adenylate cyclase-associated protein 1 (yeast) | AA806142 | 213798_s_at | 636.17 | 1012.10 | 1.59 | 0.0024 | <0.01 | 1416.86 | 2.23 | 0.0011 | <0.01 |
| CDK5 regulatory subunit associated protein 2 | NM_018249 | 220935_s_at | 75.99 | 86.63 | 1.14 | 0.28 | 0.504 | 178.53 | 2.35 | <0.000001 | 0.016 |
| Cell adhesion molecule 1 | NM_014333 | 209030_s_at | 141.18 | 376.45 | 2.67 | 0.13 | 0.086 | 520.79 | 3.69 | 0.000008 | <0.01 |
| Cell adhesion molecule 1 | AL519710 | 209031_at | 100.27 | 219.90 | 2.19 | 0.11 | 0.104 | 558.86 | 5.57 | 0.0092 | <0.01 |
| Cell adhesion molecule 1 | AF132811 | 209032_s_at | 32.89 | 65.82 | 2.00 | 0.12 | 0.56 | 160.85 | 4.89 | 0.00058 | <0.01 |
| Cell division cycle 20 homolog (S. cerevisiae) | NM_001255 | 202870_s_at | 20.69 | 143.24 | 6.92 | 0.042 | 0.069 | 113.43 | 5.48 | 0.28 | 0.17 |
| Centromere protein F, 350/400kDa (mitosin) | NM_005196 | 207828_s_at | 61.18 | 207.92 | 3.40 | 0.013 | 0.054 | 185.30 | 3.03 | 0.24 | 0.073 |
| Chaperonin containing TCP1, subunit 2 (beta) | AL545982 | 201946_s_at | 253.49 | 419.19 | 1.65 | 0.0017 | 0.022 | 532.48 | 2.10 | 0.012 | <0.01 |
| Chondroitin sulfate synthase 1 | NM_014918 | 203044_at | 109.10 | 193.53 | 1.77 | 0.014 | 0.401 | 263.96 | 2.42 | 0.038 | 0.0096 |
| Chromosome 16 open reading frame 61 | NM_020188 | 218447_at | 121.81 | 235.43 | 1.93 | 0.0015 | 0.0303 | 293.34 | 2.41 | 0.033 | <0.01 |
| Chromosome 21 open reading frame 7 | NM_020152 | 221211_s_at | 117.54 | 67.45 | -1.74 | 0.039 | 0.41 | 299.76 | 2.55 | 0.0013 | <0.01 |
| Chromosome 5 open reading frame 13 | U36189 | 201309_x_at | 193.25 | 552.20 | 2.86 | 0.0032 | <0.01 | 789.60 | 4.09 | 0.000073 | <0.01 |
| Chromosome 5 open reading frame 13 | NM_004772 | 201310_s_at | 430.04 | 1233.67 | 2.87 | 0.0053 | <0.01 | 1431.91 | 3.33 | 0.012 | <0.01 |
| Chromosome 6 open reading frame 145 | AK024828 | 212923_s_at | 215.95 | 434.40 | 2.01 | 0.062 | 0.054 | 681.57 | 3.16 | 0.0019 | <0.01 |
| Cleavage and polyadenylation specific factor 1 | NM_013291 | 201639_s_at | 83.74 | 156.18 | 1.87 | 0.0067 | 0.44 | 201.77 | 2.41 | 0.012 | 0.016 |
| Coactosin-like 1 (Dictyostelium) | NM_021615 | 221059_s_at | 185.76 | 360.97 | 1.94 | 0.0048 | 0.0303 | 560.57 | 3.02 | 0.0015 | <0.01 |
| Coiled-coil domain containing 99 | AF269167 | 221685_s_at | 85.14 | 373.33 | 4.38 | 0.0018 | <0.01 | 501.03 | 5.88 | 0.0011 | <0.01 |
| Collagen, type IV, alpha 1 | AI922605 | 211980_at | 365.60 | 870.69 | 2.38 | 0.048 | 0.048 | 1639.46 | 4.48 | 0.0019 | <0.01 |
| Collagen, type IV, alpha 1 | NM_001845 | 211981_at | 222.46 | 650.12 | 2.92 | 0.053 | 0.048 | 1235.74 | 5.55 | 0.0046 | <0.01 |
| Collagen, type IV, alpha 2 | X05610 | 211964_at | 512.30 | 879.85 | 1.72 | 0.11 | 0.069 | 1466.16 | 2.86 | 0.000005 | <0.01 |
| Collagen, type IV, alpha 2 | AA909035 | 211966_at | 165.69 | 419.59 | 2.53 | 0.083 | 0.069 | 667.25 | 4.03 | <0.000001 | <0.01 |
| Collagen, type V, alpha 1 | N30339 | 212488_at | 528.69 | 1455.41 | 2.75 | 0.016 | <0.01 | 1767.20 | 3.34 | 0.00041 | <0.01 |
| Collagen, type V, alpha 1 | AI983428 | 212489_at | 219.71 | 627.74 | 2.86 | 0.015 | <0.01 | 691.21 | 3.15 | <0.000001 | <0.01 |
| Collagen, type VII, alpha 1 | NM_000094 | 204136_at | 81.29 | 279.94 | 3.44 | 0.029 | 0.054 | 313.89 | 3.86 | 0.00025 | <0.01 |
| Collagen, type XI, alpha 1 | NM_001854 | 204320_at | 31.27 | 145.79 | 4.66 | 0.024 | 0.069 | 40.09 | 1.28 | 0.49 | 0.32 |
| Connective tissue growth factor | M92934 | 209101_at | 467.87 | 2637.13 | 5.64 | 0.00068 | <0.01 | 3201.08 | 6.84 | 0.00041 | <0.01 |
| CTP synthase | NM_001905 | 202613_at | 39.48 | 245.10 | 6.21 | 0.0061 | 0.012 | 454.71 | 11.52 | 0.000059 | <0.01 |
| Cyclin A2 | NM_001237 | 203418_at | 26.97 | 128.99 | 4.78 | 0.0044 | 0.054 | 136.54 | 5.06 | 0.065 | 0.016 |
| Cyclin B1 | BE407516 | 214710_s_at | 42.74 | 180.59 | 4.23 | 0.032 | 0.054 | 166.25 | 3.89 | 0.24 | 0.05 |
| Cyclin B2 | NM_004701 | 202705_at | 49.44 | 165.79 | 3.35 | 0.013 | 0.054 | 134.58 | 2.72 | 0.29 | 0.26 |
| Cyclin-dependent kinase 1 | AL524035 | 203213_at | 42.19 | 171.61 | 4.07 | 0.011 | 0.054 | 181.29 | 4.30 | 0.18 | 0.028 |
| Cyclin-dependent kinase 1 | NM_001786 | 203214_x_at | 46.80 | 177.95 | 3.80 | 0.012 | 0.054 | 210.37 | 4.49 | 0.15 | 0.016 |
| Cyclin-dependent kinase 1 | D88357 | 210559_s_at | 52.88 | 204.47 | 3.87 | 0.0094 | 0.048 | 253.21 | 4.79 | 0.14 | <0.01 |
| Cysteine and glycine-rich protein 1 | NM_004078 | 200621_at | 457.62 | 949.86 | 2.08 | 0.12 | 0.069 | 1889.72 | 4.13 | 0.000033 | <0.01 |
| Cysteine and glycine-rich protein 2 | NM_001321 | 207030_s_at | 315.55 | 775.45 | 2.46 | 0.056 | 0.054 | 1011.12 | 3.20 | 0.00066 | <0.01 |
| Cysteine and glycine-rich protein 2 | U46006 | 211126_s_at | 124.93 | 341.87 | 2.74 | 0.012 | 0.0303 | 377.02 | 3.02 | 0.0023 | <0.01 |
| Cysteine-rich secretory protein LCCL domain containing 2 | AL136861 | 221541_at | 227.95 | 427.74 | 1.88 | 0.18 | 0.11 | 596.87 | 2.62 | 0.0019 | <0.01 |
| Cytokine receptor-like factor 1 | NM_004750 | 206315_at | 23.43 | 128.54 | 5.49 | 0.0042 | 0.054 | 319.30 | 13.63 | 0.0098 | <0.01 |
| Dapper, antagonist of beta-catenin, homolog 1 (Xenopus laevis) | NM_016651 | 219179_at | 33.34 | 173.36 | 5.20 | 0.0027 | 0.0303 | 271.00 | 8.13 | 0.001 | <0.01 |
| DEAD (Asp-Glu-Ala-Asp) box polypeptide 3, Y-linked | NM_004660 | 205000_at | 26.28 | 56.65 | 2.16 | 0.27 | 0.56 | 141.63 | 5.39 | 0.027 | 0.028 |
| Deleted in liver cancer 1 | AF026219 | 210762_s_at | 367.83 | 605.35 | 1.65 | 0.06 | 0.069 | 845.90 | 2.30 | 0.000016 | <0.01 |
| Desmoplakin | NM_004415 | 200606_at | 80.51 | 306.14 | 3.80 | 0.0016 | <0.01 | 55.81 | -1.44 | 0.29 | 0.29 |
| Dual specificity phosphatase 1 | NM_004417 | 201041_s_at | 286.93 | 669.72 | 2.33 | 0.059 | 0.054 | 1003.03 | 3.50 | 0.00024 | <0.01 |
| Dynamin 1-like | NM_012062 | 203105_s_at | 81.53 | 138.48 | 1.70 | 0.0075 | 0.504 | 181.92 | 2.23 | 0.011 | 0.016 |
|  |  |  |  | **SSc-ILD** | | | | **IPF** | | | |
| **Annotation** | **Accession** | **Probe Set ID** | **Control mean** | **Ssc-ILD mean** | **Fold change** | **p value** | **q-value(%)** | **IPF mean** | **Fold change** | **p value** | **q-value(%)** |
| Ectodermal-neural cortex 1 | AF010314 | 201340_s_at | 61.59 | 154.56 | 2.51 | 0.032 | 0.401 | 248.79 | 4.04 | 0.023 | <0.01 |
| Ectodermal-neural cortex 1 (with BTB-like domain) | NM_003633 | 201341_at | 98.60 | 198.07 | 2.01 | 0.0099 | 0.29 | 327.45 | 3.32 | 0.00019 | <0.01 |
| Ectonucleotide pyrophosphatase/phosphodiesterase 1 | NM_006208 | 205066_s_at | 109.70 | 296.12 | 2.70 | 0.013 | 0.048 | 172.32 | 1.57 | 0.038 | 0.32 |
| Elastin | AA479278 | 212670_at | 43.66 | 396.92 | 9.09 | 0.0021 | <0.01 | 766.87 | 17.56 | 0.0026 | <0.01 |
| Endothelial cell-specific molecule 1 | NM_007036 | 208394_x_at | 10.21 | 64.32 | 6.30 | 0.19 | 0.54 | 116.77 | 11.43 | 0.031 | 0.0096 |
| Eukaryotic translation initiation factor 1A, Y-linked | BC005248 | 204409_s_at | 25.19 | 63.05 | 2.50 | 0.16 | 0.55 | 139.95 | 5.55 | 0.0041 | 0.016 |
| Eukaryotic translation initiation factor 4E binding protein 1 | AB044548 | 221539_at | 104.34 | 230.53 | 2.21 | 0.01 | 0.054 | 281.30 | 2.70 | 0.0069 | <0.01 |
| Eukaryotic translation initiation factor 5A | NM_001970 | 201123_s_at | 443.74 | 737.14 | 1.66 | 0.13 | 0.094 | 965.64 | 2.18 | 0.0063 | 0.0096 |
|  |  |  |  |  | | | |  | | | |
| Eukaryotic translation initiation factor 5A | BF541557 | 213753_x_at | 205.04 | 163.22 | -1.26 | 0.50 | 0.41 | 496.63 | 2.42 | 0.013 | 0.0096 |
| Fatty acid binding protein 5 | NM_001444 | 202345_s_at | 47.30 | 178.07 | 3.76 | 0.033 | 0.054 | 97.05 | 2.05 | 0.16 | 0.32 |
| Fermitin family member 2 | AW469573 | 209209_s_at | 117.61 | 265.36 | 2.26 | 0.016 | 0.054 | 455.22 | 3.87 | 0.0041 | <0.01 |
| Fermitin family member 2 | Z24725 | 209210_s_at | 500.10 | 898.00 | 1.80 | 0.065 | 0.054 | 1644.63 | 3.29 | 0.0056 | <0.01 |
| Fermitin family member 2 | AI928241 | 214212_x_at | 229.56 | 454.55 | 1.98 | 0.077 | 0.069 | 615.85 | 2.68 | 0.000002 | <0.01 |
| Follistatin-like 3 (secreted glycoprotein) | NM_005860 | 203592_s_at | 32.53 | 168.45 | 5.18 | 0.0079 | 0.048 | 404.75 | 12.44 | 0.0048 | <0.01 |
| Forkhead box D1 | NM_004472 | 206307_s_at | 132.29 | 274.90 | 2.08 | 0.031 | 0.069 | 138.81 | 1.05 | 0.84 | 0.32 |
| FtsJ homolog 1 (E. coli) | NM_012280 | 205324_s_at | 78.03 | 151.43 | 1.94 | 0.0031 | 0.41 | 214.52 | 2.75 | 0.018 | <0.01 |
| Galanin prepropeptide | AL556409 | 214240_at | 18.39 | 81.53 | 4.43 | 0.0088 | 0.46 | 254.36 | 13.84 | 0.014 | <0.01 |
| Geminin, DNA replication inhibitor | NM_015895 | 218350_s_at | 62.72 | 148.91 | 2.37 | 0.014 | 0.401 | 203.07 | 3.24 | 0.035 | <0.01 |
| GLI pathogenesis-related 1 | NM_006851 | 204222_s_at | 249.40 | 452.20 | 1.81 | 0.11 | 0.086 | 630.79 | 2.53 | 0.0039 | <0.01 |
| Glutaminase | NM_014905 | 203159_at | 70.13 | 209.34 | 2.98 | 0.0031 | 0.0303 | 159.52 | 2.27 | <0.000001 | 0.097 |
| Glutamine-fructose-6-phosphate transaminase 2 | NM_005110 | 205100_at | 77.67 | 114.17 | 1.47 | 0.081 | 0.55 | 213.49 | 2.75 | 0.0022 | <0.01 |
| Golgi transport 1B | NM_016072 | 218193_s_at | 116.30 | 200.99 | 1.73 | 0.001 | 0.34 | 247.20 | 2.13 | 0.0033 | <0.01 |
| Growth arrest and DNA-damage-inducible, beta | NM_015675 | 207574_s_at | 68.13 | 306.59 | 4.50 | 0.0023 | <0.01 | 351.00 | 5.15 | 0.0023 | <0.01 |
| Growth arrest and DNA-damage-inducible, beta | AF087853 | 209304_x_at | 75.76 | 319.52 | 4.22 | 0.0015 | <0.01 | 328.41 | 4.33 | 0.00094 | <0.01 |
| Growth arrest and DNA-damage-inducible, beta | AF078077 | 209305_s_at | 47.37 | 182.72 | 3.86 | 0.0034 | 0.0303 | 162.38 | 3.43 | 0.0015 | <0.01 |
| H2A histone family, member X | NM_002105 | 205436_s_at | 161.94 | 377.68 | 2.33 | 0.017 | 0.048 | 419.44 | 2.59 | 0.23 | 0.016 |
| Heat shock 27kDa protein 1 | NM_001540 | 201841_s_at | 1003.17 | 1662.64 | 1.66 | 0.13 | 0.069 | 2418.72 | 2.41 | 0.026 | <0.01 |
| Hematological and neurological expressed 1 | NM_016185 | 217755_at | 91.20 | 229.50 | 2.52 | 0.0036 | 0.0303 | 241.10 | 2.64 | 0.059 | <0.01 |
| Hepatoma-derived growth factor, related protein 3 | AB029156 | 209526_s_at | 104.38 | 138.05 | 1.32 | 0.032 | 0.55 | 213.55 | 2.05 | 0.017 | 0.016 |
| Histone cluster 1, H4c | NM_003542 | 205967_at | 121.16 | 333.46 | 2.75 | 0.013 | 0.0303 | 501.59 | 4.14 | 0.049 | <0.01 |
| Hydroxysteroid (17-beta) dehydrogenase 6 homolog (mouse) | NM_003725 | 205700_at | 19.05 | 52.27 | 2.74 | 0.095 | 0.56 | 147.75 | 7.75 | 0.017 | <0.01 |
| Hypoxia up-regulated 1 | NM_006389 | 200825_s_at | 178.85 | 279.21 | 1.56 | 0.024 | 0.069 | 376.58 | 2.11 | 0.000056 | <0.01 |
| Inhibin, beta A | M13436 | 210511_s_at | 143.33 | 688.08 | 4.80 | 0.0012 | <0.01 | 1198.48 | 8.36 | 0.0023 | <0.01 |
| Inhibitor of DNA binding 1, dominant negative helix-loop-helix protein | D13889 | 208937_s_at | 25.45 | 917.52 | 36.05 | 0.00078 | <0.01 | 752.79 | 29.58 | 0.011 | <0.01 |
| Inhibitor of DNA binding 3, dominant negative helix-loop-helix protein | NM_002167 | 207826_s_at | 27.70 | 603.21 | 21.78 | 0.00051 | <0.01 | 603.35 | 21.78 | 0.0025 | <0.01 |
| Insulin-like growth factor binding protein 3 | M31159 | 210095_s_at | 986.38 | 3159.58 | 3.20 | 0.003 | <0.01 | 5241.94 | 5.31 | 0.0051 | <0.01 |
| Insulin-like growth factor binding protein 3 | BF340228 | 212143_s_at | 408.59 | 1489.10 | 3.64 | 0.0068 | <0.01 | 2376.15 | 5.82 | 0.029 | <0.01 |
| Insulin-like growth factor binding protein 7 | NM_001553 | 201162_at | 1174.47 | 1590.26 | 1.35 | 0.34 | 0.15 | 2545.76 | 2.17 | 0.000016 | <0.01 |
| Insulin-like growth factor binding protein 7 | NM_001553 | 201163_s_at | 1374.82 | 1646.20 | 1.20 | 0.49 | 0.22 | 2792.54 | 2.03 | 0.000024 | <0.01 |
| Integrin, alpha E | NM_002208 | 205055_at | 177.23 | 279.60 | 1.58 | 0.0024 | 0.054 | 392.13 | 2.21 | <0.000001 | <0.01 |
| Integrin, beta-like 1 | NM_004791 | 205422_s_at | 138.48 | 175.94 | 1.27 | 0.19 | 0.55 | 303.90 | 2.19 | 0.021 | <0.01 |
| Integrin, beta-like 1 | AL359052 | 214927_at | 71.54 | 137.19 | 1.92 | 0.025 | 0.46 | 229.00 | 3.20 | 0.006 | <0.01 |
|  |  |  |  | **SSc-ILD** | | | | **IPF** | | | |
| **Annotation** | **Accession** | **Probe Set ID** | **Control mean** | **Ssc-ILD mean** | **Fold change** | **p value** | **q-value(%)** | **IPF mean** | **Fold change** | **p value** | **q-value(%)** |
| Interleukin 11 | NM_000641 | 206924_at | 23.62 | 717.79 | 30.39 | 0.015 | <0.01 | 2374.92 | 100.56 | 0.0019 | <0.01 |
| Interleukin 11 | M57765 | 206926_s_at | 36.82 | 117.76 | 3.20 | 0.013 | 0.401 | 322.50 | 8.76 | 0.016 | <0.01 |
| IQ motif containing J-schwannomin interacting protein 1 read-through transcript | NM_014575 | 204030_s_at | 76.86 | 144.58 | 1.88 | 0.015 | 0.46 | 253.98 | 3.30 | 0.00052 | <0.01 |
| Isocitrate dehydrogenase 2 (NADP+), mitochondrial | U52144 | 210046_s_at | 117.64 | 269.41 | 2.29 | 0.019 | 0.054 | 250.54 | 2.13 | 0.0061 | 0.05 |
| KDEL (Lys-Asp-Glu-Leu) endoplasmic reticulum protein retention receptor 3 | NM_016657 | 207265_s_at | 269.26 | 465.69 | 1.73 | 0.0019 | 0.0303 | 631.20 | 2.34 | 0.0024 | <0.01 |
| KIAA0101 | NM_014736 | 202503_s_at | 133.51 | 326.43 | 2.44 | 0.017 | 0.048 | 378.10 | 2.83 | 0.14 | 0.0096 |
| Kruppel-like factor 10 | NM_005655 | 202393_s_at | 131.46 | 245.46 | 1.87 | 0.0049 | 0.054 | 299.80 | 2.28 | 0.032 | 0.0096 |
| Leprecan-like 1 | NM_018192 | 218717_s_at | 33.03 | 103.20 | 3.12 | 0.022 | 0.44 | 225.04 | 6.81 | 0.029 | <0.01 |
| Leucine rich repeat containing 15 | AU147799 | 213909_at | 81.45 | 363.87 | 4.47 | 0.01 | 0.012 | 121.96 | 1.50 | 0.094 | 0.32 |
| Leucine rich repeat containing 17 | NM_005824 | 205381_at | 270.06 | 174.29 | -1.55 | 0.33 | 0.41 | 540.45 | 2.00 | 0.031 | 0.13 |
| Leucine rich repeat containing 59 | AK025328 | 222231_s_at | 306.26 | 571.40 | 1.87 | 0.0055 | <0.01 | 618.40 | 2.02 | 0.0033 | <0.01 |
| Leukemia inhibitory factor (cholinergic differentiation factor) | NM_002309 | 205266_at | 46.52 | 166.79 | 3.59 | 0.0053 | 0.054 | 278.49 | 5.99 | 0.0024 | <0.01 |
| LIM and cysteine-rich domains 1 | NM_014583 | 218574_s_at | 162.68 | 248.49 | 1.53 | 0.11 | 0.46 | 356.24 | 2.19 | 0.0055 | <0.01 |
| LIM and senescent cell antigen-like domains 1 | NM_004987 | 207198_s_at | 194.47 | 313.65 | 1.61 | 0.016 | 0.054 | 447.73 | 2.30 | 0.0045 | <0.01 |
| LIM and senescent cell antigen-like domains 1 | AL110164 | 212687_at | 300.83 | 531.95 | 1.77 | 0.028 | 0.048 | 760.08 | 2.53 | 0.0073 | <0.01 |
| MARCKS-like 1 | NM_023009 | 200644_at | 147.51 | 241.67 | 1.64 | 0.027 | 0.401 | 327.56 | 2.22 | 0.000035 | 0.0096 |
| Maternal embryonic leucine zipper kinase | NM_014791 | 204825_at | 74.13 | 194.32 | 2.62 | 0.023 | 0.054 | 171.04 | 2.31 | 0.19 | 0.17 |
| Matrix Gla protein | NM_000900 | 202291_s_at | 96.22 | 48.88 | -1.97 | 0.33 | 0.41 | 268.39 | 2.79 | 0.022 | 0.073 |
| Melanoma cell adhesion molecule | M28882 | 211340_s_at | 40.12 | 127.39 | 3.18 | 0.042 | 0.41 | 179.69 | 4.48 | 0.039 | <0.01 |
| Met proto-oncogene (hepatocyte growth factor receptor) | BG170541 | 203510_at | 72.74 | 184.74 | 2.54 | 0.034 | 0.069 | 188.79 | 2.60 | 0.0065 | 0.016 |
| Methylenetetrahydrofolate dehydrogenase (NADP+ dependent) 2 | NM_006636 | 201761_at | 256.14 | 392.08 | 1.53 | 0.12 | 0.104 | 591.24 | 2.31 | 0.039 | <0.01 |
| Microtubule associated monoxygenase, calponin and LIM domain containing 2 | BE965029 | 212472_at | 95.31 | 341.04 | 3.58 | 0.0016 | <0.01 | 485.61 | 5.09 | 0.00093 | <0.01 |
| Microtubule associated monoxygenase, calponin and LIM domain containing 2 | BE965029 | 212473_s_at | 184.67 | 667.48 | 3.61 | 0.0039 | <0.01 | 1104.25 | 5.98 | 0.00053 | <0.01 |
| Moesin | NM_002444 | 200600_at | 385.21 | 734.63 | 1.91 | 0.0026 | <0.01 | 859.85 | 2.23 | 0.017 | <0.01 |
| Myeloid/lymphoid or mixed-lineage leukemia (trithorax homolog, Drosophila); translocated to, 11 | BC006471 | 211071_s_at | 90.40 | 223.09 | 2.47 | 0.00001 | <0.01 | 257.33 | 2.85 | 0.028 | <0.01 |
| Myosin light chain kinase | NM_005965 | 202555_s_at | 1055.52 | 1328.18 | 1.26 | 0.64 | 0.22 | 2531.46 | 2.40 | 0.00023 | <0.01 |
| Myosin X | NM_012334 | 201976_s_at | 329.65 | 411.64 | 1.25 | 0.39 | 0.54 | 700.21 | 2.12 | 0.0038 | <0.01 |
| Myosin, light chain 9, regulatory | NM_006097 | 201058_s_at | 417.52 | 670.93 | 1.61 | 0.071 | 0.069 | 1227.80 | 2.94 | 0.018 | <0.01 |
| NADPH oxidase 4 | NM_016931 | 219773_at | 12.26 | 206.59 | 16.86 | 0.012 | 0.0303 | 323.60 | 26.41 | 0.016 | <0.01 |
| Nerve growth factor | NM_002506 | 206814_at | 34.60 | 115.48 | 3.34 | 0.012 | 0.401 | 172.21 | 4.98 | 0.019 | <0.01 |
| NLR family, pyrin domain containing 1 | NM_021730 | 218380_at | 180.29 | 296.85 | 1.65 | 0.25 | 0.15 | 584.10 | 3.24 | 0.00022 | <0.01 |
| Non-metastatic cells 1, protein (NM23A) | NM_000269 | 201577_at | 311.33 | 584.50 | 1.88 | 0.0015 | <0.01 | 687.52 | 2.21 | 0.0074 | <0.01 |
| NUAK family, SNF1-like kinase, 1 | NM_014840 | 204589_at | 56.16 | 232.84 | 4.15 | 0.016 | 0.048 | 489.90 | 8.72 | 0.001 | <0.01 |
| Nucleolar and spindle associated protein 1 | NM_016359 | 218039_at | 67.38 | 182.22 | 2.70 | 0.018 | 0.069 | 205.13 | 3.04 | 0.22 | 0.05 |
| Ornithine decarboxylase 1 | NM_002539 | 200790_at | 105.82 | 280.04 | 2.65 | 0.0072 | 0.0303 | 300.19 | 2.84 | 0.002 | <0.01 |
| Palladin, cytoskeletal associated protein | AK025843 | 200906_s_at | 267.57 | 355.68 | 1.33 | 0.33 | 0.52 | 572.27 | 2.14 | 0.00048 | <0.01 |
| Palladin, cytoskeletal associated protein | AU157932 | 200907_s_at | 624.44 | 834.27 | 1.34 | 0.33 | 0.2 | 1300.79 | 2.08 | 0.00053 | <0.01 |
| PDZ and LIM domain 5 | BG054550 | 203242_s_at | 29.93 | 93.72 | 3.13 | 0.019 | 0.46 | 149.14 | 4.98 | 0.026 | 0.0096 |
| PDZ and LIM domain 5 | NM_006457 | 203243_s_at | 53.22 | 175.79 | 3.30 | 0.0066 | 0.054 | 343.87 | 6.46 | 0.032 | <0.01 |
| PDZ and LIM domain 5 | AK027217 | 216804_s_at | 23.47 | 113.10 | 4.82 | 0.0072 | 0.34 | 184.45 | 7.86 | 0.0059 | <0.01 |
|  |  |  |  | **SSc-ILD** | | | | **IPF** | | | |
| **Annotation** | **Accession** | **Probe Set ID** | **Control mean** | **Ssc-ILD mean** | **Fold change** | **p value** | **q-value(%)** | **IPF mean** | **Fold change** | **p value** | **q-value(%)** |
| PDZ binding kinase | NM_018492 | 219148_at | 36.85 | 147.55 | 4.00 | 0.022 | 0.069 | 149.01 | 4.04 | 0.16 | 0.05 |
| Peroxidasin homolog (Drosophila) | BF342851 | 212012_at | 813.18 | 1088.70 | 1.34 | 0.19 | 0.11 | 1798.50 | 2.21 | 0.00045 | <0.01 |
| Peroxidasin homolog (Drosophila) | D86983 | 212013_at | 246.71 | 358.79 | 1.45 | 0.22 | 0.15 | 587.69 | 2.38 | 0.000015 | <0.01 |
| Phosphofructokinase, platelet | NM_002627 | 201037_at | 175.40 | 529.33 | 3.02 | 0.00033 | <0.01 | 586.05 | 3.34 | 0.00083 | <0.01 |
| Phosphoglucomutase 3 | BC001258 | 210041_s_at | 109.99 | 242.72 | 2.21 | 0.0089 | 0.054 | 291.43 | 2.65 | 0.001 | <0.01 |
| Phosphoglycerate kinase 1 | NM_000291 | 200737_at | 177.79 | 354.74 | 2.00 | 0.0084 | 0.048 | 389.25 | 2.19 | 0.000005 | <0.01 |
| Phosphoglycerate kinase 1 | S81916 | 217356_s_at | 480.76 | 925.05 | 1.92 | 0.0044 | <0.01 | 968.86 | 2.02 | <0.000001 | <0.01 |
| Phospholamban | NM_002667 | 204939_s_at | 25.73 | 170.17 | 6.61 | 0.11 | 0.094 | 460.09 | 17.88 | 0.035 | <0.01 |
| Phospholamban | NM_002667 | 204940_at | 12.99 | 63.03 | 4.85 | 0.099 | 0.54 | 230.40 | 17.73 | 0.029 | <0.01 |
| Phosphoribosyl pyrophosphate synthetase 1 | NM_002764 | 208447_s_at | 58.98 | 283.42 | 4.81 | 0.0036 | <0.01 | 344.99 | 5.85 | 0.037 | <0.01 |
| Phosphoribosyl pyrophosphate synthetase 1 | BC001605 | 209440_at | 141.67 | 548.77 | 3.87 | 0.0021 | <0.01 | 588.03 | 4.15 | 0.0086 | <0.01 |
| Pituitary tumor-transforming 1 | NM_004219 | 203554_x_at | 149.22 | 361.15 | 2.42 | 0.04 | 0.054 | 336.30 | 2.25 | 0.29 | 0.05 |
| Plasminogen activator, urokinase receptor | U08839 | 210845_s_at | 120.27 | 268.46 | 2.23 | 0.0083 | 0.048 | 345.50 | 2.87 | 0.0024 | <0.01 |
| Plasminogen activator, urokinase receptor | AY029180 | 211924_s_at | 69.33 | 149.15 | 2.15 | 0.053 | 0.44 | 200.61 | 2.89 | 0.0068 | <0.01 |
| Pleckstrin homology-like domain, family A, member 2 | AF001294 | 209803_s_at | 292.11 | 417.20 | 1.43 | 0.13 | 0.11 | 602.08 | 2.06 | 0.02 | <0.01 |
| Pleckstrin homology-like domain, family B, member 1 | AB014538 | 212134_at | 91.75 | 119.11 | 1.30 | 0.14 | 0.56 | 200.91 | 2.19 | 0.0079 | 0.016 |
| Potassium large conductance calcium-activated channel, subfamily M, alpha member 1 | U11058 | 221584_s_at | 156.59 | 208.73 | 1.33 | 0.26 | 0.55 | 529.10 | 3.38 | <0.000001 | <0.01 |
| POTE ankyrin domain family, member K, pseudogene | AY014272 | 210926_at | 272.13 | 578.80 | 2.13 | 0.0043 | <0.01 | 746.49 | 2.74 | 0.0053 | <0.01 |
| PRKC, apoptosis, WT1, regulator | AI336206 | 204004_at | 69.24 | 238.13 | 3.44 | 0.0086 | 0.0303 | 340.97 | 4.92 | <0.000001 | <0.01 |
| Procollagen-lysine, 2-oxoglutarate 5-dioxygenase 2 | AI754404 | 202619_s_at | 200.84 | 629.11 | 3.13 | 0.036 | 0.0303 | 775.50 | 3.86 | 0.000001 | <0.01 |
| Procollagen-lysine, 2-oxoglutarate 5-dioxygenase 2 | NM_000935 | 202620_s_at | 144.10 | 414.59 | 2.88 | 0.038 | 0.048 | 696.05 | 4.83 | 0.032 | <0.01 |
| Proenkephalin | NM_006211 | 213791_at | 33.71 | 314.31 | 9.32 | 0.029 | 0.0303 | 366.74 | 10.88 | 0.00086 | <0.01 |
| Prostaglandin-endoperoxide synthase 1 | NM_000962 | 205128_x_at | 122.75 | 405.05 | 3.30 | 0.01 | 0.012 | 898.95 | 7.32 | <0.000001 | <0.01 |
| Prostaglandin-endoperoxide synthase 1 | S36219 | 215813_s_at | 102.39 | 362.03 | 3.54 | 0.0083 | <0.01 | 785.40 | 7.67 | <0.000001 | <0.01 |
| Prostaglandin-endoperoxide synthase 1 | NM_000962 | 205127_at | 48.75 | 141.80 | 2.91 | 0.094 | 0.44 | 281.92 | 5.78 | 0.000006 | <0.01 |
| Proteasome 26S subunit, non-ATPase, 2 | NM_002808 | 200830_at | 322.02 | 680.55 | 2.11 | 0.0033 | <0.01 | 709.61 | 2.20 | 0.059 | <0.01 |
| Protein kinase C, alpha | AI471375 | 213093_at | 184.82 | 234.83 | 1.27 | 0.18 | 0.55 | 388.47 | 2.10 | 0.00085 | <0.01 |
| Protein phosphatase 1, regulatory (inhibitor) subunit 14B | BE305165 | 212680_x_at | 103.57 | 229.14 | 2.21 | 0.035 | 0.069 | 184.04 | 1.78 | 0.025 | 0.29 |
| Protein regulator of cytokinesis 1 | NM_003981 | 218009_s_at | 41.41 | 165.05 | 3.99 | 0.015 | 0.054 | 139.52 | 3.37 | 0.24 | 0.32 |
| Protein tyrosine phosphatase-like, member A | NM_014241 | 219654_at | 84.73 | 144.20 | 1.70 | 0.016 | 0.504 | 216.85 | 2.56 | 0.038 | 0.0096 |
| Proteolipid protein 2 (colonic epithelium-enriched) | NM_002668 | 201136_at | 233.32 | 439.60 | 1.88 | 0.0026 | <0.01 | 670.20 | 2.87 | 0.021 | <0.01 |
| Pyruvate dehydrogenase (lipoamide) beta | AL117618 | 211023_at | 244.95 | 331.60 | 1.35 | 0.044 | 0.41 | 537.45 | 2.19 | 0.0033 | <0.01 |
| RAB3B, member RAS oncogene family | BC005035 | 205924_at | 76.96 | 205.42 | 2.67 | 0.011 | 0.054 | 329.06 | 4.28 | 0.032 | <0.01 |
| RAB3B, member RAS oncogene family | NM_002867 | 205925_s_at | 48.79 | 115.81 | 2.37 | 0.011 | 0.46 | 215.15 | 4.41 | 0.017 | <0.01 |
| Ras homolog gene family, member B | AI263909 | 212099_at | 86.54 | 303.60 | 3.51 | 0.00099 | <0.01 | 405.14 | 4.68 | 0.00055 | <0.01 |
| Regulator of G-protein signaling 4 | AL514445 | 204337_at | 65.89 | 120.18 | 1.82 | 0.19 | 0.54 | 204.44 | 3.10 | 0.012 | 0.0096 |
| Rho guanine nucleotide exchange factor (GEF) 40 | R42449 | 58780_s_at | 138.43 | 177.84 | 1.28 | 0.19 | 0.55 | 314.49 | 2.27 | 0.0094 | <0.01 |
| Ribonucleotide reductase M1 | AI692974 | 201476_s_at | 103.53 | 214.12 | 2.07 | 0.023 | 0.069 | 221.32 | 2.14 | 0.16 | 0.05 |
| Ribonucleotide reductase M1 | NM_001033 | 201477_s_at | 102.65 | 216.19 | 2.11 | 0.015 | 0.054 | 200.49 | 1.95 | 0.19 | 0.17 |
| Ribonucleotide reductase M2 | BE966236 | 201890_at | 56.12 | 197.08 | 3.51 | 0.02 | 0.054 | 266.63 | 4.75 | 0.14 | <0.01 |
| Ribonucleotide reductase M2 | BC001886 | 209773_s_at | 103.33 | 353.46 | 3.42 | 0.021 | 0.0303 | 353.58 | 3.42 | 0.23 | 0.016 |
| Ribosomal protein S4, Y-linked 1 | NM_001008 | 201909_at | 343.28 | 469.48 | 1.37 | 0.44 | 0.22 | 1117.57 | 3.26 | 0.000001 | <0.01 |
| RING1 and YY1 binding protein | W84482 | 201844_s_at | 99.21 | 144.60 | 1.46 | 0.12 | 0.54 | 218.25 | 2.20 | 0.000015 | 0.0096 |
|  |  |  |  | **SSc-ILD** | | | | **IPF** | | | |
| **Annotation** | **Accession** | **Probe Set ID** | **Control mean** | **Ssc-ILD mean** | **Fold change** | **p value** | **q-value(%)** | **IPF mean** | **Fold change** | **p value** | **q-value(%)** |
| Runt-related transcription factor 1 | D43968 | 209360_s_at | 113.55 | 198.56 | 1.75 | 0.01 | 0.401 | 239.73 | 2.11 | 0.00001 | 0.028 |
| Secreted protein, acidic, cysteine-rich (osteonectin) | AL575922 | 212667_at | 878.92 | 845.52 | -1.04 | 0.87 | 0.41 | 1899.52 | 2.16 | 0.00001 | <0.01 |
| Serine/threonine kinase 38 like | AW779556 | 212572_at | 61.20 | 147.28 | 2.41 | 0.012 | 0.34 | 282.18 | 4.61 | 0.024 | <0.01 |
| Serpin peptidase inhibitor, clade E, member 1 | AL574210 | 202627_s_at | 373.34 | 2453.13 | 6.57 | 0.004 | <0.01 | 2929.30 | 7.85 | 0.01 | <0.01 |
| Serpin peptidase inhibitor, clade E, member 1 | NM_000602 | 202628_s_at | 329.82 | 2473.18 | 7.50 | 0.0022 | <0.01 | 2662.64 | 8.07 | 0.019 | <0.01 |
| SET and MYND domain containing 3 | NM_022743 | 218788_s_at | 74.68 | 179.78 | 2.41 | 0.019 | 0.069 | 224.39 | 3.00 | 0.057 | <0.01 |
| SH3 and PX domains 2A | AI739005 | 213252_at | 54.19 | 120.18 | 2.22 | 0.0062 | 0.44 | 217.57 | 4.01 | 0.000001 | <0.01 |
| Smoothelin | NM_006932 | 207390_s_at | 46.47 | 147.71 | 3.18 | 0.0047 | 0.054 | 161.79 | 3.48 | 0.0014 | <0.01 |
| Smoothelin | AF064238 | 209427_at | 51.64 | 154.70 | 3.00 | 0.0056 | 0.054 | 133.27 | 2.58 | 0.04 | 0.097 |
| Solute carrier family 16, member 2 | NM_006517 | 204462_s_at | 54.07 | 118.90 | 2.20 | 0.0059 | 0.46 | 185.51 | 3.43 | <0.000001 | <0.01 |
| Solute carrier family 25, member 32 | NM_030780 | 221020_s_at | 74.78 | 154.88 | 2.07 | 0.011 | 0.401 | 188.85 | 2.53 | 0.0079 | 0.0096 |
| Solute carrier family 25, member 4 | NM_001151 | 202825_at | 57.05 | 105.50 | 1.85 | 0.12 | 0.54 | 163.35 | 2.86 | 0.012 | 0.016 |
| Solute carrier family 7, member 1 | AA148507 | 212295_s_at | 171.25 | 352.68 | 2.06 | 0.014 | 0.048 | 310.04 | 1.81 | 0.036 | 0.05 |
| Solute carrier family 7, member 5 | AB018009 | 201195_s_at | 56.08 | 294.19 | 5.25 | 0.00022 | <0.01 | 199.93 | 3.56 | 0.025 | <0.01 |
| Sparc/osteonectin, cwcv and kazal-like domains proteoglycan (testican) 1 | AF231124 | 202363_at | 587.91 | 678.57 | 1.15 | 0.47 | 0.54 | 1417.80 | 2.41 | 0.0071 | <0.01 |
| Spectrin repeat containing, nuclear envelope 1 | AF043290 | 209447_at | 86.22 | 162.42 | 1.88 | 0.053 | 0.46 | 231.20 | 2.68 | 0.0027 | <0.01 |
| Spermidine synthase | NM_003132 | 201516_at | 116.04 | 236.03 | 2.03 | 0.00034 | 0.0303 | 320.62 | 2.76 | 0.021 | <0.01 |
| SRY (sex determining region Y)-box 4 | BG528420 | 201416_at | 76.08 | 101.27 | 1.33 | 0.25 | 0.56 | 215.21 | 2.83 | 0.000024 | 0.016 |
| Sushi-repeat-containing protein, X-linked 2 | NM_014467 | 205499_at | 114.84 | 125.73 | 1.09 | 0.69 | 0.56 | 243.55 | 2.12 | 0.000003 | <0.01 |
| Synaptojanin 2 | AF318616 | 210612_s_at | 114.99 | 193.86 | 1.69 | 0.042 | 0.44 | 250.76 | 2.18 | 0.0055 | 0.016 |
| Syndecan 1 | Z48199 | 201286_at | 36.50 | 144.36 | 3.95 | 0.021 | 0.054 | 131.25 | 3.60 | 0.12 | 0.097 |
| TCDD-inducible poly(ADP-ribose) polymerase | AL556438 | 212665_at | 147.41 | 326.67 | 2.22 | 0.00071 | 0.012 | 206.28 | 1.40 | 0.23 | 0.32 |
| Tensin 1 | AF116610 | 218864_at | 27.88 | 67.82 | 2.43 | 0.0039 | 0.54 | 138.00 | 4.95 | 0.0027 | <0.01 |
| Tensin 1 | NM_018274 | 221246_x_at | 211.87 | 320.59 | 1.51 | 0.056 | 0.094 | 702.52 | 3.32 | 0.0036 | <0.01 |
| Tensin 1 | AL046979 | 221747_at | 81.00 | 162.74 | 2.01 | 0.019 | 0.401 | 321.05 | 3.96 | 0.0056 | <0.01 |
| Tensin 1 | AL046979 | 221748_s_at | 232.17 | 380.64 | 1.64 | 0.041 | 0.069 | 832.15 | 3.58 | 0.0089 | <0.01 |
| Tetraspanin 13 | NM_014399 | 217979_at | 37.92 | 533.94 | 14.08 | 0.0033 | <0.01 | 1039.09 | 27.40 | 0.0052 | <0.01 |
| Thioredoxin-like 4A | NM_006701 | 202836_s_at | 316.13 | 476.84 | 1.51 | 0.045 | 0.069 | 682.64 | 2.16 | <0.000001 | <0.01 |
| TIMP metallopeptidase inhibitor 3 | AW338933 | 201148_s_at | 332.05 | 567.82 | 1.71 | 0.16 | 0.1 | 691.91 | 2.08 | 0.00039 | <0.01 |
| TIMP metallopeptidase inhibitor 3 | U67195 | 201149_s_at | 448.76 | 834.11 | 1.86 | 0.13 | 0.069 | 925.83 | 2.06 | 0.027 | <0.01 |
| Tissue factor pathway inhibitor 2 | AL574096 | 209277_at | 278.00 | 468.40 | 1.68 | 0.13 | 0.11 | 721.37 | 2.59 | 0.0032 | <0.01 |
| Tissue factor pathway inhibitor 2 | L27624 | 209278_s_at | 501.16 | 816.93 | 1.63 | 0.2 | 0.13 | 1347.78 | 2.69 | 0.00038 | <0.01 |
| Topoisomerase (DNA) II alpha 170kDa | AU159942 | 201291_s_at | 55.26 | 219.87 | 3.98 | 0.018 | 0.054 | 239.96 | 4.34 | 0.19 | 0.016 |
| Topoisomerase (DNA) II alpha 170kDa | AL561834 | 201292_at | 44.15 | 190.98 | 4.33 | 0.0037 | 0.0303 | 230.58 | 5.22 | 0.16 | 0.0096 |
| TPX2, microtubule-associated, homolog (Xenopus laevis) | AF098158 | 210052_s_at | 41.86 | 163.78 | 3.91 | 0.018 | 0.054 | 118.36 | 2.83 | 0.33 | 0.29 |
| Transcription factor 4 | AK026674 | 222146_s_at | 116.49 | 166.45 | 1.43 | 0.17 | 0.54 | 251.25 | 2.16 | 0.000068 | 0.028 |
| Transforming growth factor, beta 1 | BC000125 | 203085_s_at | 77.29 | 175.85 | 2.28 | 0.045 | 0.401 | 281.22 | 3.64 | <0.000001 | <0.01 |
| Transgelin | NM_003186 | 205547_s_at | 1004.01 | 2054.46 | 2.05 | 0.026 | 0.0303 | 3521.21 | 3.51 | <0.000001 | <0.01 |
| Transmembrane protein 2 | NM_013390 | 218113_at | 94.73 | 283.11 | 2.99 | 0.046 | 0.054 | 379.36 | 4.00 | 0.0042 | <0.01 |
| Tribbles homolog 1 (Drosophila) | NM_025195 | 202241_at | 63.07 | 104.88 | 1.66 | 0.031 | 0.54 | 172.48 | 2.73 | 0.033 | 0.0096 |
| Triosephosphate isomerase 1 | BF116254 | 213011_s_at | 591.91 | 1024.39 | 1.73 | 0.00009 | <0.01 | 1245.41 | 2.10 | 0.0022 | <0.01 |
| Tropomyosin 1 (alpha) | NM_000366 | 206116_s_at | 317.53 | 1619.64 | 5.10 | 0.0032 | <0.01 | 2552.80 | 8.04 | 0.00019 | <0.01 |
| Tropomyosin 1 (alpha) | NM_000366 | 206117_at | 30.90 | 186.96 | 6.05 | 0.0033 | 0.022 | 261.73 | 8.47 | 0.0069 | <0.01 |
|  |  |  |  | **SSc-ILD** | | | | **IPF** | | | |
| **Annotation** | **Accession** | **Probe Set ID** | **Control mean** | **Ssc-ILD mean** | **Fold change** | **p value** | **q-value(%)** | **IPF mean** | **Fold change** | **p value** | **q-value(%)** |
| Tropomyosin 1 (alpha) | Z24727 | 210986_s_at | 756.33 | 2438.52 | 3.22 | 0.0046 | <0.01 | 3881.04 | 5.13 | 0.00099 | <0.01 |
| Tropomyosin 1 (alpha) | M19267 | 210987_x_at | 621.30 | 2528.40 | 4.07 | 0.0025 | <0.01 | 3465.92 | 5.58 | <0.000001 | <0.01 |
| Tubulin, alpha 4a | AL565074 | 212242_at | 106.82 | 203.83 | 1.91 | 0.016 | 0.34 | 284.77 | 2.67 | 0.0041 | <0.01 |
| Tubulin, beta 2A | NM_001069 | 204141_at | 181.52 | 361.92 | 1.99 | 0.0059 | 0.0303 | 436.68 | 2.41 | 0.0011 | <0.01 |
| Tubulin, beta 2A | BF971587 | 209372_x_at | 72.01 | 188.83 | 2.62 | 0.0022 | 0.0303 | 189.22 | 2.63 | 0.0056 | <0.01 |
| Tubulin, beta 3 | NM_006086 | 202154_x_at | 449.19 | 968.49 | 2.16 | 0.0083 | <0.01 | 779.26 | 1.73 | 0.059 | <0.01 |
| Tubulin, beta 3 | AL565749 | 213476_x_at | 420.77 | 952.29 | 2.26 | 0.0023 | <0.01 | 820.50 | 1.95 | 0.04 | <0.01 |
| Tubulin, beta 6 | BC002654 | 209191_at | 588.66 | 1204.43 | 2.05 | 0.0075 | <0.01 | 1250.96 | 2.13 | 0.019 | <0.01 |
| Tumor necrosis factor receptor superfamily, member 12A | NM_016639 | 218368_s_at | 175.59 | 440.75 | 2.51 | 0.016 | 0.022 | 548.95 | 3.13 | 0.0034 | <0.01 |
| Ubiquitin-conjugating enzyme E2C | NM_007019 | 202954_at | 92.01 | 291.33 | 3.17 | 0.014 | 0.0303 | 294.10 | 3.20 | 0.22 | 0.016 |
| Ubiquitin-conjugating enzyme E2S | NM_014501 | 202779_s_at | 106.80 | 377.09 | 3.53 | 0.0087 | <0.01 | 457.59 | 4.28 | 0.041 | <0.01 |
| UDP-N-acetyl-alpha-D-galactosamine | BE906572 | 212256_at | 54.51 | 171.76 | 3.15 | 0.0018 | 0.0303 | 242.97 | 4.46 | <0.000001 | <0.01 |
| UDP-N-acetyl-alpha-D-galactosamine:polypeptide N-acetylgalactosaminyltransferase 10 | NM_017540 | 207357_s_at | 36.17 | 111.39 | 3.08 | 0.0018 | 0.401 | 163.92 | 4.53 | 0.0015 | <0.01 |
| Uridine-cytidine kinase 2 | BC002906 | 209825_s_at | 79.65 | 229.59 | 2.88 | 0.0049 | 0.0303 | 290.33 | 3.65 | 0.000002 | <0.01 |
| Vascular endothelial growth factor A | AF022375 | 210512_s_at | 173.63 | 337.19 | 1.94 | 0.00084 | 0.012 | 458.96 | 2.64 | 0.019 | <0.01 |
| Versican | BF590263 | 204619_s_at | 128.19 | 273.34 | 2.13 | 0.019 | 0.054 | 568.69 | 4.44 | 0.023 | <0.01 |
| Versican | NM_004385 | 204620_s_at | 353.15 | 891.88 | 2.53 | 0.0015 | <0.01 | 1456.46 | 4.12 | 0.000087 | <0.01 |
| Versican | D32039 | 211571_s_at | 164.13 | 441.26 | 2.69 | 0.00068 | <0.01 | 735.97 | 4.48 | 0.000027 | <0.01 |
| Versican | R94644 | 215646_s_at | 141.85 | 353.27 | 2.49 | 0.002 | 0.012 | 827.52 | 5.83 | 0.00064 | <0.01 |
| Versican | BF218922 | 221731_x_at | 351.23 | 846.90 | 2.41 | 0.00059 | <0.01 | 1402.44 | 3.99 | 0.0019 | <0.01 |
| Vitamin D receptor | NM_000376 | 204254_s_at | 101.98 | 151.56 | 1.49 | 0.18 | 0.54 | 245.01 | 2.40 | 0.00034 | <0.01 |
| WAP four-disulfide core domain 1 | NM_021197 | 219478_at | 150.66 | 297.65 | 1.98 | 0.31 | 0.2 | 389.04 | 2.58 | 0.0045 | 0.05 |
| Wingless-type MMTV integration site family, member 5A | AI968085 | 213425_at | 345.24 | 552.51 | 1.60 | 0.15 | 0.11 | 726.85 | 2.11 | 0.0079 | 0.0096 |
| WW domain containing transcription regulator 1 | BF674349 | 202133_at | 312.03 | 395.27 | 1.27 | 0.30 | 0.51732678 | 678.88 | 2.18 | 0.00012 | <0.01 |
| Xylosyltransferase I | AI693140 | 213725_x_at | 29.01 | 255.42 | 8.80 | 0.0024 | <0.01 | 443.62 | 15.29 | 0.034 | <0.01 |
| Yip1 domain family, member 5 | NM_030799 | 221423_s_at | 220.59 | 302.86 | 1.37 | 0.081 | 0.46 | 453.18 | 2.05 | 0.0028 | <0.01 |
| ZW10 interactor | NM_007057 | 204026_s_at | 53.65 | 187.41 | 3.49 | 0.012 | 0.054 | 183.56 | 3.42 | 0.17 | 0.028 |
| **Underexpressed in SSc-ILD and/or IPF** | | | | | | | | | | | |
| --- | AL121994 | 216565_x_at | 684.40 | 192.38 | -3.56 | 0.00003 | <0.01 | 141.13 | -4.85 | 0.000022 | <0.01 |
| 2',5'-oligoadenylate synthetase 1, 40/46kDa | NM_016816 | 202869_at | 482.74 | 26.56 | -18.18 | 0.0018 | <0.01 | 13.34 | -36.19 | 0.0015 | 0.028 |
| 2',5'-oligoadenylate synthetase 1, 40/46kDa | NM_002534 | 205552_s_at | 374.61 | 18.32 | -20.45 | 0.0014 | <0.01 | 5.48 | -68.32 | 0.0012 | 0.028 |
| 2'-5'-oligoadenylate synthetase 2, 69/71kDa | NM_016817 | 204972_at | 279.23 | 27.91 | -10.00 | 0.00038 | <0.01 | 6.55 | -42.61 | 0.00025 | 0.016 |
| 2'-5'-oligoadenylate synthetase 2, 69/71kDa | NM_002535 | 206553_at | 117.22 | 17.12 | -6.85 | 0.00087 | <0.01 | 10.67 | -10.99 | 0.00059 | 0.073 |
| 2'-5'-oligoadenylate synthetase 3, 100kDa | NM_006187 | 218400_at | 305.50 | 53.43 | -5.72 | 0.00015 | <0.01 | 42.31 | -7.22 | 0.00013 | 0.016 |
| 2'-5'-oligoadenylate synthetase-like | NM_003733 | 205660_at | 246.36 | 21.63 | -11.39 | 0.019 | <0.01 | 12.56 | -19.61 | 0.016 | 0.13 |
| 2'-5'-oligoadenylate synthetase-like | AF063612 | 210797_s_at | 249.07 | 33.74 | -7.38 | 0.044 | 0.022 | 36.52 | -6.82 | 0.046 | 0.22 |
| 5'-nucleotidase, ecto (CD73) | NM_002526 | 203939_at | 991.18 | 599.91 | -1.65 | 0.0022 | <0.01 | 294.12 | -3.37 | 0.000013 | <0.01 |
| A kinase (PRKA) anchor protein 2 | BE879367 | 202759_s_at | 526.74 | 287.69 | -1.83 | 0.00017 | <0.01 | 179.11 | -2.94 | 0.000004 | <0.01 |
| A kinase (PRKA) anchor protein 2 | NM_007203 | 202760_s_at | 470.57 | 240.37 | -1.96 | 0.00048 | <0.01 | 134.34 | -3.50 | 0.000027 | <0.01 |
| Acyl-CoA synthetase long-chain family member 3 | D89053 | 201662_s_at | 286.97 | 138.73 | -2.07 | 0.0011 | <0.01 | 150.07 | -1.91 | 0.012 | 0.073 |
| ADAM metallopeptidase with thrombospondin type 1 motif, 1 | AK023795 | 222162_s_at | 512.33 | 286.91 | -1.79 | 0.26 | 0.22 | 78.76 | -6.50 | 0.0057 | 0.049 |
| Adducin 3 (gamma) | BE545756 | 201034_at | 520.40 | 243.82 | -2.13 | 0.0041 | <0.01 | 180.36 | -2.89 | 0.0012 | 0.028 |
| Adducin 3 (gamma) | AI763123 | 201752_s_at | 469.13 | 218.24 | -2.15 | 0.00078 | <0.01 | 148.33 | -3.16 | 0.000083 | 0.0096 |
|  |  |  |  | **SSc-ILD** | | | | **IPF** | | | |
| **Annotation** | **Accession** | **Probe Set ID** | **Control mean** | **Ssc-ILD mean** | **Fold change** | **p value** | **q-value(%)** | **IPF mean** | **Fold change** | **p value** | **q-value(%)** |
| Adducin 3 (gamma) | NM_019903 | 201753_s_at | 376.81 | 160.14 | -2.35 | 0.0049 | <0.01 | 126.67 | -2.97 | 0.0022 | 0.049 |
| Adducin 3 (gamma) | AI818488 | 205882_x_at | 445.76 | 203.48 | -2.19 | 0.0014 | <0.01 | 139.49 | -3.20 | 0.00025 | 0.016 |
| Adenosine deaminase, RNA-specific | NM_001111 | 201786_s_at | 861.16 | 346.09 | -2.49 | 0.000002 | <0.01 | 291.76 | -2.95 | 0.000002 | <0.01 |
| Adenosine monophosphate deaminase 3 | NM_000480 | 207992_s_at | 138.40 | 35.87 | -3.86 | 0.00022 | <0.01 | 22.91 | -6.04 | 0.0001 | 0.049 |
| Adrenomedullin | NM_001124 | 202912_at | 1968.38 | 421.93 | -4.67 | 0.000011 | <0.01 | 130.19 | -15.12 | 0.000006 | <0.01 |
| Alcohol dehydrogenase 1B (class I), beta polypeptide | M24317 | 209612_s_at | 606.08 | 54.45 | -11.13 | 0.012 | <0.01 | 17.25 | -35.14 | 0.0084 | 0.049 |
| Alcohol dehydrogenase 1B (class I), beta polypeptide | M21692 | 209613_s_at | 268.94 | 19.82 | -13.57 | 0.011 | <0.01 | 6.37 | -42.20 | 0.008 | 0.073 |
| Alcohol dehydrogenase 1B (class I), beta polypeptide | AF153821 | 209614_at | 110.38 | 7.41 | -14.89 | 0.011 | 0.022 | 3.42 | -32.31 | 0.009 | 0.13 |
| Alcohol dehydrogenase 5 (class III), chi polypeptide | M30471 | 208848_at | 260.04 | 124.11 | -2.10 | 0.000011 | <0.01 | 173.82 | -1.50 | 0.099 | 0.17 |
| Aldehyde dehydrogenase 1 family, member A3 | NM_000693 | 203180_at | 111.72 | 17.52 | -6.38 | 0.00028 | 0.14 | 6.08 | -18.38 | 0.00013 | 0.073 |
| Aldehyde dehydrogenase 2 family (mitochondrial) | NM_000690 | 201425_at | 396.35 | 195.53 | -2.03 | 0.047 | 0.022 | 158.71 | -2.50 | 0.017 | 0.13 |
| Aldo-keto reductase family 1, member B1 | NM_001628 | 201272_at | 2024.30 | 789.86 | -2.56 | 0.00048 | <0.01 | 305.32 | -6.63 | 0.000037 | <0.01 |
| Aldo-keto reductase family 1, member C1 | NM_001353 | 204151_x_at | 1649.18 | 457.13 | -3.61 | 0.000093 | <0.01 | 216.00 | -7.64 | 0.000021 | <0.01 |
| Aldo-keto reductase family 1, member C1 | S68290 | 216594_x_at | 1136.23 | 335.13 | -3.39 | 0.00006 | <0.01 | 150.78 | -7.54 | 0.000006 | <0.01 |
| Aldo-keto reductase family 1, member C2 | U05598 | 209699_x_at | 1413.06 | 387.06 | -3.65 | 0.000095 | <0.01 | 188.86 | -7.48 | 0.000025 | <0.01 |
| Aldo-keto reductase family 1, member C2 | M33376 | 211653_x_at | 1100.18 | 338.61 | -3.25 | 0.00005 | <0.01 | 170.64 | -6.45 | 0.000001 | <0.01 |
| Aldo-keto reductase family 1, member C3 | AB018580 | 209160_at | 247.34 | 76.30 | -3.24 | 0.00014 | <0.01 | 37.66 | -6.57 | 0.000011 | 0.0096 |
| Amyloid beta (A4) precursor protein | X06989 | 214953_s_at | 516.18 | 341.92 | -1.51 | 0.29 | 0.048 | 209.13 | -2.47 | 0.033 | 0.13 |
| Amyloid beta (A4) precursor-like protein 2 | NM_001642 | 208248_x_at | 647.73 | 423.50 | -1.53 | 0.024 | 0.012 | 300.17 | -2.16 | 0.0015 | 0.028 |
| Amyloid beta (A4) precursor-like protein 2 | BG427393 | 208703_s_at | 346.76 | 240.68 | -1.44 | 0.034 | 0.0303 | 151.69 | -2.29 | 0.00046 | 0.049 |
| Amyloid beta (A4) precursor-like protein 2 | BC000373 | 208704_x_at | 749.41 | 469.54 | -1.60 | 0.025 | <0.01 | 343.44 | -2.18 | 0.0024 | 0.028 |
| Amyloid beta (A4) precursor-like protein 2 | BG427393 | 208890_s_at | 246.92 | 168.76 | -1.46 | 0.021 | 0.41 | 103.92 | -2.38 | 0.000001 | 0.0096 |
| Amyloid beta (A4) precursor-like protein 2 | AW001847 | 214875_x_at | 321.55 | 220.94 | -1.46 | 0.028 | 0.0303 | 151.57 | -2.12 | 0.00086 | 0.049 |
| Angiopoietin-like 2 | AF007150 | 213001_at | 590.96 | 405.79 | -1.46 | 0.29 | 0.048 | 215.58 | -2.74 | 0.008 | 0.049 |
| Annexin A4 | BC000182 | 201301_s_at | 314.15 | 185.18 | -1.70 | 0.00093 | <0.01 | 146.82 | -2.14 | 0.00093 | 0.016 |
| Annexin A4 | NM_001153 | 201302_at | 198.58 | 120.52 | -1.65 | 0.00087 | 0.18 | 96.12 | -2.07 | 0.0042 | 0.049 |
| Apolipoprotein L, 1 | AF323540 | 209546_s_at | 139.58 | 15.50 | -9.00 | 0.00024 | <0.01 | 13.51 | -10.33 | 0.00021 | 0.049 |
| Apolipoprotein L, 3 | NM_014349 | 221087_s_at | 124.85 | 13.52 | -9.23 | 0.00024 | <0.01 | 10.46 | -11.94 | 0.00019 | 0.073 |
| Argininosuccinate synthase 1 | NM_000050 | 207076_s_at | 321.27 | 239.38 | -1.34 | 0.14 | 0.401 | 107.34 | -2.99 | 0.0004 | 0.028 |
| Armadillo repeat containing, X-linked 1 | NM_016608 | 218694_at | 283.89 | 149.93 | -1.89 | 0.0037 | <0.01 | 98.92 | -2.87 | 0.00015 | 0.028 |
| Aryl hydrocarbon receptor | NM_001621 | 202820_at | 216.93 | 99.16 | -2.19 | 0.00051 | <0.01 | 62.50 | -3.47 | 0.000081 | 0.028 |
| AT rich interactive domain 5B (MRF1-like) | BG285011 | 212614_at | 1078.55 | 384.02 | -2.81 | 0.00019 | <0.01 | 612.69 | -1.76 | 0.0035 | 0.05 |
| ATPase, Ca++ transporting, plasma membrane 1 | M95541 | 209281_s_at | 245.17 | 128.27 | -1.91 | 0.001 | <0.01 | 75.05 | -3.27 | 0.00007 | 0.016 |
| ATPase, Ca++ transporting, plasma membrane 1 | L14561 | 215716_s_at | 201.82 | 101.24 | -1.99 | 0.0025 | 0.012 | 52.02 | -3.88 | 0.000019 | 0.028 |
| ATPase, H+ transporting, lysosomal 56/58kDa, V1 subunit B2 | NM_001693 | 201089_at | 218.01 | 180.17 | -1.21 | 0.43 | 0.41 | 103.07 | -2.12 | 0.0025 | 0.073 |
| ATP-binding cassette, sub-family A (ABC1), member 1 | NM_005502 | 203504_s_at | 339.68 | 126.53 | -2.68 | 0.000011 | <0.01 | 106.51 | -3.19 | 0.000005 | <0.01 |
| ATP-binding cassette, sub-family A (ABC1), member 1 | AF285167 | 203505_at | 278.42 | 101.37 | -2.75 | 0.00097 | <0.01 | 115.53 | -2.41 | 0.0035 | 0.073 |
| B-cell CLL/lymphoma 6 | NM_001706 | 203140_at | 190.40 | 80.55 | -2.36 | 0.00016 | <0.01 | 73.40 | -2.59 | 0.00044 | 0.073 |
| B-cell translocation gene 1, anti-proliferative | AL535380 | 200920_s_at | 622.30 | 387.10 | -1.61 | 0.045 | <0.01 | 208.46 | -2.99 | 0.000029 | <0.01 |
| B-cell translocation gene 1, anti-proliferative | NM_001731 | 200921_s_at | 333.25 | 203.58 | -1.64 | 0.03 | 0.022 | 114.05 | -2.92 | 0.00037 | 0.0096 |
| BCL2 binding component 3 | AF332558 | 211692_s_at | 168.79 | 88.06 | -1.92 | 0.0025 | 0.22 | 65.87 | -2.56 | 0.000049 | 0.073 |
| Beta-2-microglobulin | NM_004048 | 201891_s_at | 4244.60 | 2483.68 | -1.71 | 0.000003 | <0.01 | 1962.75 | -2.16 | <0.000001 | <0.01 |
| Beta-2-microglobulin | AW188940 | 216231_s_at | 6163.90 | 3605.98 | -1.71 | 0.000056 | <0.01 | 2833.03 | -2.18 | 0.000002 | <0.01 |
| Bone marrow stromal cell antigen 2 | NM_004335 | 201641_at | 315.76 | 12.49 | -25.27 | 0.0081 | <0.01 | 6.24 | -50.57 | 0.0072 | 0.073 |
|  |  |  |  | **SSc-ILD** | | | | **IPF** | | | |
| **Annotation** | **Accession** | **Probe Set ID** | **Control mean** | **Ssc-ILD mean** | **Fold change** | **p value** | **q-value(%)** | **IPF mean** | **Fold change** | **p value** | **q-value(%)** |
| Bradykinin receptor B1 | NM_000710 | 207510_at | 524.20 | 155.00 | -3.38 | 0.0012 | <0.01 | 311.36 | -1.68 | 0.027 | 0.13 |
| Bradykinin receptor B2 | NM_000623 | 205870_at | 407.71 | 109.24 | -3.73 | 0.000001 | <0.01 | 130.65 | -3.12 | 0.000003 | <0.01 |
| Butyrophilin, subfamily 3, member A2 | NM_006994 | 204820_s_at | 371.47 | 91.52 | -4.06 | 0.00063 | <0.01 | 95.01 | -3.91 | 0.00072 | 0.028 |
| Calcium/calmodulin-dependent protein kinase II inhibitor 1 | NM_018584 | 218309_at | 714.76 | 278.64 | -2.57 | 0.00039 | <0.01 | 404.23 | -1.77 | 0.0045 | 0.05 |
| Carbonic anhydrase XII | NM_001218 | 203963_at | 216.85 | 123.17 | -1.76 | 0.11 | 0.34 | 32.31 | -6.71 | 0.0036 | 0.073 |
| Carbonic anhydrase XII | BF752277 | 214164_x_at | 311.72 | 157.58 | -1.98 | 0.047 | 0.03 | 21.60 | -14.43 | 0.0012 | 0.028 |
| Carbonic anhydrase XII | AL050025 | 215867_x_at | 300.99 | 157.95 | -1.91 | 0.041 | 0.03 | 19.25 | -15.64 | 0.00068 | 0.028 |
| CASP8 and FADD-like apoptosis regulator | AF009616 | 211316_x_at | 227.40 | 112.45 | -2.02 | 0.0073 | 0.012 | 120.34 | -1.89 | 0.0092 | 0.13 |
| Caspase 1 (interleukin 1, beta, convertase) | M87507 | 209970_x_at | 331.85 | 80.61 | -4.12 | 0.000001 | <0.01 | 52.95 | -6.27 | 0.000001 | <0.01 |
| Caspase 1 (interleukin 1, beta, convertase) | U13698 | 211366_x_at | 364.20 | 88.39 | -4.12 | <0.000001 | <0.01 | 49.18 | -7.41 | <0.000001 | <0.01 |
| Caspase 1 (interleukin 1, beta, convertase) | U13699 | 211367_s_at | 180.59 | 35.05 | -5.15 | <0.000001 | <0.01 | 9.99 | -18.08 | <0.000001 | <0.01 |
| Caspase 1 (interleukin 1, beta, convertase) | U13700 | 211368_s_at | 154.32 | 29.61 | -5.21 | 0.000002 | <0.01 | 15.77 | -9.79 | 0.000001 | 0.016 |
| Caspase 4, apoptosis-related cysteine peptidase | U25804 | 209310_s_at | 177.10 | 96.23 | -1.84 | 0.008 | 0.26 | 76.98 | -2.30 | 0.0018 | 0.13 |
| Cathepsin A | NM_000308 | 200661_at | 746.21 | 435.09 | -1.72 | 0.00202 | <0.01 | 365.58 | -2.04 | 0.00029 | 0.0096 |
| Cathepsin B | NM_001908 | 200838_at | 1294.18 | 638.23 | -2.03 | 0.0053 | <0.01 | 504.10 | -2.57 | 0.0011 | 0.016 |
| Cathepsin B | NM_001908 | 200839_s_at | 2266.15 | 1298.91 | -1.74 | 0.0057 | <0.01 | 1088.52 | -2.08 | 0.00087 | 0.0096 |
| Cathepsin C | NM_001814 | 201487_at | 550.75 | 194.65 | -2.83 | 0.00015 | <0.01 | 210.26 | -2.62 | 0.00028 | 0.016 |
| Cathepsin K | NM_000396 | 202450_s_at | 2596.69 | 819.30 | -3.17 | 0.01 | <0.01 | 336.08 | -7.73 | 0.0011 | 0.0096 |
| Cathepsin L1 | NM_001912 | 202087_s_at | 769.84 | 326.66 | -2.36 | 0.012 | <0.01 | 124.45 | -6.19 | 0.00076 | 0.0096 |
| Cathepsin O | AV729484 | 203758_at | 231.63 | 68.18 | -3.40 | 0.000059 | <0.01 | 50.30 | -4.61 | 0.000032 | 0.016 |
| Cathepsin S | NM_004079 | 202902_s_at | 204.78 | 34.18 | -5.99 | 0.0031 | <0.01 | 15.96 | -12.83 | 0.0017 | 0.049 |
| Cbp/p300-interacting transactivator, with Glu/Asp-rich carboxy-terminal domain, 2 | NM_006079 | 207980_s_at | 159.39 | 70.90 | -2.25 | 0.0019 | 0.18 | 23.79 | -6.70 | 0.000075 | 0.049 |
| Cbp/p300-interacting transactivator, with Glu/Asp-rich carboxy-terminal domain, 2 | AF109161 | 209357_at | 280.28 | 114.03 | -2.46 | 0.00026 | <0.01 | 42.05 | -6.67 | 0.000011 | <0.01 |
| CCAAT/enhancer binding protein (C/EBP), beta | AL564683 | 212501_at | 1202.05 | 707.05 | -1.70 | 0.011 | <0.01 | 503.99 | -2.39 | 0.00021 | <0.01 |
| CCAAT/enhancer binding protein (C/EBP), delta | NM_005195 | 203973_s_at | 748.34 | 303.41 | -2.47 | 0.0043 | <0.01 | 262.39 | -2.85 | 0.0012 | 0.016 |
| CD47 molecule | Z25521 | 211075_s_at | 504.30 | 249.18 | -2.02 | 0.000039 | <0.01 | 137.72 | -3.66 | 0.000001 | <0.01 |
| CD47 molecule | BG230614 | 213857_s_at | 817.33 | 352.13 | -2.32 | 0.00024 | <0.01 | 248.97 | -3.28 | 0.000099 | <0.01 |
| CD82 molecule | NM_002231 | 203904_x_at | 245.45 | 122.53 | -2.00 | 0.0072 | 0.012 | 144.04 | -1.70 | 0.016 | 0.22 |
| Cell division cycle 25 homolog B (S. pombe) | NM_021873 | 201853_s_at | 194.82 | 134.76 | -1.45 | 0.13 | 0.41 | 70.33 | -2.77 | 0.0013 | 0.073 |
| Cellular repressor of E1A-stimulated genes 1 | NM_003851 | 201200_at | 301.97 | 157.97 | -1.91 | 0.001 | <0.01 | 103.54 | -2.92 | 0.000062 | 0.016 |
| Cellular retinoic acid binding protein 2 | NM_001878 | 202575_at | 306.27 | 254.54 | -1.20 | 0.42 | 0.41 | 113.36 | -2.70 | 0.0048 | 0.073 |
| Chemokine (C-C motif) ligand 11 | D49372 | 210133_at | 529.84 | 36.18 | -14.64 | 0.0021 | <0.01 | 31.02 | -17.08 | 0.002 | 0.028 |
| Chemokine (C-C motif) ligand 2 | S69738 | 216598_s_at | 2676.36 | 385.53 | -6.94 | 0.000055 | <0.01 | 128.29 | -20.86 | 0.00003 | <0.01 |
| Chemokine (C-C motif) ligand 7 | NM_006273 | 208075_s_at | 215.28 | 23.08 | -9.33 | 0.000096 | <0.01 | 10.64 | -20.23 | 0.000062 | 0.016 |
| Chemokine (C-X-C motif) ligand 1 | NM_001511 | 204470_at | 637.05 | 123.92 | -5.14 | <0.000001 | <0.01 | 26.31 | -24.21 | <0.000001 | <0.01 |
| Chemokine (C-X-C motif) ligand 10 | NM_001565 | 204533_at | 771.16 | 19.22 | -40.12 | 0.00034 | <0.01 | 9.48 | -81.32 | 0.00031 | <0.01 |
| Chemokine (C-X-C motif) ligand 11 | AF030514 | 210163_at | 179.86 | 4.99 | -36.04 | 0.0027 | <0.01 | 4.24 | -42.42 | 0.0026 | 0.073 |
| Chemokine (C-X-C motif) ligand 11 | AF002985 | 211122_s_at | 165.76 | 8.57 | -19.34 | 0.00058 | <0.01 | 3.97 | -41.72 | 0.00048 | 0.049 |
| Chemokine (C-X-C motif) ligand 12 | NM_000609 | 203666_at | 195.34 | 130.61 | -1.50 | 0.046 | 0.401 | 26.23 | -7.45 | 0.000005 | 0.0096 |
| Chemokine (C-X-C motif) ligand 12 | U19495 | 209687_at | 840.36 | 446.63 | -1.88 | 0.0003 | <0.01 | 48.22 | -17.43 | <0.000001 | <0.01 |
| Chemokine (C-X-C motif) ligand 2 | M57731 | 209774_x_at | 144.94 | 25.11 | -5.77 | 0.000073 | <0.01 | 3.58 | -40.49 | 0.000035 | 0.028 |
| Chemokine (C-X-C motif) ligand 6 | NM_002993 | 206336_at | 425.78 | 90.06 | -4.73 | 0.0019 | <0.01 | 26.61 | -16.00 | 0.00061 | 0.016 |
|  |  |  |  | **SSc-ILD** | | | | **IPF** | | | |
| **Annotation** | **Accession** | **Probe Set ID** | **Control mean** | **Ssc-ILD mean** | **Fold change** | **p value** | **q-value(%)** | **IPF mean** | **Fold change** | **p value** | **q-value(%)** |
| Chitinase 3-like 1 | M80927 | 209396_s_at | 272.70 | 70.89 | -3.85 | 0.068 | 0.0303 | 20.12 | -13.55 | 0.019 | 0.13 |
| Chromosome 10 open reading frame 10 | AL136653 | 209183_s_at | 208.79 | 13.64 | -15.31 | 0.0062 | <0.01 | 5.45 | -38.29 | 0.0049 | 0.073 |
| Chromosome 13 open reading frame 15 | NM_014059 | 218723_s_at | 212.39 | 76.00 | -2.79 | 0.0001 | <0.01 | 178.95 | -1.19 | 0.51 | 0.29 |
| Chromosome 14 open reading frame 132 | NM_020215 | 218820_at | 300.39 | 148.51 | -2.02 | 0.0058 | <0.01 | 164.10 | -1.83 | 0.014 | 0.13 |
| Chromosome 19 open reading frame 66 | NM_018381 | 218429_s_at | 149.14 | 40.52 | -3.68 | 0.00032 | <0.01 | 19.44 | -7.67 | 0.000093 | 0.049 |
| Chromosome 19 open reading frame 66 | AI862559 | 53720_at | 268.09 | 66.14 | -4.05 | 0.000028 | <0.01 | 31.15 | -8.61 | 0.000011 | <0.01 |
| Coagulation factor II (thrombin) receptor | NM_001992 | 203989_x_at | 264.80 | 106.92 | -2.48 | 0.033 | 0.022 | 234.26 | -1.13 | 0.66 | 0.29 |
| Coiled-coil domain containing 85B | NM_006848 | 204610_s_at | 225.98 | 142.19 | -1.59 | 0.019 | 0.22 | 93.65 | -2.41 | 0.000014 | 0.028 |
| Cold shock domain protein A | NM_003651 | 201161_s_at | 320.42 | 189.62 | -1.69 | 0.00032 | <0.01 | 134.76 | -2.38 | 0.000022 | 0.016 |
| Collagen, type VI, alpha 1 | AI141603 | 212091_s_at | 500.27 | 440.38 | -1.14 | 0.77 | 0.41 | 195.03 | -2.57 | 0.012 | 0.073 |
| Collagen, type VI, alpha 2 | AY029208 | 209156_s_at | 1630.83 | 1327.26 | -1.23 | 0.61 | 0.048 | 637.08 | -2.56 | 0.017 | 0.073 |
| Collectin sub-family member 12 | NM_030781 | 221019_s_at | 114.75 | 51.82 | -2.21 | 0.048 | 0.41 | 7.15 | -16.04 | 0.00084 | 0.073 |
| Colony stimulating factor 1 (macrophage) | M37435 | 209716_at | 247.62 | 63.15 | -3.92 | 0.000006 | <0.01 | 24.61 | -10.06 | 0.000004 | <0.01 |
| Complement component 1, r subcomponent | AL573058 | 212067_s_at | 1448.63 | 350.18 | -4.14 | 0.000064 | <0.01 | 392.83 | -3.69 | 0.00012 | <0.01 |
| Complement component 1, r subcomponent-like | NM_016546 | 218983_at | 145.04 | 53.72 | -2.70 | 0.000065 | 0.14 | 43.64 | -3.32 | 0.000084 | 0.073 |
| Complement component 1, s subcomponent | M18767 | 208747_s_at | 2721.67 | 757.25 | -3.59 | 0.000003 | <0.01 | 739.44 | -3.68 | 0.00001 | <0.01 |
| Complement component 3 | NM_000064 | 217767_at | 457.89 | 107.33 | -4.27 | 0.00031 | <0.01 | 14.40 | -31.79 | 0.000072 | <0.01 |
| Complement factor B | NM_001710 | 202357_s_at | 837.04 | 42.64 | -19.63 | 0.0036 | <0.01 | 13.90 | -60.23 | 0.0029 | 0.028 |
| Complement factor H | X56210 | 215388_s_at | 437.48 | 118.72 | -3.68 | 0.018 | <0.01 | 144.44 | -3.03 | 0.026 | 0.13 |
| C-type lectin domain family 2, member B | BC005254 | 209732_at | 595.30 | 126.45 | -4.71 | 0.022 | <0.01 | 21.51 | -27.68 | 0.0074 | 0.049 |
| C-type lectin domain family 3, member B | NM_003278 | 205200_at | 144.76 | 42.80 | -3.38 | 0.041 | 0.048 | 40.22 | -3.60 | 0.035 | 0.29 |
| Cyclin G1 | BC000196 | 208796_s_at | 606.50 | 356.97 | -1.70 | 0.01 | <0.01 | 250.45 | -2.42 | 0.000028 | <0.01 |
| Cyclin-dependent kinase inhibitor 2A (melanoma, p16, inhibits CDK4) | U38945 | 209644_x_at | 312.20 | 139.48 | -2.24 | 0.011 | <0.01 | 125.88 | -2.48 | 0.0072 | 0.073 |
| Cytochrome b-245, alpha polypeptide | NM_000101 | 203028_s_at | 274.17 | 189.59 | -1.45 | 0.0084 | 0.22 | 117.55 | -2.33 | 0.000053 | 0.028 |
| Cytochrome b5 type A (microsomal) | NM_001914 | 207843_x_at | 415.89 | 120.97 | -3.44 | 0.000026 | <0.01 | 118.78 | -3.50 | 0.000038 | <0.01 |
| Cytochrome b5 type A (microsomal) | M22865 | 209366_x_at | 478.26 | 130.55 | -3.66 | 0.000017 | <0.01 | 127.10 | -3.76 | 0.000024 | <0.01 |
| Cytochrome b5 type A (microsomal) | M22976 | 215726_s_at | 251.43 | 77.91 | -3.23 | 0.00076 | <0.01 | 91.89 | -2.74 | 0.0015 | 0.073 |
| Cytochrome P450, family 1, subfamily B, polypeptide 1 | AU154504 | 202435_s_at | 158.26 | 71.57 | -2.21 | 0.022 | 0.22 | 29.11 | -5.44 | 0.000085 | 0.049 |
| Cytochrome P450, family 1, subfamily B, polypeptide 1 | AU144855 | 202436_s_at | 467.47 | 216.00 | -2.16 | 0.037 | <0.01 | 64.46 | -7.25 | 0.000052 | <0.01 |
| Cytochrome P450, family 1, subfamily B, polypeptide 1 | NM_000104 | 202437_s_at | 140.66 | 69.35 | -2.03 | 0.053 | 0.34 | 21.45 | -6.56 | 0.00061 | 0.073 |
| DEAD (Asp-Glu-Ala-Asp) box polypeptide 58 | NM_014314 | 218943_s_at | 391.58 | 49.78 | -7.87 | 0.002 | <0.01 | 39.22 | -9.98 | 0.0017 | 0.028 |
| DEAD (Asp-Glu-Ala-Asp) box polypeptide 60 | NM_017631 | 218986_s_at | 253.21 | 44.30 | -5.72 | 0.000028 | <0.01 | 21.35 | -11.86 | 0.000015 | <0.01 |
| Decorin | AI281593 | 209335_at | 2232.37 | 1027.07 | -2.17 | 0.00015 | <0.01 | 1636.16 | -1.36 | 0.019 | 0.073 |
| Decorin | AF138303 | 211813_x_at | 6527.54 | 3165.57 | -2.06 | 0.000011 | <0.01 | 4571.15 | -1.43 | 0.0014 | 0.0096 |
| Dihydropyrimidinase-like 2 | NM_001386 | 200762_at | 552.25 | 265.70 | -2.08 | 0.000009 | <0.01 | 207.00 | -2.67 | 0.0011 | <0.01 |
| Dipeptidyl-peptidase 4 | M80536 | 203716_s_at | 327.81 | 103.26 | -3.17 | 0.02 | <0.01 | 12.68 | -25.85 | 0.0015 | 0.028 |
| Dipeptidyl-peptidase 4 | NM_001935 | 203717_at | 396.67 | 125.03 | -3.17 | 0.0027 | <0.01 | 38.96 | -10.18 | 0.0002 | 0.0096 |
| Dipeptidyl-peptidase 4 | M74777 | 211478_s_at | 216.90 | 85.20 | -2.55 | 0.042 | 0.0303 | 16.86 | -12.87 | 0.0013 | 0.049 |
| Disabled homolog 2, mitogen-responsive phosphoprotein | N21202 | 201278_at | 419.24 | 214.19 | -1.96 | 0.0012 | <0.01 | 181.09 | -2.32 | 0.0024 | 0.0096 |
| Disabled homolog 2, mitogen-responsive phosphoprotein | BC003064 | 201279_s_at | 621.39 | 365.09 | -1.70 | 0.0048 | <0.01 | 230.32 | -2.70 | 0.000006 | <0.01 |
| Disabled homolog 2, mitogen-responsive phosphoprotein | NM_001343 | 201280_s_at | 1043.34 | 615.46 | -1.70 | 0.016 | <0.01 | 349.39 | -2.99 | 0.000007 | <0.01 |
| Disabled homolog 2, mitogen-responsive phosphoprotein | AF188298 | 210757_x_at | 742.02 | 425.49 | -1.74 | 0.0015 | <0.01 | 277.12 | -2.68 | <0.000001 | <0.01 |
| DNA-damage regulated autophagy modulator 1 | NM_018370 | 218627_at | 1126.08 | 269.16 | -4.18 | 0.000001 | <0.01 | 207.43 | -5.43 | 0.000002 | <0.01 |
| DNA-damage-inducible transcript 3 | BC003637 | 209383_at | 285.01 | 133.09 | -2.14 | 0.00069 | <0.01 | 107.59 | -2.65 | 0.000031 | 0.016 |
|  |  |  |  | **SSc-ILD** | | | | **IPF** | | | |
| **Annotation** | **Accession** | **Probe Set ID** | **Control mean** | **Ssc-ILD mean** | **Fold change** | **p value** | **q-value(%)** | **IPF mean** | **Fold change** | **p value** | **q-value(%)** |
| Ectonucleotide pyrophosphatase/phosphodiesterase 2 | L35594 | 209392_at | 555.82 | 240.57 | -2.31 | 0.17 | 0.048 | 16.26 | -34.18 | 0.022 | 0.073 |
| Ectonucleotide pyrophosphatase/phosphodiesterase 2 | D45421 | 210839_s_at | 437.21 | 189.31 | -2.31 | 0.18 | 0.048 | 23.10 | -18.93 | 0.029 | 0.13 |
| EGF-containing fibulin-like extracellular matrix protein 1 | AI826799 | 201842_s_at | 970.26 | 463.10 | -2.10 | 0.0014 | <0.01 | 425.52 | -2.28 | 0.00072 | 0.016 |
| Endoplasmic reticulum protein 29 | NM_006817 | 201216_at | 430.56 | 301.73 | -1.43 | 0.25 | 0.048 | 169.67 | -2.54 | 0.012 | 0.073 |
| Endothelial PAS domain protein 1 | AF052094 | 200878_at | 1875.53 | 612.98 | -3.06 | 0.001 | <0.01 | 443.70 | -4.23 | 0.00043 | <0.01 |
| Epidermal growth factor receptor | AW157070 | 201983_s_at | 406.72 | 183.91 | -2.21 | 0.00092 | <0.01 | 130.82 | -3.11 | 0.00018 | 0.016 |
| Epithelial membrane protein 1 | NM_001423 | 201324_at | 510.69 | 304.00 | -1.68 | 0.02 | <0.01 | 246.97 | -2.07 | 0.0097 | 0.028 |
| Epithelial membrane protein 1 | NM_001423 | 201325_s_at | 371.38 | 216.33 | -1.72 | 0.021 | 0.012 | 139.04 | -2.67 | 0.00019 | 0.016 |
| Erythrocyte membrane protein band 4.1-like 2 | BF511685 | 201718_s_at | 198.21 | 82.75 | -2.40 | 0.0039 | <0.01 | 74.82 | -2.65 | 0.0029 | 0.073 |
| Erythrocyte membrane protein band 4.1-like 2 | NM_001431 | 201719_s_at | 306.45 | 128.91 | -2.38 | 0.00039 | <0.01 | 115.89 | -2.64 | 0.00027 | 0.028 |
| Eukaryotic translation initiation factor 2-alpha kinase 2 | NM_002759 | 204211_x_at | 213.57 | 58.72 | -3.64 | 0.000032 | <0.01 | 40.97 | -5.21 | 0.000016 | 0.016 |
| Eukaryotic translation initiation factor 2-alpha kinase 2 | AV755522 | 213294_at | 318.33 | 101.37 | -3.14 | <0.000001 | <0.01 | 84.68 | -3.76 | <0.000001 | <0.01 |
| Extracellular matrix protein 1 | U65932 | 209365_s_at | 306.85 | 231.41 | -1.33 | 0.21 | 0.26 | 133.46 | -2.30 | 0.0026 | 0.073 |
| Family with sequence similarity 21, member A | AL080183 | 212370_x_at | 380.64 | 215.43 | -1.77 | 0.06 | 0.0303 | 154.24 | -2.47 | 0.0037 | 0.073 |
| Family with sequence similarity 21, member A | AV728658 | 214946_x_at | 311.15 | 170.14 | -1.83 | 0.11 | 0.048 | 92.87 | -3.35 | 0.0045 | 0.073 |
| Family with sequence similarity 21, member A with sequence similarity 21, member D | W68158 | 212929_s_at | 126.84 | 69.10 | -1.84 | 0.039 | 0.41 | 26.29 | -4.83 | 0.00032 | 0.073 |
| Family with sequence similarity 21, member C | BC006456 | 211068_x_at | 333.52 | 180.59 | -1.85 | 0.025 | 0.012 | 108.12 | -3.08 | 0.00072 | 0.028 |
| Fas (TNF receptor superfamily, member 6) | AA164751 | 204780_s_at | 290.51 | 160.27 | -1.81 | 0.00099 | <0.01 | 97.56 | -2.98 | 0.000005 | 0.0096 |
| Fas (TNF receptor superfamily, member 6) | X83493 | 215719_x_at | 209.52 | 122.97 | -1.70 | 0.021 | 0.26 | 66.97 | -3.13 | 0.00041 | 0.049 |
| Fas (TNF receptor superfamily, member 6) | Z70519 | 216252_x_at | 185.51 | 111.91 | -1.66 | 0.022 | 0.34 | 64.46 | -2.88 | 0.00045 | 0.073 |
| Ferritin, heavy polypeptide 1 | NM_002032 | 200748_s_at | 6342.52 | 3812.71 | -1.66 | 0.000045 | <0.01 | 2521.92 | -2.51 | 0.002 | <0.01 |
| Ferritin, heavy polypeptide 1 | AA083483 | 214211_at | 1019.26 | 443.74 | -2.30 | 0.000004 | <0.01 | 162.93 | -6.26 | <0.000001 | <0.01 |
| Ferritin, heavy polypeptide 1 pseudogene 5 | J04755 | 211628_x_at | 5465.02 | 3793.88 | -1.44 | 0.00058 | <0.01 | 2713.38 | -2.01 | 0.0034 | <0.01 |
| Ferritin, light polypeptide | BG538564 | 213187_x_at | 4628.14 | 3249.06 | -1.42 | 0.0019 | <0.01 | 2221.95 | -2.08 | 0.00021 | <0.01 |
| Fibroblast growth factor 7 | NM_002009 | 205782_at | 159.10 | 49.53 | -3.21 | 0.015 | 0.022 | 91.83 | -1.73 | 0.11 | 0.29 |
| Fibronectin leucine rich transmembrane protein 2 | NM_013231 | 204359_at | 218.68 | 190.94 | -1.15 | 0.43 | 0.41 | 106.97 | -2.04 | 0.0047 | 0.13 |
| Fibulin 1 | NM_001996 | 201787_at | 343.70 | 155.90 | -2.20 | 0.026 | 0.012 | 54.46 | -6.31 | 0.002 | 0.049 |
| Fibulin 1 | Z95331 | 202994_s_at | 545.28 | 189.83 | -2.87 | 0.00065 | <0.01 | 79.77 | -6.84 | 0.0001 | <0.01 |
| Fibulin 1 | NM_006486 | 202995_s_at | 448.92 | 164.51 | -2.73 | 0.0016 | <0.01 | 63.93 | -7.02 | 0.00019 | 0.0096 |
| Fibulin 2 | NM_001998 | 203886_s_at | 266.86 | 189.90 | -1.41 | 0.24 | 0.41 | 52.53 | -5.08 | 0.00073 | 0.049 |
| FK506 binding protein 9, 63 kDa | AL050187 | 212169_at | 735.67 | 354.16 | -2.08 | 0.015 | <0.01 | 282.35 | -2.61 | 0.0049 | 0.049 |
| Flavin containing monooxygenase 2 (non-functional) | BC005894 | 211726_s_at | 530.43 | 15.73 | -33.73 | 0.017 | <0.01 | 11.42 | -46.43 | 0.016 | 0.073 |
| Four and a half LIM domains 1 | NM_001449 | 201540_at | 739.72 | 435.67 | -1.70 | 0.024 | <0.01 | 296.83 | -2.49 | 0.00074 | 0.016 |
| Four and a half LIM domains 1 | AF098518 | 210298_x_at | 251.59 | 176.99 | -1.42 | 0.059 | 0.34 | 85.76 | -2.93 | 0.000002 | 0.0096 |
| Four and a half LIM domains 1 | AF063002 | 210299_s_at | 277.83 | 174.61 | -1.59 | 0.03 | 0.0303 | 111.52 | -2.49 | 0.00069 | 0.049 |
| Four and a half LIM domains 1 | AF220153 | 214505_s_at | 268.68 | 191.76 | -1.40 | 0.037 | 0.29 | 92.34 | -2.91 | 0.000001 | <0.01 |
| Frizzled homolog 7 (Drosophila) | NM_003507 | 203706_s_at | 189.02 | 112.63 | -1.68 | 0.017 | 0.29 | 80.42 | -2.35 | 0.0079 | 0.13 |
| FYN oncogene related to SRC, FGR, YES | M14333 | 210105_s_at | 372.83 | 164.63 | -2.26 | 0.0025 | <0.01 | 92.61 | -4.03 | 0.00024 | 0.016 |
| FYN oncogene related to SRC, FGR, YES | S74774 | 216033_s_at | 155.81 | 71.26 | -2.19 | 0.012 | 0.22 | 28.21 | -5.52 | 0.00047 | 0.073 |
| G protein-coupled receptor 124 | BF511315 | 221814_at | 237.02 | 120.92 | -1.96 | 0.0042 | <0.01 | 111.67 | -2.12 | 0.0007 | 0.073 |
| G protein-coupled receptor 37 (endothelin receptor type B-like) | U87460 | 209631_s_at | 170.94 | 23.29 | -7.34 | 0.022 | 0.022 | 28.74 | -5.95 | 0.026 | 0.22 |
| G protein-coupled receptor kinase 5 | NM_005308 | 204396_s_at | 222.36 | 72.52 | -3.07 | 0.0011 | <0.01 | 59.74 | -3.72 | 0.00068 | 0.049 |
| G0/G1switch 2 | NM_015714 | 213524_s_at | 244.86 | 90.92 | -2.69 | 0.028 | 0.022 | 28.33 | -8.64 | 0.0011 | 0.049 |
|  |  |  |  | **SSc-ILD** | | | | **IPF** | | | |
| **Annotation** | **Accession** | **Probe Set ID** | **Control mean** | **Ssc-ILD mean** | **Fold change** | **p value** | **q-value(%)** | **IPF mean** | **Fold change** | **p value** | **q-value(%)** |
| Galactosidase, beta 1 | NM_000404 | 201576_s_at | 259.92 | 191.15 | -1.36 | 0.13 | 0.41 | 125.02 | -2.08 | 0.0012 | 0.073 |
| Gelsolin | NM_000177 | 200696_s_at | 442.81 | 266.45 | -1.66 | 0.0023 | <0.01 | 163.34 | -2.71 | 0.000002 | <0.01 |
| Glioma tumor suppressor candidate region gene 2 | NM_015710 | 217807_s_at | 766.15 | 543.07 | -1.41 | 0.027 | <0.01 | 377.91 | -2.03 | 0.000008 | <0.01 |
| Glucan (1,4-alpha-), branching enzyme 1 | NM_000158 | 203282_at | 526.85 | 401.38 | -1.31 | 0.18 | 0.048 | 209.00 | -2.52 | 0.0018 | 0.028 |
| Glutaredoxin (thioltransferase) | NM_002064 | 206662_at | 793.08 | 265.44 | -2.99 | 0.0016 | <0.01 | 324.75 | -2.44 | 0.0035 | 0.049 |
| Glutaredoxin (thioltransferase) | AF162769 | 209276_s_at | 575.63 | 184.00 | -3.13 | 0.0027 | <0.01 | 225.31 | -2.55 | 0.0053 | 0.049 |
| Glutathione S-transferase kappa 1 | NM_015917 | 217751_at | 250.16 | 165.11 | -1.52 | 0.0025 | 0.18 | 122.74 | -2.04 | 0.000018 | 0.028 |
| Glycophorin C | NM_002101 | 202947_s_at | 212.41 | 93.63 | -2.27 | 0.0017 | <0.01 | 62.25 | -3.41 | 0.000007 | 0.016 |
| Glycoprotein (transmembrane) nmb | NM_002510 | 201141_at | 981.23 | 386.26 | -2.54 | 0.082 | 0.022 | 75.67 | -12.97 | 0.0061 | 0.049 |
| Glypican 1 | NM_002081 | 202756_s_at | 240.67 | 159.79 | -1.51 | 0.0014 | 0.18 | 119.00 | -2.02 | 0.019 | 0.028 |
| GPS, PLAT and transmembrane domain-containing protein | AA308853 | 214035_x_at | 367.73 | 184.24 | -2.00 | 0.001 | <0.01 | 163.93 | -2.24 | 0.000052 | 0.016 |
| Granulin | NM_002087 | 200678_x_at | 544.33 | 261.34 | -2.08 | 0.019 | <0.01 | 195.12 | -2.79 | 0.0021 | 0.049 |
| Granulin | BC000324 | 211284_s_at | 827.12 | 350.51 | -2.36 | 0.000028 | <0.01 | 330.14 | -2.51 | 0.000021 | <0.01 |
| Granulin | AK023348 | 216041_x_at | 945.17 | 386.72 | -2.44 | 0.000063 | <0.01 | 368.63 | -2.56 | 0.00004 | <0.01 |
| Gremlin 1 | AF154054 | 218468_s_at | 1679.84 | 1434.29 | -1.17 | 0.49 | 0.048 | 583.46 | -2.88 | 0.000001 | <0.01 |
| Gremlin 1 | NM_013372 | 218469_at | 1540.86 | 1205.90 | -1.28 | 0.27 | 0.048 | 617.65 | -2.49 | 0.00026 | <0.01 |
| Growth arrest-specific 1 | NM_002048 | 204457_s_at | 383.22 | 81.59 | -4.70 | 0.00019 | <0.01 | 17.21 | -22.27 | 0.000059 | <0.01 |
| Growth differentiation factor 15 | AF003934 | 221577_x_at | 185.79 | 84.93 | -2.19 | 0.041 | 0.0303 | 43.82 | -4.24 | 0.00072 | 0.073 |
| Guanosine monophosphate reductase | NM_006877 | 204187_at | 143.60 | 45.31 | -3.17 | 0.000024 | 0.094 | 32.88 | -4.37 | 0.000018 | 0.049 |
| Guanylate binding protein 1, interferon-inducible, 67kDa | BC002666 | 202269_x_at | 626.71 | 191.65 | -3.27 | 0.017 | <0.01 | 228.02 | -2.75 | 0.025 | 0.073 |
| Guanylate binding protein 1, interferon-inducible, 67kDa | NM_002053 | 202270_at | 271.06 | 90.06 | -3.01 | 0.0071 | <0.01 | 184.66 | -1.47 | 0.21 | 0.29 |
| Guanylate binding protein 2, interferon-inducible | NM_004120 | 202748_at | 216.57 | 44.17 | -4.90 | 0.00036 | <0.01 | 54.98 | -3.94 | 0.0006 | 0.049 |
| H2B histone family, member S | NM_017445 | 208579_x_at | 280.55 | 140.12 | -2.00 | 0.026 | 0.022 | 186.24 | -1.51 | 0.09 | 0.29 |
| Heat-responsive protein 12 | N54448 | 203790_s_at | 208.75 | 80.66 | -2.59 | 0.039 | 0.0303 | 49.74 | -4.20 | 0.014 | 0.13 |
| Hect domain and RLD 5 | NM_016323 | 219863_at | 340.15 | 73.86 | -4.61 | 0.00074 | <0.01 | 59.57 | -5.71 | 0.00052 | 0.028 |
| Hect domain and RLD 6 | NM_017912 | 219352_at | 274.04 | 25.73 | -10.65 | 0.000007 | <0.01 | 16.69 | -16.42 | 0.000007 | <0.01 |
| HEG homolog 1 (zebrafish) | AI148659 | 213069_at | 509.38 | 184.16 | -2.77 | 0.0027 | <0.01 | 255.64 | -1.99 | 0.023 | 0.073 |
| Heme binding protein 1 | NM_015987 | 218450_at | 475.01 | 223.29 | -2.13 | 0.0015 | <0.01 | 237.24 | -2.00 | 0.0024 | 0.049 |
| Hemoglobin, gamma A | NM_000184 | 204419_x_at | 177.37 | 29.49 | -6.01 | 0.027 | 0.022 | 20.82 | -8.52 | 0.016 | 0.13 |
| Hemoglobin, gamma A | NM_000559 | 204848_x_at | 157.97 | 19.84 | -7.96 | 0.022 | 0.022 | 11.54 | -13.69 | 0.016 | 0.13 |
| Hemoglobin, gamma A | AI133353 | 213515_x_at | 182.98 | 32.54 | -5.62 | 0.026 | 0.022 | 25.84 | -7.08 | 0.021 | 0.13 |
| Hepatocyte growth factor (hepapoietin A) | X16323 | 209960_at | 215.20 | 40.31 | -5.34 | 0.014 | <0.01 | 10.42 | -20.65 | 0.0049 | 0.073 |
| Hexosaminidase A | AL523158 | 201765_s_at | 209.58 | 120.79 | -1.74 | 0.091 | 0.34 | 71.53 | -2.93 | 0.0072 | 0.13 |
| Hexosaminidase B (beta polypeptide) | NM_000521 | 201944_at | 728.46 | 363.34 | -2.00 | 0.011 | <0.01 | 199.01 | -3.66 | 0.00048 | 0.0096 |
| Histamine N-methyltransferase | NM_006895 | 204112_s_at | 174.98 | 35.22 | -4.97 | 0.012 | 0.012 | 110.13 | -1.59 | 0.27 | 0.29 |
| Histone cluster 1, H2ac | AL353759 | 215071_s_at | 321.95 | 84.55 | -3.81 | 0.0043 | <0.01 | 56.02 | -5.75 | 0.0021 | 0.049 |
| Histone cluster 1, H2bd | BC002842 | 209911_x_at | 258.85 | 126.90 | -2.04 | 0.036 | 0.0303 | 141.84 | -1.83 | 0.039 | 0.22 |
| Histone cluster 2, H2aa3 | AI313324 | 214290_s_at | 720.22 | 247.66 | -2.91 | 0.0011 | <0.01 | 196.48 | -3.67 | 0.00058 | 0.0096 |
| Histone cluster 2, H2aa3 | NM_003516 | 218280_x_at | 428.53 | 156.57 | -2.74 | 0.0010 | <0.01 | 113.48 | -3.78 | 0.00039 | 0.016 |
| Hydroxysteroid (11-beta) dehydrogenase 1 | NM_005525 | 205404_at | 430.46 | 78.97 | -5.45 | 0.0093 | <0.01 | 116.09 | -3.71 | 0.016 | 0.073 |
| Hypothetical protein LOC100130633 | AI805560 | 213698_at | 240.54 | 120.27 | -2.00 | 0.00034 | <0.01 | 99.84 | -2.41 | 0.00012 | 0.049 |
| Immediate early response 5 | NM_016545 | 218611_at | 210.53 | 152.35 | -1.38 | 0.05 | 0.41 | 92.57 | -2.27 | 0.0034 | 0.049 |
| Insulin-like growth factor 2 receptor | BG031974 | 201392_s_at | 274.37 | 151.96 | -1.81 | 0.0055 | 0.012 | 107.39 | -2.55 | 0.00036 | 0.049 |
| Insulin-like growth factor binding protein 6 | NM_002178 | 203851_at | 1312.90 | 491.34 | -2.67 | 0.0016 | <0.01 | 425.56 | -3.09 | 0.001 | 0.0096 |
|  |  |  |  | **SSc-ILD** | | | |  | **IPF** | | |
| **Annotation** | **Accession** | **Probe Set ID** | **Control mean** | **Ssc-ILD mean** | **Fold change** | **p value** | **q-value(%)** | **IPF mean** | **Fold change** | **p value** | **q-value(%)** |
| Integral membrane protein 2B | NM_021999 | 217731_s_at | 688.86 | 310.07 | -2.22 | 0.002 | <0.01 | 415.83 | -1.66 | 0.019 | 0.073 |
| Integral membrane protein 2B | AF092128 | 217732_s_at | 1312.72 | 555.97 | -2.36 | 0.000008 | <0.01 | 583.11 | -2.25 | 0.000033 | <0.01 |
| Intercellular adhesion molecule 1 | AI608725 | 202637_s_at | 124.28 | 33.47 | -3.71 | 0.000004 | 0.14 | 13.99 | -8.88 | 0.000001 | 0.028 |
| Interferon gamma receptor 1 | NM_000416 | 202727_s_at | 212.61 | 119.84 | -1.77 | 0.00003 | 0.14 | 95.35 | -2.23 | 0.00013 | 0.028 |
| Interferon induced transmembrane protein 1 (9-27) | NM_003641 | 201601_x_at | 4134.68 | 897.96 | -4.60 | <0.000001 | <0.01 | 334.87 | -12.35 | <0.000001 | <0.01 |
| Interferon induced transmembrane protein 1 (9-27) | AA749101 | 214022_s_at | 3698.03 | 443.31 | -8.34 | <0.000001 | <0.01 | 61.36 | -60.27 | <0.000001 | <0.01 |
| Interferon induced transmembrane protein 2 (1-8D) | NM_006435 | 201315_x_at | 4413.29 | 1668.79 | -2.64 | <0.000001 | <0.01 | 886.01 | -4.98 | <0.000001 | <0.01 |
| Interferon induced transmembrane protein 3 (1-8U) | BF338947 | 212203_x_at | 4971.02 | 2039.45 | -2.44 | <0.000001 | <0.01 | 1243.06 | -4.00 | <0.000001 | <0.01 |
| Interferon induced with helicase C domain 1 | NM_022168 | 219209_at | 354.78 | 29.67 | -11.96 | 0.0028 | <0.01 | 12.46 | -28.48 | 0.0021 | 0.049 |
| Interferon regulatory factor 1 | NM_002198 | 202531_at | 198.74 | 55.50 | -3.58 | 0.000048 | <0.01 | 36.27 | -5.48 | 0.000029 | 0.016 |
| Interferon regulatory factor 7 | NM_004030 | 208436_s_at | 390.28 | 39.53 | -9.87 | 0.00082 | <0.01 | 23.64 | -16.51 | 0.00062 | 0.016 |
| Interferon regulatory factor 9 | NM_006084 | 203882_at | 307.42 | 107.38 | -2.86 | <0.000001 | <0.01 | 63.63 | -4.83 | <0.000001 | <0.01 |
| Interferon, alpha-inducible protein 27 | NM_005532 | 202411_at | 897.46 | 78.80 | -11.39 | 0.0026 | <0.01 | 7.41 | -121.11 | 0.0016 | 0.016 |
| Interferon, alpha-inducible protein 6 | NM_022873 | 204415_at | 1196.56 | 93.67 | -12.77 | 0.00043 | <0.01 | 11.82 | -101.20 | 0.00027 | <0.01 |
| Interferon, gamma-inducible protein 16 | NM_005531 | 206332_s_at | 764.46 | 203.44 | -3.76 | 0.000021 | <0.01 | 126.97 | -6.02 | 0.00001 | <0.01 |
| Interferon, gamma-inducible protein 16 | BG256677 | 208965_s_at | 499.74 | 130.48 | -3.83 | 0.0002 | <0.01 | 61.42 | -8.14 | 0.000062 | <0.01 |
| Interferon, gamma-inducible protein 16 | AF208043 | 208966_x_at | 957.31 | 253.28 | -3.78 | 0.000007 | <0.01 | 149.78 | -6.39 | 0.000004 | <0.01 |
| Interferon, gamma-inducible protein 30 | NM_006332 | 201422_at | 199.42 | 27.67 | -7.21 | 0.0061 | <0.01 | 7.99 | -24.95 | 0.0032 | 0.073 |
| Interferon-induced protein 35 | BC001356 | 209417_s_at | 455.61 | 49.12 | -9.28 | 0.000082 | <0.01 | 18.13 | -25.13 | 0.000062 | <0.01 |
| Interferon-induced protein 44 | NM_006417 | 214453_s_at | 335.01 | 41.06 | -8.16 | 0.000038 | <0.01 | 16.97 | -19.74 | 0.000027 | <0.01 |
| Interferon-induced protein 44-like | NM_006820 | 204439_at | 370.65 | 15.36 | -24.13 | 0.00033 | <0.01 | 11.63 | -31.86 | 0.0003 | 0.0096 |
| Interferon-induced protein with tetratricopeptide repeats 1 | NM_001548 | 203153_at | 1744.16 | 82.58 | -21.12 | 0.000039 | <0.01 | 5.43 | -321.41 | 0.000033 | <0.01 |
| Interferon-induced protein with tetratricopeptide repeats 2 | BE888744 | 217502_at | 707.12 | 26.10 | -27.09 | 0.0096 | <0.01 | 13.12 | -53.90 | 0.0087 | 0.049 |
| Interferon-induced protein with tetratricopeptide repeats 3 | NM_001549 | 204747_at | 985.04 | 61.94 | -15.90 | 0.00031 | <0.01 | 17.23 | -57.16 | 0.00023 | <0.01 |
| Interferon-induced protein with tetratricopeptide repeats 5 | N47725 | 203595_s_at | 187.98 | 60.03 | -3.13 | 0.000002 | <0.01 | 37.50 | -5.01 | 0.000002 | 0.0096 |
| Interleukin 1 receptor, type I | NM_000877 | 202948_at | 1273.37 | 429.19 | -2.97 | 0.000052 | <0.01 | 256.57 | -4.96 | 0.000011 | <0.01 |
| Interleukin 15 receptor, alpha | NM_002189 | 207375_s_at | 170.29 | 19.41 | -8.77 | 0.00057 | <0.01 | 8.56 | -19.89 | 0.00035 | 0.049 |
| Interleukin 6 signal transducer (gp130) | BE856546 | 204863_s_at | 786.04 | 179.76 | -4.37 | 0.001 | <0.01 | 175.64 | -4.48 | 0.001 | 0.016 |
| Interleukin 6 signal transducer (gp130) | NM_002184 | 204864_s_at | 144.41 | 41.77 | -3.46 | 0.0012 | <0.01 | 30.96 | -4.66 | 0.0006 | 0.073 |
| Interleukin 6 signal transducer (gp130) | AB015706 | 211000_s_at | 529.90 | 108.08 | -4.90 | 0.0043 | <0.01 | 113.03 | -4.69 | 0.0046 | 0.049 |
| Interleukin 6 signal transducer (gp130) | AL049265 | 212195_at | 2080.57 | 725.27 | -2.87 | 0.00019 | <0.01 | 712.11 | -2.92 | 0.00023 | <0.01 |
| Interleukin 6 signal transducer (gp130) | AW242916 | 212196_at | 813.69 | 186.59 | -4.36 | 0.0039 | <0.01 | 202.69 | -4.01 | 0.0046 | 0.049 |
| Interleukin 7 receptor | NM_002185 | 205798_at | 484.14 | 174.18 | -2.78 | 0.019 | <0.01 | 57.81 | -8.37 | 0.0028 | 0.049 |
| Interleukin 8 | NM_000584 | 202859_x_at | 1540.90 | 656.27 | -2.35 | 0.00059 | <0.01 | 118.65 | -12.99 | 0.000007 | <0.01 |
| Interleukin 8 | AF043337 | 211506_s_at | 799.00 | 324.04 | -2.47 | 0.0032 | <0.01 | 57.29 | -13.95 | 0.00013 | <0.01 |
| ISG15 ubiquitin-like modifier | NM_005101 | 205483_s_at | 3787.75 | 411.64 | -9.20 | <0.000001 | <0.01 | 236.12 | -16.04 | 0.000001 | <0.01 |
| Jun proto-oncogene | BG491844 | 201464_x_at | 263.93 | 163.16 | -1.62 | 0.0039 | 0.012 | 113.87 | -2.32 | 0.00029 | 0.028 |
| Jun proto-oncogene | NM_002228 | 201466_s_at | 188.13 | 92.31 | -2.04 | 0.00001 | 0.094 | 63.30 | -2.97 | 0.000002 | 0.028 |
| KIAA1199 | AB033025 | 212942_s_at | 799.03 | 144.95 | -5.51 | 0.00071 | <0.01 | 30.75 | -25.99 | 0.00027 | <0.01 |
| Kruppel-like factor 4 (gut) | BF514079 | 221841_s_at | 234.23 | 78.33 | -2.99 | 0.000036 | <0.01 | 75.85 | -3.09 | 0.005 | 0.016 |
| Lamin A/C | NM_005572 | 203411_s_at | 692.04 | 574.53 | -1.20 | 0.49 | 0.048 | 266.25 | -2.60 | 0.0056 | 0.049 |
| Lamin A/C | AK026584 | 212086_x_at | 554.94 | 430.48 | -1.29 | 0.12 | 0.048 | 236.44 | -2.35 | 0.00014 | 0.0096 |
| Lamin A/C | AA063189 | 214213_x_at | 277.44 | 186.48 | -1.49 | 0.018 | 0.22 | 108.38 | -2.56 | 0.000051 | 0.028 |
| Laminin, alpha 4 | NM_002290 | 202202_s_at | 741.79 | 306.34 | -2.42 | 0.000034 | <0.01 | 362.24 | -2.05 | 0.0026 | 0.0096 |
|  |  |  |  | **SSc-ILD** | | | | **IPF** | | | |
| **Annotation** | **Accession** | **Probe Set ID** | **Control mean** | **Ssc-ILD mean** | **Fold change** | **p value** | **q-value(%)** | **IPF mean** | **Fold change** | **p value** | **q-value(%)** |
| Laminin, beta 1 | NM_002291 | 201505_at | 551.79 | 244.06 | -2.26 | 0.00036 | <0.01 | 290.12 | -1.90 | 0.0014 | 0.05 |
| Laminin, beta 1 | M20206 | 211651_s_at | 404.45 | 194.10 | -2.08 | 0.0026 | <0.01 | 212.10 | -1.91 | 0.0038 | 0.073 |
| Lanosterol synthase | AW084510 | 202245_at | 330.81 | 136.12 | -2.43 | 0.068 | 0.0303 | 49.93 | -6.63 | 0.0068 | 0.073 |
| Lectin, galactoside-binding, soluble, 3 | BC001120 | 208949_s_at | 2136.91 | 1196.25 | -1.79 | 0.00078 | <0.01 | 799.08 | -2.67 | <0.000001 | <0.01 |
| Lectin, galactoside-binding, soluble, 3 binding protein | NM_005567 | 200923_at | 506.23 | 154.97 | -3.27 | 0.00008 | <0.01 | 223.39 | -2.27 | 0.00067 | 0.028 |
| Leucine aminopeptidase 3 | NM_015907 | 217933_s_at | 705.29 | 136.52 | -5.17 | 0.0026 | <0.01 | 118.90 | -5.93 | 0.0021 | 0.028 |
| Lipase A, lysosomal acid, cholesterol esterase | NM_000235 | 201847_at | 278.49 | 142.14 | -1.96 | 0.049 | 0.0303 | 95.13 | -2.93 | 0.0083 | 0.073 |
| Lipin 1 | D80010 | 212276_at | 230.67 | 112.61 | -2.05 | 0.081 | 0.048 | 61.19 | -3.77 | 0.00086 | 0.049 |
| Lipoma HMGIC fusion partner | NM_005780 | 218656_s_at | 262.95 | 114.35 | -2.30 | 0.0029 | <0.01 | 95.54 | -2.75 | 0.0016 | 0.073 |
| Low density lipoprotein receptor-related protein associated protein 1 | NM_002337 | 201186_at | 384.20 | 254.17 | -1.51 | 0.0025 | <0.01 | 170.32 | -2.26 | 0.000001 | <0.01 |
| Lumican | NM_002345 | 201744_s_at | 1252.91 | 411.48 | -3.04 | 0.000009 | <0.01 | 301.16 | -4.16 | 0.000046 | <0.01 |
| Lymphocyte antigen 6 complex, locus E | NM_002346 | 202145_at | 966.94 | 177.81 | -5.44 | 0.000045 | <0.01 | 66.98 | -14.44 | 0.000023 | <0.01 |
| Lymphocyte antigen 96 | NM_015364 | 206584_at | 229.75 | 95.62 | -2.40 | 0.00031 | <0.01 | 94.24 | -2.44 | 0.00044 | 0.049 |
| Lysophosphatidic acid receptor 1 | AW269335 | 204036_at | 635.12 | 273.16 | -2.33 | 0.0026 | <0.01 | 146.54 | -4.33 | 0.000089 | <0.01 |
| Lysophosphatidic acid receptor 1 | BF055366 | 204037_at | 543.76 | 244.66 | -2.22 | 0.0075 | <0.01 | 139.80 | -3.89 | 0.00028 | 0.0096 |
| Lysosomal-associated membrane protein 1 | J03263 | 201551_s_at | 265.56 | 189.68 | -1.40 | 0.26 | 0.41 | 95.01 | -2.80 | 0.0093 | 0.13 |
| Major histocompatibility complex, class I, A | AI923492 | 213932_x_at | 2612.13 | 1102.23 | -2.37 | 0.000015 | <0.01 | 808.85 | -3.23 | 0.000002 | <0.01 |
| Major histocompatibility complex, class I, A | AA573862 | 215313_x_at | 2751.38 | 1110.07 | -2.48 | 0.000006 | <0.01 | 813.32 | -3.38 | 0.000003 | <0.01 |
| Major histocompatibility complex, class I, A | M80469 | 217436_x_at | 773.14 | 253.30 | -3.05 | 0.00048 | <0.01 | 187.76 | -4.12 | 0.00025 | <0.01 |
| Major histocompatibility complex, class I, B | D83043 | 208729_x_at | 2374.26 | 805.76 | -2.95 | 0.00028 | <0.01 | 352.03 | -6.74 | 0.000033 | <0.01 |
| Major histocompatibility complex, class I, B | L42024 | 209140_x_at | 3231.85 | 1163.91 | -2.78 | 0.00025 | <0.01 | 400.17 | -8.08 | 0.000006 | <0.01 |
| Major histocompatibility complex, class I, B | L07950 | 211911_x_at | 2548.47 | 813.48 | -3.13 | 0.00035 | <0.01 | 365.95 | -6.96 | 0.000071 | <0.01 |
| Major histocompatibility complex, class I, C | BC004489 | 208812_x_at | 2623.61 | 898.42 | -2.92 | 0.00035 | <0.01 | 430.89 | -6.09 | 0.000032 | <0.01 |
| Major histocompatibility complex, class I, C | U62824 | 211799_x_at | 1112.53 | 323.28 | -3.44 | 0.00055 | <0.01 | 175.22 | -6.35 | 0.00019 | <0.01 |
| Major histocompatibility complex, class I, C | M12679 | 214459_x_at | 2813.68 | 932.88 | -3.02 | 0.000061 | <0.01 | 462.02 | -6.09 | 0.000013 | <0.01 |
| Major histocompatibility complex, class I, C | AK024836 | 216526_x_at | 3043.48 | 964.14 | -3.16 | 0.00011 | <0.01 | 424.40 | -7.17 | 0.000015 | <0.01 |
| Major histocompatibility complex, class I, E | X56841 | 200904_at | 537.54 | 125.18 | -4.29 | 0.000002 | <0.01 | 59.31 | -9.06 | 0.000002 | <0.01 |
| Major histocompatibility complex, class I, E | NM_005516 | 200905_x_at | 1414.20 | 442.92 | -3.19 | 0.000025 | <0.01 | 176.71 | -8.00 | 0.000007 | <0.01 |
| Major histocompatibility complex, class I, E | M31183 | 217456_x_at | 581.41 | 164.58 | -3.53 | 0.00012 | <0.01 | 85.27 | -6.82 | 0.000047 | <0.01 |
| Major histocompatibility complex, class I, F | NM_018950 | 204806_x_at | 1239.65 | 396.69 | -3.12 | 0.000063 | <0.01 | 240.54 | -5.15 | 0.000028 | <0.01 |
| Major histocompatibility complex, class I, F | AW514210 | 221875_x_at | 1272.83 | 378.69 | -3.36 | 0.00021 | <0.01 | 230.57 | -5.52 | 0.000093 | <0.01 |
| Major histocompatibility complex, class I, G | AF226990 | 210514_x_at | 1406.95 | 368.96 | -3.81 | 0.00012 | <0.01 | 233.25 | -6.03 | 0.000063 | <0.01 |
| Major histocompatibility complex, class I, G | M90685 | 211528_x_at | 1715.65 | 482.96 | -3.55 | 0.00039 | <0.01 | 355.11 | -4.83 | 0.00024 | <0.01 |
| Major histocompatibility complex, class I, G | M90684 | 211529_x_at | 1596.04 | 434.44 | -3.67 | 0.00012 | <0.01 | 303.29 | -5.26 | 0.000073 | <0.01 |
| Major histocompatibility complex, class I, G | M90686 | 211530_x_at | 1322.04 | 447.38 | -2.96 | 0.001 | <0.01 | 258.12 | -5.12 | 0.00019 | <0.01 |
| Mannosidase, alpha, class 1A, member 1 | BG287153 | 221760_at | 236.85 | 78.42 | -3.02 | 0.00051 | <0.01 | 63.74 | -3.72 | 0.0011 | 0.049 |
| Matrix-remodelling associated 5 | AF245505 | 209596_at | 672.60 | 257.82 | -2.61 | 0.0016 | <0.01 | 256.54 | -2.62 | 0.00016 | <0.01 |
| MAX interactor 1 | NM_005962 | 202364_at | 176.51 | 121.93 | -1.45 | 0.054 | 0.41 | 65.85 | -2.68 | 0.00025 | 0.049 |
| Meis homeobox 3 pseudogene 1 | H15129 | 214077_x_at | 185.97 | 76.37 | -2.44 | 0.0041 | 0.012 | 65.27 | -2.85 | 0.0024 | 0.073 |
| Membrane metallo-endopeptidase | AI433463 | 203434_s_at | 559.71 | 189.78 | -2.95 | 0.058 | 0.022 | 10.52 | -53.20 | 0.0068 | 0.049 |
| Membrane metallo-endopeptidase | NM_007287 | 203435_s_at | 660.90 | 215.48 | -3.07 | 0.074 | 0.022 | 15.48 | -42.70 | 0.012 | 0.073 |
| Methyltransferase like 7A | NM_014033 | 207761_s_at | 219.19 | 59.15 | -3.71 | 0.0018 | <0.01 | 55.35 | -3.96 | 0.0016 | 0.073 |
| Microfibrillar-associated protein 4 | R72286 | 212713_at | 578.43 | 204.89 | -2.82 | 0.00087 | <0.01 | 363.37 | -1.59 | 0.036 | 0.13 |
| Midline 1 | NM_000381 | 203637_s_at | 153.47 | 86.66 | -1.77 | 0.029 | 0.401 | 52.31 | -2.93 | 0.0038 | 0.13 |
|  |  |  |  | **SSc-ILD** | | | | **IPF** | | | |
| **Annotation** | **Accession** | **Probe Set ID** | **Control mean** | **Ssc-ILD mean** | **Fold change** | **p value** | **q-value(%)** | **IPF mean** | **Fold change** | **p value** | **q-value(%)** |
| Mitogen-activated protein kinase kinase kinase 5 | D84476 | 203836_s_at | 158.06 | 40.42 | -3.91 | 0.00011 | <0.01 | 18.53 | -8.53 | 0.000016 | 0.028 |
| Mitogen-activated protein kinase kinase kinase 5 | NM_005923 | 203837_at | 174.28 | 44.40 | -3.93 | 0.0017 | <0.01 | 17.26 | -10.10 | 0.00026 | 0.049 |
| Monooxygenase, DBH-like 1 | AY007239 | 209708_at | 248.08 | 104.05 | -2.38 | 0.0032 | <0.01 | 26.04 | -9.53 | 0.000025 | 0.0096 |
| Myeloid differentiation primary response gene (88) | U70451 | 209124_at | 262.90 | 106.41 | -2.47 | 0.00024 | <0.01 | 96.30 | -2.73 | 0.0038 | 0.049 |
| Myristoylated alanine-rich protein kinase C substrate | AW163148 | 201668_x_at | 215.14 | 195.52 | -1.10 | 0.58 | 0.41 | 95.82 | -2.25 | 0.00033 | 0.073 |
| Myxovirus resistance 1, interferon-inducible protein p78 (mouse) | NM_002462 | 202086_at | 1361.89 | 91.23 | -14.93 | 0.00012 | <0.01 | 8.84 | -154.06 | 0.000096 | <0.01 |
| Myxovirus resistance 2 (mouse) | NM_002463 | 204994_at | 517.28 | 40.63 | -12.73 | 0.00089 | <0.01 | 8.54 | -60.57 | 0.00062 | 0.0096 |
| N-acetylglucosamine-1-phosphate transferase, alpha and beta subunits | AK001821 | 212959_s_at | 209.96 | 84.77 | -2.48 | 0.0034 | <0.01 | 139.94 | -1.50 | 0.08 | 0.29 |
| N-acylsphingosine amidohydrolase (acid ceramidase) 1 | U47674 | 210980_s_at | 458.82 | 272.19 | -1.69 | 0.0095 | <0.01 | 226.96 | -2.02 | 0.0008 | 0.049 |
| N-acylsphingosine amidohydrolase (acid ceramidase) 1 | AI934569 | 213702_x_at | 539.00 | 313.19 | -1.72 | 0.0034 | <0.01 | 255.58 | -2.11 | 0.001 | 0.016 |
| NAD(P)H dehydrogenase, quinone 1 | AI039874 | 201467_s_at | 144.96 | 139.18 | -1.04 | 0.85 | 0.41 | 23.26 | -6.23 | 0.00033 | 0.073 |
| NAD(P)H dehydrogenase, quinone 1 | NM_000903 | 201468_s_at | 433.15 | 294.94 | -1.47 | 0.11 | 0.048 | 84.60 | -5.12 | 0.00052 | 0.016 |
| NAD(P)H dehydrogenase, quinone 1 | BC000906 | 210519_s_at | 467.99 | 406.36 | -1.15 | 0.51 | 0.41 | 108.34 | -4.32 | 0.00036 | 0.016 |
| NECAP endocytosis associated 2 | NM_018090 | 220731_s_at | 252.02 | 169.73 | -1.48 | 0.00037 | 0.18 | 117.71 | -2.14 | 0.0016 | 0.016 |
| Neuropilin 1 | BE620457 | 212298_at | 365.60 | 194.79 | -1.88 | 0.00046 | <0.01 | 116.93 | -3.13 | 0.00016 | <0.01 |
| Neutral sphingomyelinase activation associated factor | NM_003580 | 203269_at | 203.12 | 136.38 | -1.49 | 0.13 | 0.41 | 70.61 | -2.88 | 0.0015 | 0.073 |
| Nicotinamide phosphoribosyltransferase | BF575514 | 217738_at | 330.31 | 136.38 | -2.42 | 0.0017 | <0.01 | 68.29 | -4.84 | 0.000055 | 0.0096 |
| Nicotinamide phosphoribosyltransferase | NM_005746 | 217739_s_at | 541.20 | 202.44 | -2.67 | 0.00068 | <0.01 | 116.11 | -4.66 | 0.00013 | <0.01 |
| Ninjurin 1 | NM_004148 | 203045_at | 221.58 | 96.20 | -2.30 | 0.000038 | <0.01 | 101.96 | -2.17 | 0.0019 | 0.028 |
| N-myc (and STAT) interactor | NM_004688 | 203964_at | 307.52 | 72.41 | -4.25 | 0.000068 | <0.01 | 70.23 | -4.38 | 0.000072 | 0.016 |
| N-sulfoglucosamine sulfohydrolase | U30894 | 35626_at | 252.62 | 144.04 | -1.75 | 0.077 | 0.26 | 91.12 | -2.77 | 0.0074 | 0.073 |
| Nuclear factor of kappa light polypeptide gene enhancer in B-cells inhibitor, alpha | AI078167 | 201502_s_at | 695.42 | 211.57 | -3.29 | 0.00001 | <0.01 | 41.58 | -16.73 | 0.000002 | <0.01 |
| Nuclear factor, interleukin 3 regulated | NM_005384 | 203574_at | 214.89 | 154.71 | -1.39 | 0.17 | 0.41 | 97.87 | -2.20 | 0.0015 | 0.073 |
| Nuclear pore complex interacting protein | NM_006985 | 204538_x_at | 495.73 | 291.31 | -1.70 | 0.035 | 0.012 | 235.72 | -2.10 | 0.00042 | 0.028 |
| Nuclear pore complex interacting protein | AC002045 | 214870_x_at | 531.77 | 300.70 | -1.77 | 0.0077 | <0.01 | 258.68 | -2.06 | 0.00024 | 0.016 |
| Nucleosome assembly protein 1-like 1 | AW148801 | 212967_x_at | 752.40 | 637.16 | -1.18 | 0.28 | 0.048 | 347.59 | -2.16 | 0.00012 | <0.01 |
| Nucleosome assembly protein 1-like 1 | AI985751 | 213864_s_at | 846.45 | 723.03 | -1.17 | 0.32 | 0.048 | 409.93 | -2.06 | 0.00038 | 0.0096 |
| Odd-skipped related 2 (Drosophila) | AI811298 | 213568_at | 144.37 | 36.28 | -3.98 | 0.00043 | <0.01 | 12.36 | -11.68 | 0.00012 | 0.049 |
| Olfactomedin-like 1 | AW305097 | 217525_at | 149.66 | 32.98 | -4.54 | 0.0011 | <0.01 | 22.02 | -6.80 | 0.00065 | 0.073 |
| Olfactomedin-like 3 | NM_020190 | 218162_at | 320.26 | 223.75 | -1.43 | 0.16 | 0.34 | 83.73 | -3.82 | 0.00025 | 0.028 |
| Optineurin | AV757675 | 202073_at | 193.33 | 75.56 | -2.56 | 0.00018 | <0.01 | 58.76 | -3.29 | 0.00019 | 0.028 |
| Optineurin | NM_021980 | 202074_s_at | 573.06 | 279.49 | -2.05 | 0.00004 | <0.01 | 191.17 | -3.00 | <0.000001 | <0.01 |
| Oxysterol binding protein-like 8 | AL049923 | 212582_at | 980.40 | 573.63 | -1.71 | 0.019 | <0.01 | 448.16 | -2.19 | 0.0023 | 0.028 |
| PDZ domain containing ring finger 3 | AL569804 | 212915_at | 193.21 | 91.88 | -2.10 | 0.000079 | <0.01 | 56.45 | -3.42 | 0.00015 | 0.028 |
| Pentraxin 3, long | NM_002852 | 206157_at | 1415.20 | 202.98 | -6.97 | 0.000019 | <0.01 | 35.23 | -40.17 | 0.000016 | <0.01 |
| Peptidylglycine alpha-amidating monooxygenase | AI022882 | 212958_x_at | 692.83 | 424.22 | -1.63 | 0.043 | 0.012 | 327.42 | -2.12 | 0.0032 | 0.049 |
| Perilipin 2 | BC005127 | 209122_at | 477.98 | 226.55 | -2.11 | 0.00012 | <0.01 | 122.44 | -3.90 | 0.000004 | <0.01 |
| Peripheral myelin protein 22 | L03203 | 210139_s_at | 1142.20 | 378.29 | -3.02 | 0.000086 | <0.01 | 272.08 | -4.20 | 0.000042 | <0.01 |
| PHD finger protein 11 | BF055474 | 221816_s_at | 379.52 | 127.00 | -2.99 | 0.000022 | <0.01 | 79.05 | -4.80 | 0.000011 | <0.01 |
| PHD finger protein 15 | AI735639 | 212660_at | 141.20 | 44.37 | -3.18 | 0.000202 | 0.14 | 40.62 | -3.48 | 0.0002 | 0.073 |
| Phorbol-12-myristate-13-acetate-induced protein 1 | AI857639 | 204285_s_at | 420.65 | 176.48 | -2.38 | 0.000026 | <0.01 | 83.62 | -5.03 | 0.000003 | <0.01 |
| Phosphatidic acid phosphatase type 2A | AB000888 | 209147_s_at | 454.68 | 174.69 | -2.60 | 0.0014 | <0.01 | 197.27 | -2.30 | 0.0026 | 0.049 |
|  |  |  |  | **SSc-ILD** | | | | **IPF** | | | |
| **Annotation** | **Accession** | **Probe Set ID** | **Control mean** | **Ssc-ILD mean** | **Fold change** | **p value** | **q-value(%)** | **IPF mean** | **Fold change** | **p value** | **q-value(%)** |
| Phosphatidic acid phosphatase type 2A | AF014403 | 210946_at | 662.73 | 320.62 | -2.07 | 0.00039 | <0.01 | 330.84 | -2.00 | 0.0006 | 0.016 |
| Phosphatidic acid phosphatase type 2B | AA628586 | 212226_s_at | 2495.93 | 536.86 | -4.65 | 0.000011 | <0.01 | 198.04 | -12.60 | 0.000006 | <0.01 |
| Phosphatidic acid phosphatase type 2B | AV725664 | 212230_at | 1344.37 | 296.77 | -4.53 | 0.000007 | <0.01 | 119.05 | -11.29 | 0.000005 | <0.01 |
| Phosphatidylethanolamine binding protein 1 | AF130103 | 210825_s_at | 529.37 | 455.13 | -1.16 | 0.70 | 0.41 | 195.02 | -2.71 | 0.028 | 0.13 |
| Phosphodiesterase 5A, cGMP-specific | NM_001083 | 206757_at | 137.12 | 27.99 | -4.90 | 0.018 | 0.022 | 21.90 | -6.26 | 0.014 | 0.13 |
| Phosphogluconate dehydrogenase | NM_002631 | 201118_at | 192.00 | 169.38 | -1.13 | 0.50 | 0.41 | 88.71 | -2.16 | 0.00071 | 0.073 |
| Phospholipid scramblase 1 | NM_021105 | 202430_s_at | 470.63 | 74.34 | -6.33 | 0.00003 | <0.01 | 30.49 | -15.44 | 0.00002 | <0.01 |
| Phospholipid scramblase 1 | AI825926 | 202446_s_at | 746.01 | 134.49 | -5.55 | 0.000036 | <0.01 | 43.64 | -17.09 | 0.000019 | <0.01 |
| Plasminogen activator, tissue | NM_000930 | 201860_s_at | 325.73 | 257.22 | -1.27 | 0.68 | 0.41 | 72.67 | -4.48 | 0.0054 | 0.073 |
| Platelet derived growth factor D | NM_025208 | 219304_s_at | 173.66 | 43.11 | -4.03 | 0.0016 | <0.01 | 59.60 | -2.91 | 0.0039 | 0.13 |
| Platelet-derived growth factor receptor, alpha polypeptide | NM_006206 | 203131_at | 2022.22 | 840.14 | -2.41 | 0.000003 | <0.01 | 758.23 | -2.67 | <0.000001 | <0.01 |
| Pleckstrin homology domain containing, family A member 5 | NM_019012 | 220952_s_at | 179.45 | 107.24 | -1.67 | 0.013 | 0.29 | 75.54 | -2.38 | 0.00021 | 0.073 |
| Pleckstrin homology domain containing, family O member 2 | NM_025201 | 204436_at | 184.73 | 64.71 | -2.85 | 0.00082 | <0.01 | 77.32 | -2.39 | 0.0018 | 0.13 |
| Pleckstrin homology-like domain, family A, member 1 | AA576961 | 217996_at | 275.83 | 236.03 | -1.17 | 0.72 | 0.41 | 128.03 | -2.15 | 0.0063 | 0.073 |
| Pleckstrin homology-like domain, family A, member 1 | AI795908 | 217997_at | 215.62 | 160.36 | -1.34 | 0.45 | 0.41 | 69.62 | -3.10 | 0.0068 | 0.13 |
| Pleckstrin homology-like domain, family A, member 1 | NM_007350 | 217998_at | 163.03 | 117.28 | -1.39 | 0.39 | 0.41 | 52.03 | -3.13 | 0.00041 | 0.073 |
| Pleiotrophin | AL565812 | 209465_x_at | 199.49 | 64.80 | -3.08 | 0.0015 | <0.01 | 106.97 | -1.86 | 0.014 | 0.26 |
| Pleiotrophin | M57399 | 209466_x_at | 688.02 | 232.60 | -2.96 | 0.00066 | <0.01 | 266.06 | -2.59 | 0.0013 | 0.028 |
| Pleiotrophin | BC005916 | 211737_x_at | 676.08 | 225.25 | -3.00 | 0.0017 | <0.01 | 247.10 | -2.74 | 0.0024 | 0.028 |
| Plexin C1 | AF035307 | 213241_at | 174.48 | 69.66 | -2.50 | 0.058 | 0.048 | 7.50 | -23.26 | 0.0025 | 0.073 |
| Poly (ADP-ribose) polymerase family, member 12 | NM_022750 | 218543_s_at | 213.75 | 39.08 | -5.47 | 0.000033 | <0.01 | 18.98 | -11.26 | 0.000024 | 0.0096 |
| Potassium channel tetramerisation domain containing 12 | AA551075 | 212188_at | 395.76 | 153.64 | -2.58 | 0.016 | <0.01 | 83.99 | -4.71 | 0.004 | 0.049 |
| Potassium channel tetramerisation domain containing 12 | AI718937 | 212192_at | 246.53 | 71.10 | -3.47 | 0.011 | <0.01 | 58.37 | -4.22 | 0.0079 | 0.073 |
| Potassium inwardly-rectifying channel, subfamily J, member 2 | AF153820 | 206765_at | 114.80 | 25.23 | -4.55 | 0.00029 | 0.14 | 12.71 | -9.03 | 0.000067 | 0.073 |
| Pregnancy specific beta-1-glycoprotein 3 | R32065 | 215821_x_at | 140.58 | 164.18 | 1.17 | 0.68 | 0.56 | 12.04 | -11.68 | 0.0048 | 0.13 |
| Pregnancy specific beta-1-glycoprotein 4 | NM_002780 | 208191_x_at | 139.05 | 110.09 | -1.26 | 0.59 | 0.41 | 14.20 | -9.79 | 0.0038 | 0.13 |
| Prion protein | NM_000311 | 201300_s_at | 1026.41 | 568.73 | -1.80 | 0.000013 | <0.01 | 365.99 | -2.80 | 0.00025 | <0.01 |
| Prion protein | AV725328 | 215707_s_at | 229.58 | 135.62 | -1.69 | 0.0019 | 0.18 | 85.20 | -2.69 | 0.000048 | 0.028 |
| Progesterone receptor membrane component 1 | AL547946 | 201120_s_at | 679.10 | 438.16 | -1.55 | 0.021 | <0.01 | 278.14 | -2.44 | 0.0006 | 0.016 |
| Proline-rich nuclear receptor coactivator 1 | AF279899 | 209034_at | 289.73 | 149.27 | -1.94 | 0.000705 | <0.01 | 103.86 | -2.79 | 0.000008 | 0.0096 |
| Prosaposin | M32221 | 200866_s_at | 466.46 | 237.61 | -1.96 | 0.00024 | <0.01 | 197.59 | -2.36 | 0.000072 | 0.0096 |
| Prosaposin | NM_002778 | 200871_s_at | 1091.09 | 551.41 | -1.98 | 0.00054 | <0.01 | 466.83 | -2.34 | 0.000074 | <0.01 |
| Prostaglandin D2 synthase 21kDa (brain) | BC005939 | 211748_x_at | 209.41 | 85.49 | -2.45 | 0.034 | 0.0303 | 137.21 | -1.53 | 0.14 | 0.29 |
| Prostaglandin D2 synthase 21kDa (brain) | NM_000954 | 212187_x_at | 229.64 | 109.74 | -2.09 | 0.049 | 0.0303 | 135.74 | -1.69 | 0.038 | 0.29 |
| Prostaglandin E synthase | AF010316 | 210367_s_at | 214.48 | 67.85 | -3.16 | 0.00068 | <0.01 | 46.32 | -4.63 | 0.0003 | 0.049 |
| Prostaglandin I2 (prostacyclin) synthase | NM_000961 | 208131_s_at | 212.13 | 67.48 | -3.14 | 0.0036 | <0.01 | 28.41 | -7.47 | 0.000087 | 0.028 |
| Proteasome activator subunit 1 | NM_006263 | 200814_at | 684.77 | 247.47 | -2.77 | 0.000009 | <0.01 | 158.93 | -4.31 | 0.000005 | <0.01 |
| Proteasome activator subunit 2 | NM_002818 | 201762_s_at | 802.34 | 337.56 | -2.38 | 0.000041 | <0.01 | 290.05 | -2.77 | 0.000041 | <0.01 |
| Proteasome subunit, beta type, 10 | NM_002801 | 202659_at | 134.84 | 45.09 | -2.99 | 0.000009 | 0.14 | 22.69 | -5.94 | 0.000004 | 0.028 |
| Proteasome subunit, beta type, 8 | U17496 | 209040_s_at | 222.36 | 64.70 | -3.44 | 0.00013 | <0.01 | 16.70 | -13.31 | 0.000023 | 0.0096 |
| Proteasome subunit, beta type, 9 | NM_002800 | 204279_at | 395.20 | 51.45 | -7.68 | 0.0016 | <0.01 | 6.19 | -63.88 | 0.00072 | 0.016 |
| Protein kinase inhibitor gamma | NM_007066 | 202732_at | 560.87 | 211.19 | -2.66 | 0.000008 | <0.01 | 215.97 | -2.60 | 0.000023 | <0.01 |
| Protocadherin gamma subfamily A, 1 | NM_002588 | 205717_x_at | 333.10 | 210.97 | -1.58 | 0.074 | 0.048 | 126.89 | -2.63 | 0.00033 | 0.028 |
| Protocadherin gamma subfamily A, 1 | AF152318 | 209079_x_at | 353.06 | 221.20 | -1.60 | 0.07 | 0.048 | 140.35 | -2.52 | 0.0016 | 0.049 |
|  |  |  |  | **SSc-ILD** | | | | **IPF** | | | |
| **Annotation** | **Accession** | **Probe Set ID** | **Control mean** | **Ssc-ILD mean** | **Fold change** | **p value** | **q-value(%)** | **IPF mean** | **Fold change** | **p value** | **q-value(%)** |
| Protocadherin gamma subfamily A, 1 | BC006439 | 211066_x_at | 412.79 | 259.99 | -1.59 | 0.041 | 0.022 | 169.17 | -2.44 | 0.00076 | 0.028 |
| Protocadherin gamma subfamily A, 1 | AK026188 | 215836_s_at | 368.84 | 240.48 | -1.53 | 0.13 | 0.048 | 140.84 | -2.62 | 0.0028 | 0.049 |
| Quiescin Q6 sulfhydryl oxidase 1 | NM_002826 | 201482_at | 1457.49 | 353.25 | -4.13 | 0.016 | <0.01 | 571.25 | -2.55 | 0.04 | 0.13 |
| RAB13, member RAS oncogene family | NM_002870 | 202252_at | 1611.73 | 907.88 | -1.78 | 0.000079 | <0.01 | 741.81 | -2.17 | 0.000002 | <0.01 |
| RAB31, member RAS oncogene family | BE789881 | 217762_s_at | 152.89 | 125.54 | -1.22 | 0.41 | 0.41 | 47.99 | -3.19 | 0.00071 | 0.073 |
| RAB31, member RAS oncogene family | NM_006868 | 217763_s_at | 209.89 | 189.70 | -1.11 | 0.63 | 0.41 | 83.33 | -2.52 | 0.0012 | 0.073 |
| RAB31, member RAS oncogene family | AF183421 | 217764_s_at | 244.54 | 194.36 | -1.26 | 0.22 | 0.41 | 84.16 | -2.91 | 0.00004 | 0.028 |
| RAB9A, member RAS oncogene family | NM_004251 | 221808_at | 256.52 | 147.90 | -1.73 | 0.0016 | <0.01 | 98.02 | -2.62 | 0.000038 | 0.028 |
| Radical S-adenosyl methionine domain containing 2 | AI337069 | 213797_at | 333.76 | 13.28 | -25.14 | 0.0039 | <0.01 | 6.11 | -54.60 | 0.0034 | 0.049 |
| RanBP-type and C3HC4-type zinc finger containing 1 | BE788439 | 221827_at | 376.50 | 203.26 | -1.85 | 0.00062 | <0.01 | 122.74 | -3.07 | 0.000026 | 0.0096 |
| Ras homolog gene family, member Q | BF978689 | 212117_at | 244.24 | 178.60 | -1.37 | 0.077 | 0.401 | 88.80 | -2.75 | 0.000003 | <0.01 |
| Ras homolog gene family, member Q | BF670447 | 212119_at | 546.67 | 440.83 | -1.24 | 0.25 | 0.048 | 214.53 | -2.55 | 0.000012 | <0.01 |
| Ras homolog gene family, member Q | BE897886 | 212120_at | 494.23 | 353.81 | -1.40 | 0.046 | 0.022 | 218.22 | -2.26 | 0.000024 | <0.01 |
| Receptor (chemosensory) transporter protein 4 | NM_022147 | 219684_at | 196.44 | 13.26 | -14.82 | 0.000091 | <0.01 | 4.75 | -41.39 | 0.000075 | 0.016 |
| Receptor accessory protein 5 | BC000232 | 208873_s_at | 358.34 | 177.89 | -2.01 | 0.0024 | <0.01 | 257.34 | -1.39 | 0.047 | 0.29 |
| Receptor-interacting serine-threonine kinase 2 | AF064824 | 209545_s_at | 211.64 | 135.84 | -1.56 | 0.012 | 0.29 | 100.62 | -2.10 | 0.0011 | 0.073 |
| Regulator of calcineurin 2 | NM_005822 | 203498_at | 142.46 | 25.74 | -5.53 | 0.00039 | <0.01 | 8.58 | -16.60 | 0.00018 | 0.049 |
| Related RAS viral (r-ras) oncogene homolog 2 | AI753792 | 212589_at | 314.11 | 154.21 | -2.04 | 0.0011 | <0.01 | 118.32 | -2.65 | 0.00085 | 0.028 |
| Related RAS viral (r-ras) oncogene homolog 2 | AI431643 | 212590_at | 325.52 | 207.60 | -1.57 | 0.046 | 0.0303 | 162.44 | -2.00 | 0.00094 | 0.049 |
| Reticulocalbin 1, EF-hand calcium binding domain | NM_002901 | 201063_at | 651.08 | 439.95 | -1.48 | 0.031 | 0.012 | 297.52 | -2.19 | 0.00024 | 0.0096 |
| Retinoic acid receptor responder (tazarotene induced) 1 | NM_002888 | 206392_s_at | 253.84 | 27.91 | -9.09 | 0.044 | 0.022 | 32.28 | -7.86 | 0.047 | 0.22 |
| Retinoic acid receptor responder (tazarotene induced) 1 | AI669229 | 221872_at | 221.36 | 28.12 | -7.87 | 0.046 | 0.022 | 40.15 | -5.51 | 0.058 | 0.22 |
| Retinoic acid receptor responder (tazarotene induced) 2 | BC000069 | 209496_at | 450.34 | 43.85 | -10.27 | 0.01 | <0.01 | 189.72 | -2.37 | 0.071 | 0.22 |
| Retinoic acid receptor responder (tazarotene induced) 3 | NM_004585 | 204070_at | 239.43 | 17.24 | -13.89 | 0.00024 | <0.01 | 20.98 | -11.41 | 0.00028 | 0.028 |
| REV3-like, catalytic subunit of DNA polymerase zeta (yeast) | NM_002912 | 208070_s_at | 168.81 | 68.18 | -2.48 | 0.0016 | 0.012 | 39.63 | -4.26 | 0.00016 | 0.049 |
| Rho family GTPase 3 | BG054844 | 212724_at | 2833.29 | 910.51 | -3.11 | <0.000001 | <0.01 | 796.91 | -3.56 | <0.000001 | <0.01 |
| Rho GTPase activating protein 29 | NM_004815 | 203910_at | 184.45 | 111.51 | -1.65 | 0.13 | 0.41 | 19.87 | -9.28 | 0.0016 | 0.073 |
| Rho-related BTB domain containing 3 | N21138 | 202975_s_at | 506.03 | 287.66 | -1.76 | 0.025 | 0.012 | 217.17 | -2.33 | 0.0041 | 0.049 |
| Rho-related BTB domain containing 3 | NM_014899 | 202976_s_at | 271.18 | 121.05 | -2.24 | 0.029 | 0.022 | 128.97 | -2.10 | 0.035 | 0.22 |
| Ring finger protein 130 | NM_018434 | 217865_at | 158.60 | 97.84 | -1.62 | 0.011 | 0.401 | 57.18 | -2.77 | 0.000033 | 0.073 |
| Runt-related transcription factor 1; translocated to, 1 | NM_004349 | 205529_s_at | 174.82 | 99.65 | -1.75 | 0.069 | 0.34 | 31.33 | -5.58 | 0.00079 | 0.073 |
| S100 calcium binding protein A10 | NM_002966 | 200872_at | 2190.92 | 1235.45 | -1.77 | 0.000081 | <0.01 | 614.43 | -3.57 | 0.000001 | <0.01 |
| S100 calcium binding protein A4 | NM_002961 | 203186_s_at | 1741.88 | 940.25 | -1.85 | 0.018 | <0.01 | 517.83 | -3.36 | 0.00042 | <0.01 |
| SAM and SH3 domain containing 1 | AK025495 | 213236_at | 225.16 | 104.68 | -2.15 | 0.0018 | <0.01 | 66.59 | -3.38 | 0.00016 | 0.049 |
| Sarcoglycan, epsilon | NM_003919 | 204688_at | 421.32 | 202.84 | -2.08 | 0.00062 | <0.01 | 124.87 | -3.37 | 0.000055 | <0.01 |
| Secernin 1 | NM_014766 | 201462_at | 379.49 | 300.28 | -1.26 | 0.39 | 0.41 | 144.89 | -2.62 | 0.0043 | 0.073 |
| Secreted and transmembrane 1 | BF939675 | 213716_s_at | 285.00 | 21.99 | -12.96 | 0.0012 | <0.01 | 3.57 | -79.83 | 0.0008 | 0.028 |
| Selenoprotein P, plasma, 1 | NM_005410 | 201427_s_at | 345.19 | 37.87 | -9.12 | 0.012 | <0.01 | 19.42 | -17.77 | 0.009 | 0.073 |
| Sequestosome 1 | NM_003900 | 201471_s_at | 1499.86 | 657.74 | -2.28 | 0.00019 | <0.01 | 306.84 | -4.89 | <0.000001 | <0.01 |
| Sequestosome 1 | N30649 | 213112_s_at | 120.89 | 44.73 | -2.70 | 0.056 | 0.34 | 8.14 | -14.86 | 0.0031 | 0.13 |
| Serglycin | J03223 | 201858_s_at | 550.00 | 151.08 | -3.64 | 0.018 | <0.01 | 458.32 | -1.20 | 0.52 | 0.29 |
| Serglycin | NM_002727 | 201859_at | 816.74 | 228.32 | -3.58 | 0.019 | <0.01 | 671.17 | -1.22 | 0.52 | 0.29 |
| Serine carboxypeptidase 1 | NM_021626 | 218217_at | 220.13 | 92.13 | -2.39 | 0.00026 | <0.01 | 75.56 | -2.91 | 0.00013 | 0.049 |
| Serpin peptidase inhibitor, clade F, member 1 | NM_002615 | 202283_at | 1165.35 | 463.93 | -2.51 | 0.0019 | <0.01 | 343.55 | -3.39 | 0.000085 | <0.01 |
|  |  |  |  | **SSc-ILD** | | | | **IPF** | | | |
| **Annotation** | **Accession** | **Probe Set ID** | **Control mean** | **Ssc-ILD mean** | **Fold change** | **p value** | **q-value(%)** | **IPF mean** | **Fold change** | **p value** | **q-value(%)** |
| Serpin peptidase inhibitor, clade G (C1 inhibitor), member 1 | NM_000062 | 200986_at | 416.16 | 86.54 | -4.81 | 0.00098 | <0.01 | 116.65 | -3.57 | 0.0018 | 0.049 |
| Signal transducer and activator of transcription 1, 91kDa | NM_007315 | 200887_s_at | 2028.99 | 561.13 | -3.62 | 0.000001 | <0.01 | 307.90 | -6.59 | 0.000001 | <0.01 |
| Signal transducer and activator of transcription 1, 91kDa | BC002704 | 209969_s_at | 327.32 | 44.57 | -7.34 | 0.00017 | <0.01 | 15.28 | -21.42 | 0.000097 | 0.0096 |
| Signal transducer and activator of transcription 6, interleukin-4 induced | BC004973 | 201331_s_at | 256.75 | 156.47 | -1.64 | 0.0052 | 0.012 | 97.49 | -2.63 | 0.000009 | 0.016 |
| Slit homolog 2 (Drosophila) | AF055585 | 209897_s_at | 244.95 | 88.95 | -2.75 | 0.011 | <0.01 | 30.48 | -8.04 | 0.0016 | 0.049 |
| SMAD family member 3 | NM_005902 | 205398_s_at | 149.72 | 43.66 | -3.43 | 0.00092 | <0.01 | 13.99 | -10.70 | 0.00012 | 0.049 |
| SMAD family member 3 | NM_015400 | 218284_at | 263.84 | 69.27 | -3.81 | 0.0058 | <0.01 | 40.02 | -6.59 | 0.0024 | 0.049 |
| Snail homolog 2 (Drosophila) | AI572079 | 213139_at | 316.26 | 299.77 | -1.05 | 0.75 | 0.41 | 141.65 | -2.23 | 0.000004 | 0.0096 |
| Solute carrier family 22, member 4 | NM_003059 | 205896_at | 128.13 | 63.85 | -2.01 | 0.026 | 0.401 | 16.03 | -7.99 | 0.00081 | 0.073 |
| Solute carrier family 39 (zinc transporter), member 14 | D31887 | 212110_at | 618.94 | 414.75 | -1.49 | 0.086 | 0.03 | 212.80 | -2.91 | 0.00054 | 0.016 |
| Solute carrier family 39 (zinc transporter), member 8 | AB040120 | 209267_s_at | 494.00 | 43.88 | -11.26 | 0.0031 | <0.01 | 6.68 | -73.95 | 0.0019 | 0.028 |
| Solute carrier family 39 (zinc transporter), member 8 | NM_022154 | 219869_s_at | 228.81 | 26.27 | -8.71 | 0.0054 | <0.01 | 7.72 | -29.63 | 0.0033 | 0.073 |
| Solute carrier family 43, member 3 | AI630178 | 213113_s_at | 142.69 | 56.04 | -2.55 | 0.0021 | 0.18 | 37.26 | -3.83 | 0.00051 | 0.073 |
| Solute carrier family 5, member 3 | AI867198 | 213164_at | 322.12 | 254.16 | -1.27 | 0.33 | 0.41 | 157.74 | -2.04 | 0.0055 | 0.073 |
| Solute carrier family 7, member 11 | AB040875 | 209921_at | 237.66 | 155.76 | -1.53 | 0.11 | 0.34 | 110.03 | -2.16 | 0.019 | 0.073 |
| SP100 nuclear antigen | NM_003113 | 202863_at | 183.83 | 79.27 | -2.32 | 0.000008 | <0.01 | 80.01 | -2.30 | 0.00015 | 0.049 |
| SP100 nuclear antigen | NM_003113 | 202864_s_at | 238.59 | 81.10 | -2.94 | 0.00048 | <0.01 | 69.53 | -3.43 | 0.00035 | 0.049 |
| SP100 nuclear antigen | U36501 | 210218_s_at | 198.71 | 55.26 | -3.60 | 0.0068 | <0.01 | 50.13 | -3.96 | 0.0056 | 0.073 |
| SP110 nuclear body protein | NM_004509 | 208012_x_at | 228.58 | 46.71 | -4.89 | 0.00042 | <0.01 | 27.29 | -8.38 | 0.00022 | 0.028 |
| SP110 nuclear body protein | AA969194 | 209761_s_at | 171.10 | 38.84 | -4.40 | 0.00061 | <0.01 | 33.02 | -5.18 | 0.00046 | 0.049 |
| SP110 nuclear body protein | AF280094 | 209762_x_at | 171.04 | 43.33 | -3.95 | 0.00019 | <0.01 | 26.06 | -6.56 | 0.000095 | 0.049 |
| Spermidine/spermine N1-acetyltransferase 1 | NM_002970 | 203455_s_at | 352.07 | 214.30 | -1.64 | 0.13 | 0.048 | 168.00 | -2.10 | 0.01 | 0.073 |
| Spermidine/spermine N1-acetyltransferase 1 | M55580 | 210592_s_at | 784.93 | 426.08 | -1.84 | 0.0047 | <0.01 | 294.57 | -2.66 | 0.0001 | <0.01 |
| Spermidine/spermine N1-acetyltransferase 1 | BE971383 | 213988_s_at | 250.38 | 151.17 | -1.66 | 0.15 | 0.29 | 99.35 | -2.52 | 0.00075 | 0.073 |
| Sphingomyelin phosphodiesterase, acid-like 3A | AA873600 | 213624_at | 253.49 | 76.42 | -3.32 | 0.018 | 0.012 | 88.13 | -2.88 | 0.027 | 0.13 |
| Spondin 2, extracellular matrix protein | NM_012445 | 218638_s_at | 634.94 | 226.78 | -2.80 | 0.021 | <0.01 | 344.52 | -1.84 | 0.035 | 0.13 |
| Stanniocalcin 2 | AI435828 | 203438_at | 621.16 | 257.79 | -2.41 | 0.0018 | <0.01 | 194.19 | -3.20 | 0.00053 | 0.016 |
| Stanniocalcin 2 | BC000658 | 203439_s_at | 385.62 | 256.64 | -1.50 | 0.018 | 0.022 | 189.63 | -2.03 | 0.00043 | 0.049 |
| Stomatin | AI537887 | 201060_x_at | 513.40 | 134.67 | -3.81 | 0.000024 | <0.01 | 82.30 | -6.24 | 0.000012 | <0.01 |
| Stomatin | M81635 | 201061_s_at | 851.55 | 198.97 | -4.28 | 0.00008 | <0.01 | 148.19 | -5.75 | 0.000067 | <0.01 |
| Sulfatase 1 | BE500977 | 212354_at | 601.25 | 283.95 | -2.12 | 0.011 | <0.01 | 365.73 | -1.64 | 0.057 | 0.13 |
| Sulfide quinone reductase-like (yeast) | NM_021199 | 217995_at | 530.98 | 156.96 | -3.38 | 0.000001 | <0.01 | 69.44 | -7.65 | 0.000001 | <0.01 |
| Superoxide dismutase 2, mitochondrial | W46388 | 215223_s_at | 1587.48 | 236.02 | -6.73 | <0.000001 | <0.01 | 35.80 | -44.35 | <0.000001 | <0.01 |
| Superoxide dismutase 2, mitochondrial | X15132 | 216841_s_at | 484.78 | 84.08 | -5.77 | 0.000018 | <0.01 | 19.61 | -24.72 | 0.000012 | <0.01 |
| Superoxide dismutase 2, mitochondrial | BF575213 | 221477_s_at | 2180.37 | 311.04 | -7.01 | <0.000001 | <0.01 | 29.80 | -73.17 | <0.000001 | <0.01 |
| Sushi, von Willebrand factor type A, EGF and pentraxin domain containing 1 | AA716107 | 213247_at | 332.92 | 61.79 | -5.39 | 0.0019 | <0.01 | 44.81 | -7.43 | 0.0013 | 0.028 |
| Sushi-repeat-containing protein, X-linked | NM_006307 | 204955_at | 447.36 | 336.17 | -1.33 | 0.17 | 0.048 | 125.51 | -3.56 | 0.00017 | 0.0096 |
| Syndecan binding protein (syntenin) | NM_005625 | 200958_s_at | 1404.06 | 669.35 | -2.10 | 0.000094 | <0.01 | 541.26 | -2.59 | 0.000013 | <0.01 |
| TAP binding protein (tapasin) | AF029750 | 208829_at | 270.49 | 76.68 | -3.53 | 0.000003 | <0.01 | 58.58 | -4.62 | 0.000005 | <0.01 |
| Tenascin XA pseudogene | NM_007116 | 206093_x_at | 174.54 | 36.65 | -4.76 | 0.044 | 0.0303 | 20.96 | -8.33 | 0.027 | 0.13 |
| Tenascin XA pseudogene | BE044614 | 213451_x_at | 176.99 | 33.03 | -5.36 | 0.045 | 0.0303 | 15.17 | -11.67 | 0.026 | 0.13 |
| Tenascin XA pseudogene | M25813 | 216333_x_at | 158.43 | 32.60 | -4.86 | 0.045 | 0.030 | 9.96 | -15.90 | 0.021 | 0.13 |
|  |  |  |  | **SSc-ILD** | | | | **IPF** | | | |
| **Annotation** | **Accession** | **Probe Set ID** | **Control mean** | **Ssc-ILD mean** | **Fold change** | **p value** | **q-value(%)** | **IPF mean** | **Fold change** | **p value** | **q-value(%)** |
| Tensin 3 | NM_022748 | 217853_at | 756.79 | 307.08 | -2.46 | 0.0013 | <0.01 | 278.88 | -2.71 | 0.00084 | 0.016 |
| Tetraspanin 4 | BC000389 | 209263_x_at | 643.62 | 499.93 | -1.29 | 0.39 | 0.048 | 269.14 | -2.39 | 0.0075 | 0.073 |
| Tetraspanin 4 | AF054841 | 209264_s_at | 278.68 | 167.13 | -1.67 | 0.0035 | 0.012 | 127.69 | -2.18 | 0.00027 | 0.049 |
| Tetraspanin 5 | AF065389 | 209890_at | 332.73 | 205.27 | -1.62 | 0.0023 | <0.01 | 132.54 | -2.51 | 0.000034 | 0.016 |
| Thioredoxin interacting protein | AA812232 | 201008_s_at | 512.89 | 165.04 | -3.11 | 0.0063 | <0.01 | 132.68 | -3.87 | 0.0025 | 0.049 |
| Thioredoxin interacting protein | AI439556 | 201009_s_at | 507.71 | 190.82 | -2.66 | 0.0088 | <0.01 | 119.41 | -4.25 | 0.00097 | 0.016 |
| Thioredoxin interacting protein | NM_006472 | 201010_s_at | 535.81 | 187.88 | -2.85 | 0.0059 | <0.01 | 144.60 | -3.71 | 0.00094 | 0.028 |
| Thioredoxin reductase 1 | NM_003330 | 201266_at | 729.54 | 393.71 | -1.85 | 0.013 | <0.01 | 293.69 | -2.48 | 0.0035 | 0.028 |
| Three prime repair exonuclease 1 | NM_016381 | 205875_s_at | 127.55 | 24.89 | -5.13 | 0.00059 | <0.01 | 20.51 | -6.22 | 0.00041 | 0.073 |
| Three prime repair exonuclease 1 | AJ243797 | 34689_at | 146.34 | 32.47 | -4.51 | 0.00083 | 0.14 | 34.28 | -4.27 | 0.00095 | 0.073 |
| Tissue factor pathway inhibitor | J03225 | 209676_at | 265.88 | 86.55 | -3.07 | 0.018 | 0.012 | 107.35 | -2.48 | 0.032 | 0.13 |
| Tissue factor pathway inhibitor | AF021834 | 210664_s_at | 293.97 | 105.52 | -2.79 | 0.012 | <0.01 | 145.21 | -2.02 | 0.032 | 0.22 |
| Tissue factor pathway inhibitor | BF511231 | 213258_at | 421.90 | 116.18 | -3.63 | 0.01 | <0.01 | 166.21 | -2.54 | 0.025 | 0.13 |
| TNFAIP3 interacting protein 1 | NM_006058 | 207196_s_at | 386.23 | 158.71 | -2.43 | 0.00083 | <0.01 | 106.78 | -3.62 | 0.00022 | 0.016 |
| TRAF3 interacting protein 2 | AL008730 | 215411_s_at | 234.00 | 175.27 | -1.34 | 0.15 | 0.41 | 78.19 | -2.99 | 0.0014 | 0.073 |
| Transcription factor 21 | NM_003206 | 204931_at | 283.95 | 51.58 | -5.51 | 0.039 | 0.022 | 20.97 | -13.54 | 0.019 | 0.13 |
| Transforming growth factor, beta receptor II | D50683 | 208944_at | 579.73 | 300.49 | -1.93 | 0.000001 | <0.01 | 243.11 | -2.38 | 0.000001 | <0.01 |
| Transient receptor potential cation channel, subfamily A, member 1 | AA502609 | 217590_s_at | 588.36 | 111.31 | -5.29 | 0.0095 | <0.01 | 54.31 | -10.83 | 0.0041 | 0.049 |
| Transketolase | BF696840 | 208699_x_at | 292.41 | 240.65 | -1.22 | 0.46 | 0.41 | 117.75 | -2.48 | 0.0059 | 0.073 |
| Transketolase | L12711 | 208700_s_at | 448.92 | 345.93 | -1.30 | 0.27 | 0.048 | 208.80 | -2.15 | 0.0012 | 0.049 |
| Transmembrane protein 123 | BG538627 | 211967_at | 356.75 | 204.76 | -1.74 | 0.00052 | <0.01 | 170.54 | -2.09 | 0.0043 | 0.028 |
| Transmembrane protein 140 | NM_018295 | 218999_at | 156.91 | 20.11 | -7.80 | 0.00006 | <0.01 | 17.05 | -9.20 | 0.000054 | 0.049 |
| Transmembrane protein 176A | NM_018487 | 218345_at | 138.95 | 7.60 | -18.27 | 0.026 | 0.022 | 6.48 | -21.45 | 0.025 | 0.22 |
| Transmembrane protein 176B | NM_014020 | 220532_s_at | 235.62 | 9.53 | -24.73 | 0.049 | 0.022 | 9.01 | -26.15 | 0.049 | 0.22 |
| Transmembrane protein 47 | AI803181 | 209655_s_at | 313.65 | 151.74 | -2.07 | 0.015 | 0.012 | 146.70 | -2.14 | 0.011 | 0.13 |
| Transmembrane protein 50A | NM_014313 | 217766_s_at | 222.60 | 145.56 | -1.53 | 0.0082 | 0.26 | 100.89 | -2.21 | 0.000062 | 0.028 |
| Transporter 1, ATP-binding cassette, sub-family B | NM_000593 | 202307_s_at | 326.14 | 89.73 | -3.63 | 0.00079 | <0.01 | 56.49 | -5.77 | 0.00036 | 0.016 |
| Tripartite motif-containing 14 | NM_014788 | 203148_s_at | 160.01 | 32.32 | -4.95 | 0.000001 | <0.01 | 15.68 | -10.20 | 0.000001 | 0.0096 |
| Tripartite motif-containing 22 | AA083478 | 213293_s_at | 1052.31 | 178.30 | -5.90 | 0.000037 | <0.01 | 127.76 | -8.24 | 0.000041 | <0.01 |
| Tripartite motif-containing 38 | AU157590 | 203567_s_at | 155.95 | 57.72 | -2.70 | 0.0021 | 0.14 | 53.46 | -2.92 | 0.0024 | 0.13 |
| Tripartite motif-containing 8 | NM_030912 | 221012_s_at | 368.69 | 179.84 | -2.05 | 0.000089 | <0.01 | 189.11 | -1.95 | 0.00099 | 0.028 |
| Tripeptidyl peptidase I | BG231932 | 200742_s_at | 274.72 | 172.71 | -1.59 | 0.11 | 0.048 | 125.03 | -2.20 | 0.0039 | 0.073 |
| Tryptophanyl-tRNA synthetase | M61715 | 200628_s_at | 619.63 | 211.52 | -2.93 | 0.000002 | <0.01 | 213.84 | -2.90 | 0.000007 | <0.01 |
| Tryptophanyl-tRNA synthetase | NM_004184 | 200629_at | 645.60 | 197.00 | -3.28 | 0.000041 | <0.01 | 209.18 | -3.09 | 0.000051 | <0.01 |
| Tumor necrosis factor (ligand) superfamily, member 10 | NM_003810 | 202688_at | 139.35 | 15.74 | -8.85 | 0.028 | 0.022 | 11.39 | -12.24 | 0.024 | 0.22 |
| Tumor necrosis factor receptor superfamily, member 11b | BF433902 | 204932_at | 233.73 | 58.34 | -4.01 | 0.0011 | <0.01 | 34.50 | -6.78 | 0.00046 | 0.049 |
| Tumor necrosis factor receptor superfamily, member 11b | NM_002546 | 204933_s_at | 1231.11 | 277.60 | -4.43 | 0.0015 | <0.01 | 138.16 | -8.91 | 0.00062 | <0.01 |
| Tumor necrosis factor receptor superfamily, member 21 | NM_016629 | 218856_at | 247.55 | 93.51 | -2.65 | 0.004 | <0.01 | 74.69 | -3.31 | 0.002 | 0.073 |
| Tumor necrosis factor, alpha-induced protein 2 | NM_006291 | 202510_s_at | 404.24 | 63.56 | -6.36 | 0.000003 | <0.01 | 22.56 | -17.92 | 0.000003 | <0.01 |
| Tumor necrosis factor, alpha-induced protein 3 | AI738896 | 202643_s_at | 148.03 | 38.01 | -3.89 | 0.001 | <0.01 | 11.95 | -12.39 | 0.00025 | 0.049 |
| Tumor necrosis factor, alpha-induced protein 3 | NM_006290 | 202644_s_at | 237.81 | 59.81 | -3.98 | 0.000016 | <0.01 | 22.30 | -10.67 | 0.000007 | <0.01 |
| Tumor necrosis factor, alpha-induced protein 6 | AW188198 | 206025_s_at | 1047.49 | 323.01 | -3.24 | 0.022 | <0.01 | 598.21 | -1.75 | 0.13 | 0.29 |
| Tumor necrosis factor, alpha-induced protein 6 | NM_007115 | 206026_s_at | 847.28 | 303.41 | -2.79 | 0.0046 | <0.01 | 513.63 | -1.65 | 0.079 | 0.13 |
| Tumor necrosis factor, alpha-induced protein 8 | NM_014350 | 208296_x_at | 259.86 | 89.03 | -2.92 | 0.00048 | <0.01 | 61.92 | -4.20 | 0.00023 | 0.028 |
|  |  |  |  | **SSc-ILD** | | | | **IPF** | | | |
| **Annotation** | **Accession** | **Probe Set ID** | **Control mean** | **Ssc-ILD mean** | **Fold change** | **p value** | **q-value(%)** | **IPF mean** | **Fold change** | **p value** | **q-value(%)** |
| Tumor necrosis factor, alpha-induced protein 8 | BC005352 | 210260_s_at | 242.60 | 82.44 | -2.94 | 0.000064 | <0.01 | 63.96 | -3.79 | 0.000065 | 0.016 |
| Twist homolog 1 (Drosophila) | X99268 | 213943_at | 288.60 | 151.32 | -1.91 | 0.053 | 0.0303 | 49.52 | -5.83 | 0.0027 | 0.049 |
| Two pore segment channel 1 | NM_017901 | 217914_at | 171.94 | 112.39 | -1.53 | 0.0032 | 0.34 | 71.12 | -2.42 | 0.0074 | 0.049 |
| Ubiquitin specific peptidase 18 | NM_017414 | 219211_at | 134.72 | 26.59 | -5.07 | 0.0013 | <0.01 | 25.53 | -5.28 | 0.0013 | 0.073 |
| Ubiquitin-conjugating enzyme E2L 6 | NM_004223 | 201649_at | 770.87 | 136.41 | -5.65 | 0.000012 | <0.01 | 102.31 | -7.53 | 0.000012 | <0.01 |
| UDP-Gal:betaGlcNAc beta 1,4- galactosyltransferase, polypeptide 5 | BF691447 | 221484_at | 158.07 | 91.74 | -1.72 | 0.028 | 0.401 | 51.62 | -3.06 | 0.0016 | 0.13 |
| UDP-Gal:betaGlcNAc beta 1,4- galactosyltransferase, polypeptide 5 | AL035683 | 221485_at | 159.15 | 90.93 | -1.75 | 0.043 | 0.401 | 58.64 | -2.71 | 0.0023 | 0.13 |
| UDP-glucose ceramide glucosyltransferase | NM_003358 | 204881_s_at | 614.44 | 236.46 | -2.60 | 0.00033 | <0.01 | 149.43 | -4.11 | 0.000085 | <0.01 |
| UDP-glucose ceramide glucosyltransferase | AI378044 | 221765_at | 148.96 | 57.13 | -2.61 | 0.00016 | 0.14 | 39.16 | -3.80 | 0.00012 | 0.049 |
| Vascular cell adhesion molecule 1 | NM_001078 | 203868_s_at | 835.20 | 32.17 | -25.96 | 0.0049 | <0.01 | 16.38 | -51.00 | 0.0044 | 0.028 |
| Vascular endothelial growth factor C | U58111 | 209946_at | 613.42 | 324.15 | -1.89 | 0.0084 | <0.01 | 132.64 | -4.62 | 0.000014 | <0.01 |
| Vesicle amine transport protein 1 homolog (T. californica) | BC001913 | 208626_s_at | 431.45 | 369.63 | -1.17 | 0.64 | 0.41 | 194.20 | -2.22 | 0.012 | 0.073 |
| Vesicle-associated membrane protein 5 | NM_006634 | 204929_s_at | 349.85 | 97.17 | -3.60 | 0.00016 | <0.01 | 84.62 | -4.13 | 0.00016 | 0.016 |
| V-rel reticuloendotheliosis viral oncogene homolog A (avian) | NM_021975 | 201783_s_at | 231.54 | 167.59 | -1.38 | 0.013 | 0.401 | 98.32 | -2.35 | 0.000043 | 0.049 |
| Wilms tumor 1 associated protein | NM_004906 | 203137_at | 718.00 | 188.21 | -3.81 | 0.000007 | <0.01 | 111.01 | -6.47 | 0.000004 | <0.01 |
| Wilms tumor 1 associated protein | BC000383 | 210285_x_at | 438.40 | 122.84 | -3.57 | 0.000002 | <0.01 | 71.88 | -6.10 | 0.000002 | <0.01 |
| X (inactive)-specific transcript (non-protein coding) | AV699347 | 214218_s_at | 144.69 | 123.44 | -1.17 | 0.52 | 0.41 | 11.99 | -12.07 | 0.000003 | 0.016 |
| X (inactive)-specific transcript (non-protein coding) | AA628440 | 221728_x_at | 131.83 | 116.94 | -1.13 | 0.63 | 0.41 | 5.86 | -22.50 | 0.000001 | 0.016 |
| Xeroderma pigmentosum, complementation group C | D21089 | 209375_at | 213.50 | 113.26 | -1.89 | 0.011 | 0.022 | 64.06 | -3.33 | 0.00043 | 0.049 |
| XIAP associated factor 1 | NM_017523 | 206133_at | 190.99 | 35.58 | -5.37 | 0.00044 | <0.01 | 21.13 | -9.04 | 0.00026 | 0.049 |
| Zinc finger CCCH-type containing 12A | NM_025079 | 218810_at | 159.69 | 68.56 | -2.33 | 0.00059 | 0.14 | 39.20 | -4.07 | 0.000052 | 0.049 |
| Zinc finger protein 36, C3H type-like 2 | U07802 | 201368_at | 869.53 | 279.30 | -3.11 | 0.000001 | <0.01 | 178.87 | -4.86 | 0.000001 | <0.01 |
| Zinc finger, MYM-type 6 | NM_007167 | 219924_s_at | 184.18 | 87.01 | -2.12 | 0.000056 | 0.094 | 81.60 | -2.26 | 0.00005 | 0.073 |

**Additional file 4. Summary of differentially expressed genes in SSc-ILD and IPF.** Word file, .txt extension.

This data set contains all of the genes up- or down- regulated (according to the criteria described in the methods) in fibroblasts from at least one disease group compared to control fibroblasts. Included are p-values from dChip analysis and q-values from SAM analysis.
